# Remote assessment of the fate of phytoplankton in the Southern Ocean sea-ice zone

**DOI:** 10.1038/s41467-020-16931-0

**Published:** 2020-06-19

**Authors:** Sébastien Moreau, Philip W. Boyd, Peter G. Strutton

**Affiliations:** 1 Norwegian Polar Institute, Fram Centre, PO Box 6606 Langnes, NO-9296 Tromsø, Norway; 2 Institute for Marine and Antarctic Studies, University of Tasmania, Hobart, TAS 7001 Australia; 3 Australian Research Council Centre of Excellence for Climate Extremes, University of Tasmania, Hobart, TAS 7001 Australia

**Keywords:** Carbon cycle, Element cycles, Marine biology

## Abstract

In the Southern Ocean, large-scale phytoplankton blooms occur in open water and the sea-ice zone (SIZ). These blooms have a range of fates including physical advection, downward carbon export, or grazing. Here, we determine the magnitude, timing and spatial trends of the biogeochemical (export) and ecological (foodwebs) fates of phytoplankton, based on seven BGC-Argo floats spanning three years across the SIZ. We calculate loss terms using the production of chlorophyll—based on nitrate depletion—compared with measured chlorophyll. Export losses are estimated using conspicuous chlorophyll pulses at depth. By subtracting export losses, we calculate grazing-mediated losses. Herbivory accounts for ~90% of the annually-averaged losses (169 mg C m^−2^ d^−1^), and phytodetritus POC export comprises ~10%. Furthermore, export and grazing losses each exhibit distinctive seasonality captured by all floats spanning 60°S to 69°S. These similar trends reveal widespread patterns in phytoplankton fate throughout the Southern Ocean SIZ.

## Abstract (web summary)

Satellites can observe marine phytoplankton, but observations are sparse in seasonally dark, cloudy environments like the Southern Ocean. These authors use Argo floats to track the fate of phytoplankton blooms off Antarctica and determine 10% of biomass is exported, while 90% is prey to grazing.

## Introduction

Phytoplankton in the Southern Ocean produce new particles, driving the biological carbon pump that plays a disproportionately important role in global climate on a range of time scales^1,2^. Phytoplankton also supply energy to support Antarctic krill, the most abundant animal on the planet by mass^3^, and the link to apex predators. In the Southern Ocean, satellite remote-sensing reveals that the sea-ice zone (SIZ) accounts for ~15% of the basin-scale primary production^4^. The productivity of the SIZ also plays a strong role in setting the magnitude of the downward particle flux, via the rapid sinking of sea-ice edge blooms^5^, and sea ice is a critical habitat for overwintering krill^6^. At decadal time scales, the fate of phytoplankton is changing in the SIZ due to environmental forcing^7,8^. A climate-change related reduction in sea-ice cover could have dramatic consequences for biogeochemistry and ecology if the fate of phytoplankton is altered^9–11^.

**Fig. 1:**
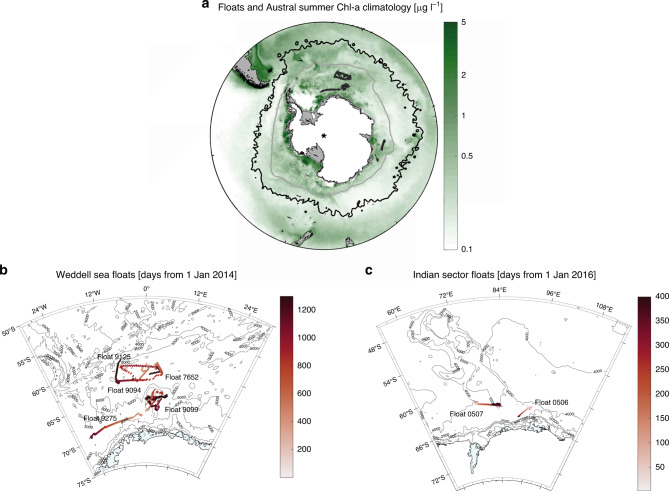
Floats trajectories in the sea-ice zone. Maps of the float trajectories overlying the MODIS chlorophyll austral summer climatology (2002–2018) in **a** and as a function of profile days in **b** the Weddell Sea and **c** the Indian sector of the Southern Ocean. In **a**, the grey line is the climatological sea-ice extent in September (1978−2017) and the black line is the northern jet of the sub-Antarctic Front (SAF-N) as defined in Ref. ^70^.

Biogeochemical (BGC) Argo floats are a relatively recent technological development that provide vertical profiles of chlorophyll (chl) and particles (both proxies for phytoplankton biomass), along with temperature, salinity, pH, dissolved oxygen and nitrate concentration^12^. These observations are particularly valuable for polar oceans, where fewer in situ observations have been made. Floats can also sense the water column during the polar night and under the sea ice where satellites cannot view^13^, providing unique time series of phytoplankton growth and particulate organic carbon (POC) export dynamics^14^. Chl concentration is routinely used to derive primary production remotely from satellite^15,16^ but little effort has focused on the logical next step—assessing the fate of phytoplankton blooms^17^. Information on the fate of phytoplankton is valuable as it helps to partition losses due to physical (advection), biogeochemical (export) and ecological (grazing and mortality as part of pelagic foodwebs) processes^18^. The objective of this study is to assess patterns in the biogeochemical and ecological fates of primary producers as revealed through careful analysis of BGC-Argo multi-sensor datasets for seven widely distributed SOCCOM (Southern Ocean Carbon and Climate Observations and Modelling) floats that drifted across the SIZ of the Weddell Sea and the Indian Ocean sector of the Southern Ocean, between 60° S and 69° (see “Methods” and Fig. 1). Our findings on the distinctive seasonality of the fate of phytoplankton represent a step forward in our understanding of drivers of biogeochemistry and ecology at high latitudes, where few continuous time series exist in open water^19^ or in the dynamic SIZ^20^.

## Results

### Calculating chlorophyll production and losses

The development of an approach to tease apart the fate of chl relies first on calculating its potential production (i.e., de novo synthesis), comparing this with measured chl, then assessing the cumulative losses due to advection, vertical export, grazing and mortality^18^. The first step relies on knowledge that phytoplankton photosynthesis produces chl via the consumption of NO_3_^−^ at known rates. For phytoplankton and sea-ice algae the ratio of chl synthesis to nitrate uptake (chl:N) varies between 0.34 and 2.47 µg chl:µmol N as reported in field studies and laboratory cultures across sites ranging from polar to tropical waters^21–26^ (a sensitivity analysis of the method to the chl:N ratio is presented in the “Methods”). A recent biogeochemical budget for the Amundsen Sea Polynya^23^ provides a very detailed, multi-station time series of nitrate depletion and concurrent accumulation of chl during a rapidly evolving bloom (average ratio 1.75 ± 0.4 µg chl:µmol N) from a polynya analogous to the SIZ we studied using BGC-Argo. Thus, from nitrate drawdown and this reference value of chl:N = 1.75 µg chl:µmol N, we calculate potential chl synthesis without any losses, so long as ocean physics enables a comparison of consecutive nitrate profiles derived from BGC-Argo.

**Fig. 2:**
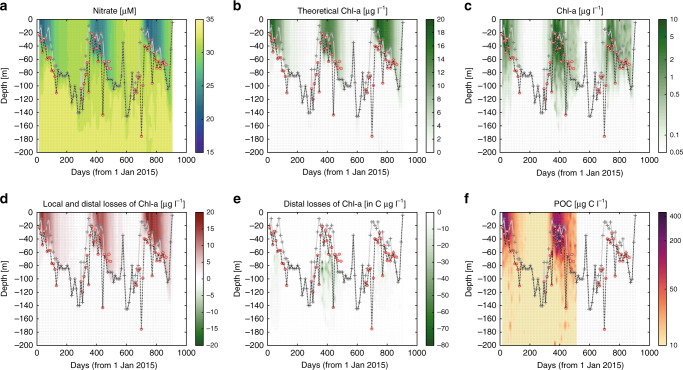
Biogeochemical observations for float #9099 in the Weddell Sea. Concentration of **a** nitrate (µM), **b** theoretical maximum chl (µg l^−1^), **c** measured chl (µg l^−1^), **d** total losses (µg chl l^−1^), **e** distal losses (µg C l^−1^), and **f** particulate organic carbon (POC, µg C l^−1^) in the upper 200 m. Thick dashed line indicates the deepest of the euphotic zone depth (Zeu, red circles) defined from Ref. ^59^, and the mixed layer depth (grey crosses and grey line) defined from Ref. ^58^, as the depth at which density increased by 0.01 kg m^−3^ compared with density at the surface. Winter mixed layer across all seven floats varied from 90 to 175 m deep, averaging 125 ± 6 m. Thin dashed lines indicate float profiles.

We were able to rule out advection as a loss term for chl and nitrate, because changes in physical properties were at least an order of magnitude smaller than published criteria for consideration of contiguous profiles (see “Methods” and Ref. ^27^). Each winter, nutrient inventories (here nitrate, NO_3_^−^) are reset in the upper ocean by deep vertical mixing. That is, the concentration over the upper few hundred metres becomes almost uniform and equivalent to the deep concentration. Using a chl:*N* ratio of 1.75 µg chl:µmol N for our reference value and the salinity-normalized NO_3_^−^ drawdown relative to the closest winter NO_3_^−^ profile (ΔNO_3_^−^, Fig. 2a), we can calculate the concentration of chl (chl*) that should be present in the absence of losses (Fig. 2b):

1 $${\mathrm{chl}}^ \ast = 1.75 \times \Delta {\mathrm{NO}}_3^ -.$$

Chl concentration is measured by a calibrated in vivo fluorescence sensor on the BGC-Argo float, corrected for nonphotochemical quenching^28^ and assumed to represent biomass^12^ after losses:

2 $${\mathrm{chl}} = {\mathrm{chl}}^\ast - {\mathrm{total}}\;{\mathrm{losses}}.$$

The difference between chl* and the observed chl (Fig. 2c) represents a proxy for the total losses of phytoplankton biomass (Fig. 2d):

3 $${\mathrm{total}}\;{\mathrm{losses}} = {\mathrm{chl}}^\ast - {\mathrm{chl}}.$$

Total losses can be partitioned into local and distal losses, where distal losses are downward export, because we eliminated horizontal advection:

4 $${\mathrm{total}}\;{\mathrm{losses}} = {\mathrm{local}}\;{\mathrm{losses}} + {\mathrm{export}}.$$

The downward export of chl as phytodetritus (i.e., phytoplankton aggregates and senescent cells^29^) is conspicuous in BGC-Argo profiles below the export depth (i.e., the deeper of either the surface mixed layer, ML, or the euphotic zone depth, Zeu; see “Methods”) where it corresponds to higher (i.e., excess) chl concentrations than that expected from the negligible observed NO_3_^−^ drawdown at these depths (Fig. 2e). The export of phytodetritus is first observed below the export depth in early spring and is coincident with observations of POC export from the BGC-Argo backscattering sensors (Fig. 2f). This early export of phytodetritus and POC, following the stratification of the water column, was consistently observed among all seven floats (Supplementary Figs. 1 and 2), in agreement with previous studies that showed that shallow transient stratification close to spring led to more favourable conditions for primary production and carbon export in the Southern Ocean^14^. Losses of chl due to downward export were calculated using conspicuous excess chl pulses at depth (between the export depth and 175 m, the deepest winter mixed layer estimated from all seven floats, see “Methods”).

After calculating phytodetritus downward export, local losses were calculated as the difference between total losses (between the surface and 175 m) and downward export (between the export depth and 175 m):

5 $${\mathrm{local}}\;{\mathrm{losses}} = {\mathrm{total}}\;{\mathrm{losses}} - {\mathrm{export}}.$$

Local losses of chl can be due to physiological and ecological processes: mortality and grazing, respectively (Fig. 2d). Mortality (defined here as intrinsically driven mortality in response to environmental stresses such as nutrient starvation^30^) was calculated from published oceanic rates used in modelling^31^ and subtracted from local losses, leaving grazing. We have included viral lysis in the grazing term in these calculations, due to the dearth of knowledge available to tease grazing and lysis apart for the SIZ.

6 $${\mathrm{grazing}} = {\mathrm{local}}\;{\mathrm{losses}}-{\mathrm{mortality}}.$$

**Fig. 3:**
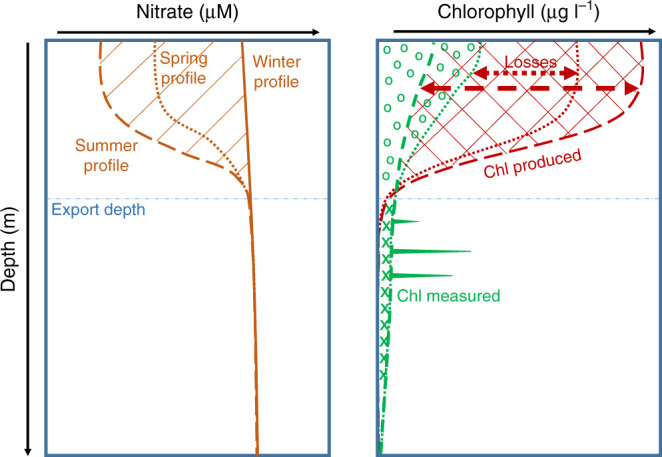
Schematic of steps to determine the fate of phytoplankton. After ruling out advection by comparing the water column physical properties between consecutive profiles, we can compare a spring or summer nitrate profile to the winter nitrate profile (left panel). Second, based on the observed nitrate depletion (the hatched area in the left panel), we can calculate the chlorophyll produced (right panel, dotted lines correspond to spring in the both panel, dashed lines to summer). Third, by comparing the chlorophyll produced to the chlorophyll measured, we get the cumulative loss terms (red arrows in the right panel). Loss terms associated with different parts of the water column. Below the export depth, the excess chlorophyll reveals the export of phytodetritus, whereas herbivory mainly takes place between the surface and the export depth. The accumulation of phytoplankton biomass takes place between the surface and the export depth.

Finally, the phytoplankton biomass accumulation was calculated from positive changes in integrated observed chl^15^. Chl losses and biomass accumulation were converted to carbon units to assess the contribution of phytodetritus POC export to downward POC export (see “Methods”). This approach is described schematically in Fig. 3.

Following previous work that estimated POC export from sensors mounted on lagrangian floats^32,33^, we estimate POC export from the POC increment below the export depth and down to 175 m between consecutive profiles. The downward POC export includes phytodetritus and particles transformed by the foodweb such as faecal pellets^34^ (see “Methods”), and is calculated over the same depth stratum as phytodetritus POC export.

Other studies (Dall’Olmo et al.^33^ and Briggs et al.^35^) measured the sinking of small particles through the mesopelagic layer using backscattering sensors mounted on BGC-Argo floats. Using this approach, Briggs et al.^35^ highlighted that particulate fragmentation was responsible for 49 ± 22% of the observed flux loss between 100 and 1000 m. Our method, using BGC-Argo sensors to estimate carbon export, can not account for the bacterial remineralization of particulates into dissolved organic carbon (DOC). In addition, large backscattering spikes might be caused by the presence of zooplankton as shown by Bishop and Wood^36^, which would lead us to overestimate POC export.

### Biogeochemical losses of chlorophyll

**Fig. 4:**
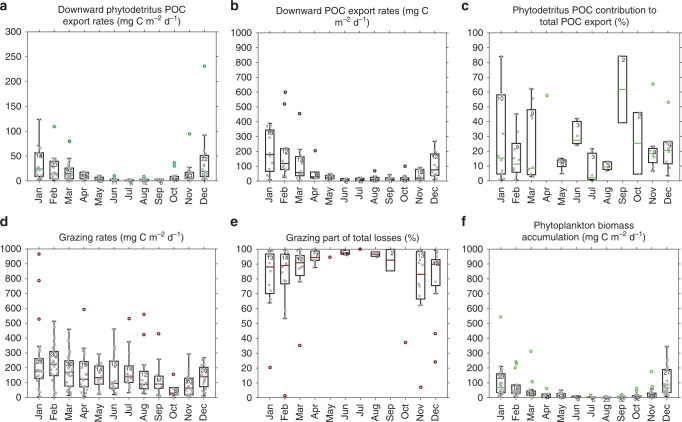
Seasonal evolution of phytoplankton fates. Box plot of integrated **a** phytodetritus POC export (mg C m^−2^ d^−1^), **b** POC export (mg C m^−2^ d^−1^), **c** phytodetritus POC contribution to total POC export (%), **d** grazing (mg C m^−2^ d^−1^), **e** grazing part of total losses (%) and **f** phytoplankton biomass accumulation (mg C m^−2^ d^−1^). The box plot shows the median, the 50% central region, and the functional limits ([Q1 − 1.5 × IQR, Q3 + 1.5 × IQR], where Q1 and Q3 are the 25th and 75th percentiles respectively, and IQR is the interquartile range). Data are plotted as crosses and outliers as circles. The sample size is indicated for each box.

Phytodetritus POC export takes place from October to April, and is at its highest from December to March (Fig. 4a). The maximum measured export of phytodetritus POC was 413 mg C m^−2^ d^−1^ early in the production season, on December 28, 2016, by float #9275 in the south of the Weddell Sea (Supplementary Fig. 1). Maximum downward POC export was 600 mg C m^−2^ d^−1^ measured on February 2, 2016, by float #0506 (Fig. 4b), and was in the range of previous maximum estimates from the open Southern Ocean to the SIZ (1090 mg C m^−2^ d^−1^; Ref. ^37^). Pooling the seven BGC-Argo float datasets we studied, POC export follows phytodetritus POC export through the phytoplankton production season, and is highest from December to March (Fig. 4b). However, compared with phytodetritus POC export, POC export starts a little later, in November compared with October.

Our analysis provides seasonal trends in the contribution of phytodetritus to downward POC export. Where POC and phytodetritus POC export coincide, we find that the export of phytodetritus accounts for 24% on average of the total export of POC (Fig. 4c and Supplementary Fig. 3). However, the annually averaged POC and phytodetritus POC export calculated for all the BGC-Argo floats are 79 and 19 mg C m^−2^ d^−1^, respectively, which considers all seasons. Hence the contribution of phytodetritus over the annual cycle is ~19%. This contribution is variable during the winter months (with few estimates from May to October) but consistent throughout the productive season, although slightly higher at the beginning of the production season (November–December) than at the end (January–March, Fig. 4c). At the onset of the production season, the contribution of grazers to POC export via faecal pellets or vertical migration is expected to be lower as grazers may still be overwintering at depth^38^.

### Ecological fate of phytoplankton

After calculating the direct export of chl as phytodetritus, grazing is determined by difference since physical losses are negligible. Chl losses are mainly observed between the surface and the export depth consistent with herbivory (Fig. 2d). However, to be consistent with our phytodetritus POC export calculations, losses due to grazing were integrated from the surface to 175 m depth. The maximum grazing rate was 965 mg C m^−2^ d^−1^, measured on January 13, 2015 by float #7652 in the Weddell Sea (Fig. 4d). Previously, modelling studies^39^ have calculated upper bound grazing rates of 400–700 mg C d^−1^ for the coastal and open Southern Ocean. Pooling all BGC-Argo floats considered here, we find annually averaged grazing rates of 169 mg C m^−2^ d^−1^ (Fig. 4d). This analysis is the first basin scale, observational estimate of grazing throughout the SIZ of the Southern Ocean. In comparison, we observe relatively little accumulation of phytoplankton in the SIZ (Fig. 4f). The annually averaged phytoplankton biomass accumulation calculated for all the BGC-Argo floats was 40 mg C m^−2^ d^−1^. The maximum phytoplankton biomass accumulation was 543 mg C m^−2^ d^−1^, measured by float #9099 in the Weddell Sea on January 3, 2016.

Pooling all float datasets, we can also derive the seasonal influence of grazing on chl. An increase in herbivory is observed from October to December, while higher grazing pressure is fairly constant throughout the second part of the growing season, January–June, 3 months after the main downward export events (Fig. 4d). Grazing decreases during winter (July–October) but does not fall to zero, consistent with observations that krill and other zooplankton feed throughout winter for their survival^40^.

For all float datasets, where phytodetritus POC export events and grazing rates are coincident, grazing contributes 83% of total chl losses, on average. However, a comparison of the annually averaged grazing rates (169 mg C m^−2^ d^−1^) to the annually averaged phytodetritus POC export (19 mg C m^−2^ d^−1^) for all float datasets takes the contribution of grazing to total chl losses to ~90%. The remaining 10% is downward export of phytodetritus. This trend is consistent throughout the year for all floats, except in November, at the beginning of the production season, when the impact of grazing is slightly lower (Fig. 4e). This is consistent with the higher contribution of phytodetritus to total export at the beginning of the production season. Furthermore, the dominance of grazing throughout most of the year confirms the primary role of zooplankton in controlling the fate of phytoplankton in the Southern Ocean^41,42^.

### Sensitivity of the method to the chl:N ratio

To account for the potential variability in the chl:N ratio, we ran a detailed sensitivity analysis of our approach to the chl:*N* ratio (see Methods). The results of the sensitivity analysis for all floats is given in Table 1. We find that the average export of phytodetritus for all floats is 22 ± 3, 19 ± 3 and 18 ± 3 mg C d^−1^ for chl:N ratios of 1.25, 1.75 and 2.5 µg chl:µmol N, respectively. Similarly, average grazing for all floats is 126 ± 7, 169 ± 10 and 233 ± 13 mg C d^−1^ for chl:N ratios of 1.25, 1.75 and 2.5 µg chl:µmol N, respectively. Thus, the contribution of grazing to total chl losses is 85, 90 and 93% for varying chl:N ratios of 1.25, 1.75 and 2.5 µg chl:µmol N, confirming the prevalence of grazing on chl losses in the SIZ of the Southern Ocean.

**Table 1:** Results of the sensitivity analysis of the method to the chl:N ratio (µg chl:µmol N).

	Chl:N = 1.25			Chl:N = 1.75			Chl:N = 2.5		
	Phytodetritus	Grazing	% grazed	Phytodetritus	Grazing	% grazed	Phytodetritus	Grazing	% grazed
Float 7652									
Avg	11	165	94	10	224	96	10	315	97
Stdev	2	18		2	24		2	33	
Float 9094									
Avg	23	139	86	16	186	92	14	265	95
Stdev	9	22		6	31		4	45	
Float 9099									
Avg	33	119	78	24	170	88	23	227	91
Stdev	9	20		6	28		6	35	
Float 9125									
Avg	12	89	88	12	116	90	12	161	93
Stdev	3	11		3	14		3	20	
Float 9275									
Avg	45	121	73	38	157	81	36	223	86
Stdev	19	15		16	20		17	28	
Float 0506									
Avg	12	96	89	12	131	91	13	180	93
Stdev	3	22		3	24		3	32	
Float 0507									
Avg	24	123	83	25	156	86	24	204	89
Stdev	6	17		5	22		5	29	

Phytodetritus POC export and grazing average (Avg) and standard deviation (stdev) are given in mg C m^−2^ d^−1^. The contribution of grazing to the total fate of phytoplankton is given as % grazed.

## Discussion

**Fig. 5:**
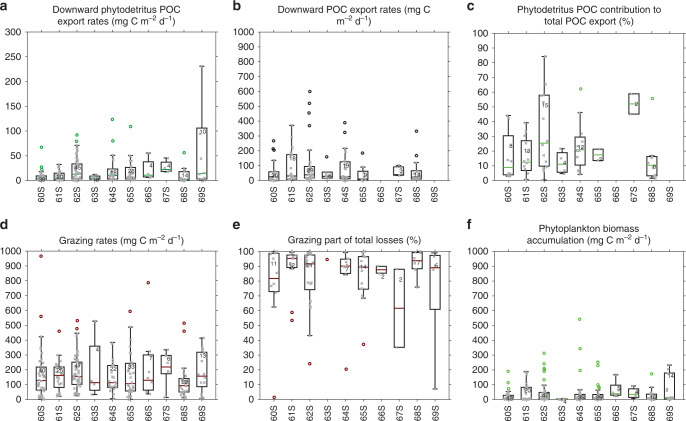
Latitudinal distribution of phytoplankton fates. Box plot of integrated **a** phytodetritus POC export (mg C m^−2^ d^−1^), **b** POC export (mg C m^−2^ d^−1^, no export values were reported at 66 and 69° S), **c** phytodetritus POC contribution to total POC export (%), **d** grazing (mg C m^−2^ d^−1^), **e** grazing part of total losses (%) and **f** phytoplankton biomass accumulation (mg C m^−2^ d^−1^). The box plot shows the median, the 50% central region, and the functional limits ([Q1 − 1.5 × IQR, Q3 + 1.5 × IQR], where Q1 and Q3 are the 25th and 75th percentiles respectively, and IQR is the interquartile range). Data are plotted as crosses and outliers as circles. The sample size is indicated for each box.

The seven floats we studied span the Weddell Sea and the Indian Ocean sector of the SIZ between 60 and 69° S (Fig. 1). Despite this wide range of locales sampled, we find no statistically significant differences in the magnitude of phytodetritus POC export, POC downward export and grazing across the SIZ (Fig. 5). The only departure from this prevalent trend is higher phytodetritus POC export at 69° S (Fig. 5a). This event may be due to a combination of higher rates of phytoplankton biomass accumulation compared with the rest of the SIZ (Fig. 5f) but a grazing loss term that is comparable to other latitudes (Fig. 5d). We also find no statistically significant differences in the magnitude of phytodetritus POC export, POC export and grazing between sampling years (2015–2017, Supplementary Fig. 4), between floats (Supplementary Fig. 5), and between the SIZ areas studied: the Weddell Sea and Prydz Bay (Supplementary Fig. 6).

This evidence of widespread and consistent seasonality in the phytodetritus and herbivory loss terms is striking but has a number of plausible explanations. First, since this study is focused in the Southern Ocean SIZ, the major phytoplankton grazers are likely to be Antarctic krill, *Euphausia superba*^43^. The spatially uniform nature of the fate of blooms is inconsistent with the known patchy distribution of krill^3^. Hence, the uniformity suggests that on longer time scales the krill patchiness may be averaged out, by the lateral advection of krill patches^44^. Second, this study focuses on BGC-Argo floats that had trajectories within the latter two of the three major krill development areas which are: the Scotia Sea and Antarctic Peninsula; the eastern Weddell and Lazarev Seas; and the north of Prydz bay and the Kerguelen Plateau^45^. The constancy of chl loss terms due to herbivory across the SIZ could be linked to the ubiquitous grazing pressure of krill in these areas, which dominates losses^46^. Analysis of floats from outside of known krill development areas could confirm, or not, the extent to which phytoplankton losses are uniform across the SIZ. Third, our data may alternatively reflect that copepod herbivory dominates where krill herbivory is absent^47,48^. The early season dominance of phytodetritus POC export as a major loss term is probably due to the similarity in the drivers of primary productivity across the SIZ, where sea-ice melt in spring typically releases large amounts of dissolved and particulate iron to surface waters^49^. This nutrient delivery, coupled with a shallow meltwater- and temperature-driven mixed layer, fosters the sea-ice edge blooms in which phytoplankton growth is decoupled from grazing^50^.

The inaccessibility, over much of the annual cycle, of both the open water regions and the SIZ of the Southern Ocean has resulted in a limited vison of how the regional biogeochemistry and ecology function, with little known about how they interact. Satellite oceanography, using a range of sensors, has led to major advances in understanding the seasonality of phytoplankton blooms and how it can be linked to other remotely sensed environmental drivers such as satellite-derived sea-ice extent and upper ocean temperature^51^. However, satellite oceanography has for some sensors been hindered by the cloudiness that characterized much of the Southern Ocean. Our findings illustrate how profiling BGC-Argo floats can complement other platforms and potentially launch significant advances in our understanding in the same way that satellite ocean colour has since the 1990s. Given the complexity of assessing the status of this ecosystem and the carbon cycle, our approach enables quantification of the fates of phytoplankton in the SIZ on a short time scale (10 days) throughout the annual cycle, and moreover to map their seasonality across a range of diverse locales. In comparison, other estimates of net community production based on export or nitrate drawdown relative to winter^27^, are on a seasonal to annual time scale. Some of the most recent BGC-Argo floats are equipped with Underwater Vision Profilers (UVP6) which will allow counting organisms from bacteria to zooplankton^52^. The ability to discern the fate of SIZ phytoplankton is essential for understanding the current magnitude of, and future trends in the high-latitude biological pump and ecosystems.

## Methods

### The sea-ice zone

We studied seven SOCCOM floats that drifted across the SIZ of the Weddell Sea and the Indian Ocean sector of the Southern Ocean, between 60 and 69° S (Fig. 1a). The Weddell Sea is a large cyclonic gyre. Northeast of the Weddell Gyre, the Southwest Indian Ridge was identified as a major topographic feature where circumpolar deep water (CDW) travels southward through the Antarctic Circumpolar Current (ACC)^53^. South of the ACC, CDW is upwelled to the surface through wind-driven divergence and is modified on its path through the Weddell Gyre (then called Warm Deep Water, WDW^54^). Five BGC-Argo floats drifted across the open waters of the eastern Weddell Gyre (Fig. 1b), a region that was identified as a major carbon sink due to strong primary production^55^. The Kerguelen Plateau is a major obstacle on the eastward flow of the ACC. The bulk of the ACC passes north of the Kerguelen Plateau, while the remainder passes through the Fawn Trough (at ~56° S) or through the Princess Elizabeth Trough (at ~64° S), south of the Banzare Bank^56^ (Fig. 1c). Two BGC-Argo floats drifted in the highly productive SIZ, south of the Banzare bank^57^. The temperature and salinity sections of all seven floats are typical of ice-covered regions (Supplementary Figs. 7 and 8), with increases in mixed layer salinity under sea ice during winter and the classic thermal signature of winter water colder than −1 °C at 100 m deep^14^. The SOCCOM floats we studied park and drift at 1000 m, and descend to 2000 m before returning to the surface every 10 days.

### Criteria necessary to develop the algorithm

Changes in water mass properties in the 10 days window between profiles can rule out the comparison of sequential float profiles. Hence, we first assessed advection between consecutive profiles via a careful analysis of physical properties. Horizontal loss terms can be regarded as not significant if changes in physical properties are negligible. To successfully infer net community production from BGC-Argo floats in the Southern Ocean, Johnson et al.^27^ imposed three criteria: (1) that the salinity at 500 m did not change by more than 0.05 between two consecutive profiles, (2) that the latitude did not change by more than 5.5° and (3) that the longitude did not change by more than 8°. For all the BGC-Argo floats studied here, we found average (maximum) changes of 0.0013 (0.017) for salinity at 500 m, 0.07° (0.45°) for latitude, and 0.22° (1.1°) for longitude between two consecutive profiles. That is, the changes we observed were at least an order of magnitude smaller than the Johnson et al. criteria. Water column properties in the SIZ are, therefore, highly stable between successive CTD profiles, which makes it reasonable to compare the temporal evolution of biogeochemical properties and ignore horizontal and vertical advection as influential loss terms for chl. That is, we consider consecutive profiles as contiguous. Furthermore, we suggest that this method can be generalized to other oceanic regions as long as the changes in physical properties between consecutive profiles are minimal.

In addition, to estimate NO_3_^−^ drawdown, we used the salinity-normalized NO_3_^−^ profile to correct for dilution and concentration effects linked to sea-ice melt and formation in the SIZ.

### Export depth and horizon

Following Dall’Olmo et al.^33^, the export depth is taken as the deeper of either the surface mixed layer (ML, defined from Ref. ^58^ as the depth at which density increased by 0.01 kg m^−3^ compared with density at the surface) or the euphotic zone depth (Zeu, defined by Ref. ^59^ as the 2% light level and derived from sea surface chl concentration in Antarctic waters) when solar irradiance reaches the ocean surface (i.e., not during the polar night) as determined by year day and latitude. We acknowledge that other open water studies have used the critical depth rather than the euphotic zone depth as the upper limit below which to calculate downward export^14^. However, near the ice edge where the coverage of satellite-derived irradiance data is poor, we find that the euphotic zone reflects more accurately the depth over which particles may be created. In contrast, the critical depth may be a more powerful metric in open waters as previously shown by Bishop and Wood^14^.

We chose 175 m (the deepest winter mixed layer estimated from all seven floats) as the depth horizon to calculate export as recent studies demonstrate that the depth of the mixed layer in winter constrains downward export^60^. For example, a proportion of the organic matter that is exported below the export depth during the stratified summer months can be ventilated by deep winter mixing.

### Confirmation of deep excess chlorophyll as phytodetritus

A rationale is presented here for interpreting subsurface excess in chl as phytodetritus (such as aggregated senescent cells) sinking below the export depth. A few studies showed that diatoms may still be viable after ingestion and excretion by krill^61^, which could cast uncertainty on our assumption of observations of phytodetritus below the export depth. However, in the Indian sector of the Southern Ocean, in proximity to Crozet Island and the Kerguelen plateau, widely observed backscattering and fluorescence excess—that are conspicuous in the profiles below the export depth—were attributed to either faecal pellets or phytodetritus^29^. The authors argued that the senescence of phytoplankton does not alter the phytol chain of chl a, which leaves the fluorescence of the prophyrin ring intact. In contrast, zooplankton grazing removes the magnesium ions from chl a, which results in the accumulation of phaeopigments in faecal pellets. Therefore, we are confident that the positive chl anomalies we observed below the export depth were mainly composed of phytodetritus.

### Grazing rates estimates

Instantaneous grazing rates can be calculated from the profile-to-profile increments in local losses of chl. First, local losses are calculated as the difference between total chl losses and the export of phytodetritus below the export depth, that is, phytodetritus POC export (Eq. (5)). Phytoplankton mortality between the surface and the export depth^31^ is then subtracted from local losses to leave grazing (Eq. (6)).

To constrain this calculation, we further impose three criteria: (1) we only consider positive changes in local losses. Negative local losses would be caused by the input of nitrate between the surface and the export depth and yield negative grazing rates; (2) we only subtract positive changes in phytodetritus between the export depth and 175 m, because negative changes would represent export below 175 m and artificially increase grazing rates, and (3) we only calculate grazing rates when increments in local losses are higher than increments in phytodetritus below the export depth, as the contrary would yield negative grazing rates. The above-mentioned conditions reduce the number of grazing estimates, but they allow us to constrain grazing rates as close to reality as possible. A reasonable number of grazing estimates (*N* = 219) can be obtained via this method.

The production of faecal pellets by grazers is where carbon export and foodwebs interact^34^. Grazing will result in the destruction of chl and the repackaging of phytoplankton cells into aggregates or faecal pellets. To avoid double accounting of these chl losses, we made a simple distinction between the direct export of phytodetritus and grazing.

### Chlorophyll to carbon conversion

We used three conversion factors to convert chl losses and biomass accumulation to carbon: (1) a lower bound of 20 µg C/µg chl^62^ to constrain the minimum downward POC export associated with phytodetritus under saturating light and high nutrient supply, (2) a robust method to derive phytoplankton carbon from chl^63,64^ (referred to hereafter as T17 for Thomalla et al.^64^); and (3) the average POC:chl ratio (in µg C/µg chl) measured in the ML for each separate float (ML POC:chl ratios for all the BGC-Argo floats studied are shown in Supplementary Fig. 9). We used approach 2 from T17 for our reference value, since it was specifically derived from multiple Southern Ocean glider transects^64^. This yielded POC:chl ratios consistent with other approaches based on backscattering^65^, but it does not account for varying POC:chl ratios with environmental conditions.

### Sensitivity of the method to the chl:N ratio

We tested the sensitivity of the calculated maximum chl concentration to chl:N ratios ranging from 0.5 to 2.5 µg chl:µmol N (Supplementary Fig. 10). First, we found that a lower bound of 1.25 µg chl:µmol N was appropriate for our calculations, since below this limit, the estimated synthesized chl is consistently lower than the observed chl for 100 days at the beginning of the production season (days 300–400 in Supplementary Fig. 10a–c). This finding was consistent between all seven of the floats we studied. Furthermore, we chose 2.5 µg chl:µmol N as a reasonable upper bound for our sensitivity analysis given that it was among the highest reported chl:N values reported in field studies and laboratory cultures across sites ranging from polar to tropical waters^21–25^.

Next we selected an intermediate chl:N ratio of 1.75 µg chl:µmol N as our reference value. This selection was supported since it is the average chl:N ratio (1.75 ± 0.4 µg chl:µmol N) observed in the recent detailed Amundsen Sea Polynya (spanning 72.5–74° S)^23^, across a primary production gradient of integrated dissolved inorganic nitrogen drawdown (ΔDIN) of 27–740 mmol N m^−2^ and integrated chl from 74 to 828 mg m^−2^ (Supplementary Table 1). This detailed, multi-station study is ideal since it presents a time series of nitrate depletion and concurrent accumulation of chl during a rapidly evolving water column bloom, in the virtual absence of grazers^23^. There is, however, no such situation when grazers are completely absent, suggesting that our grazing estimates are possibly underestimated. Furthermore, the Amundsen Sea polynya has the highest primary production rates of all Antarctic polynyas^66^. Surrounding the Amundsen Sea polynya, the Dotson and Getz Ice Shelves are among the fastest melting glaciers of Antarctica^67^, and naturally fertilize the polynya^68^. Therefore, the source of dissolved iron is likely different between the Amundsen Sea polynya and the SIZ of the Southern Ocean, with possible effects on the chl:N ratio of phytoplankton. In comparison, the chl:N ratio reported from the SOFEX-S iron-amended mesoscale bloom in the SIZ (at 66 S) averaged 2.1 µg chl:µmol N, as reported by Coale et al.^26^ (their Table 1), and was therefore comparable to the chl:N ratio of 1.75 µg chl:µmol N we chose as reference value. In addition, the in situ chl:N ratio is likely to vary throughout the production season and among phytoplankton species^69^. A map of the chl:N ratio (µg chl:µmol N) measured in the Amundsen Sea polynya supports this variability (Supplementary Fig. 10d), with a decreasing ratio eastward, towards a more developed bloom^23^.

The results of the sensitivity analysis of our approach to the chl:N ratio (from 1.25 to 2.5 µg chl:µmol N) are presented in Table 1 (see “Results”).

### Reporting summary

Further information on research design is available in the Nature Research Reporting Summary linked to this article.

## Supplementary information

Supplementary Information

Peer Review File

## Data Availability

All SOCCOM data used in the present paper are available at https://soccom.princeton.edu.
