# Supplementary material for: Remote assessment of the fate of phytoplankton in the Southern Ocean sea-ice zone
Source: Nat Commun. 2020 Jun 19;11:3108. doi: 10.1038/s41467-020-16931-0 (PMC7305117; doi:10.1038/s41467-020-16931-0)
Supplement: Supplementary file 1 — Supplementary Information [file 41467_2020_16931_MOESM1_ESM.pdf]

Supplementary information for “Remote assessment of the fate of phytoplankton in the Southern Ocean sea ice zone” by Moreau et al.

Supplementary Table

Supplementary Table A1: Integrated ΔDIN (mmol N m^{-2}) and chlorophyll (mg m^{-2}) from the Amundsen polynya[10] and the corresponding chl:N ratio (µg chl:µmol N)

Station and CTD cast	Latitude (°)	Longitude (°)	ΔDIN	Chl *a*	chl:N
5 (3)	−73.97	−118.03	27	177	6.55
68 (87)	−71.86	−118.28	144	74	0.51
6 (4)	−73.18	−115.00	205	552	2.69
34 (31)	−72.96	−115.76	236	460	1.95
66 (85)	−72.74	−116.02	248	350	1.41
18 (14)	−73.00	−113.30	310	662	2.14
13 (9)	−73.57	−112.67	369	512	1.39
25 (21)	−73.12	−112.00	470	502	1.07
50 (65)	−73.42	−115.25	558	436	0.78
35 (46)	−73.28	−112.10	565	561	0.99
48 (61)	−73.70	−115.45	606	828	1.37
29 (26)	−73.35	−114.13	646	762	1.18
57 (72)	−73.71	−113.27	740	689	0.93
Average	-	-	394	505	1.77
S.E.	-	-	61	60	0.4

Supplementary Figures:

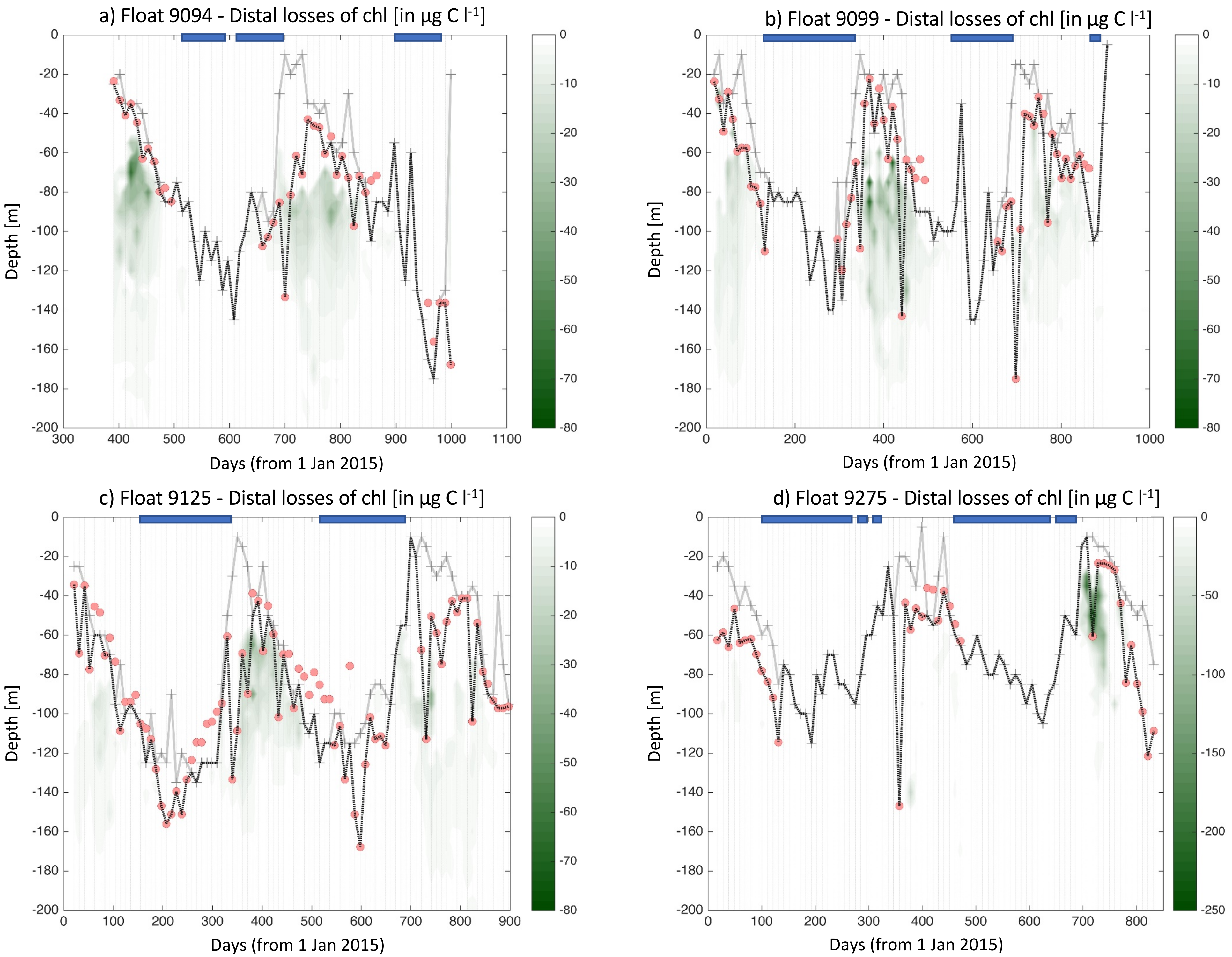

a) Float 9094 - Distal losses of chl [in µg C l-1]
b) Float 9099 - Distal losses of chl [in µg C l-1]
c) Float 9125 - Distal losses of chl [in µg C l-1]
d) Float 9275 - Distal losses of chl [in µg C l-1]
Depth [m]
Days (from 1 Jan 2015)

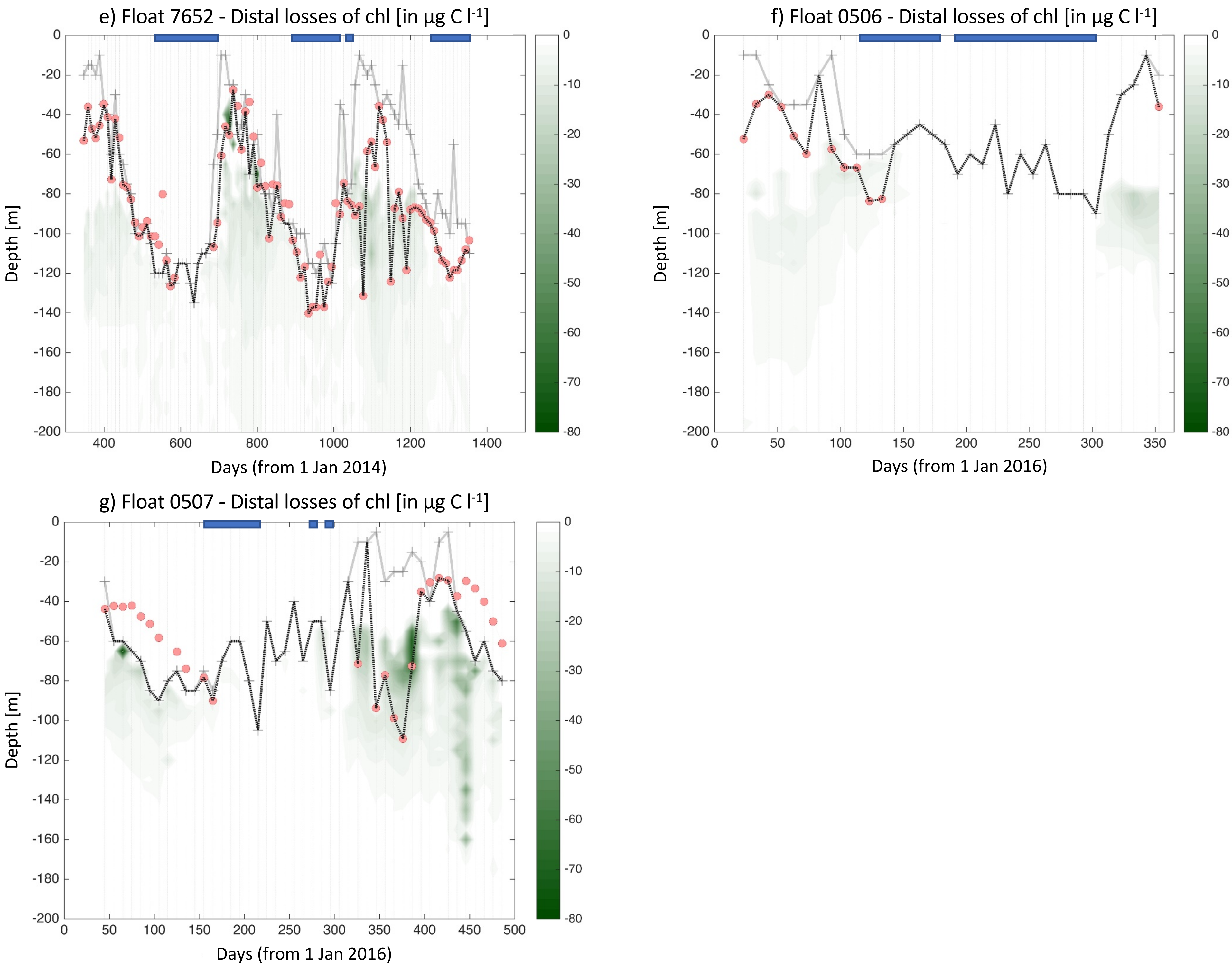
e) Float 7652 - Distal losses of chl [in µg C l-1]
Depth [m]
Days (from 1 Jan 2014)
f) Float 0506 - Distal losses of chl [in µg C l-1]
Depth [m]
Days (from 1 Jan 2016)
g) Float 0507 - Distal losses of chl [in µg C l-1]
Depth [m]
Days (from 1 Jan 2016)

Supplementary Figure 1: Distal losses (in µg C l^{-1}) for floats 9094 (a), 9099 (b), 9125 (c), 9275 (d), 7652 (e), 0506 (f) and 0507 (g). Thick dashed line indicates the deepest of the euphotic zone depth (Zeu, red circles) defined from ref.[12], and the mixed layer depth (grey crosses and grey line) defined from ref.[11], as the depth at which density increased by 0.01 kg m^{-3} compared to density at the surface. Winter mixed layer across all seven floats varied from 90 to 175 m deep, averaging 125 ± 6 m. Thin dashed lines indicate float profiles.

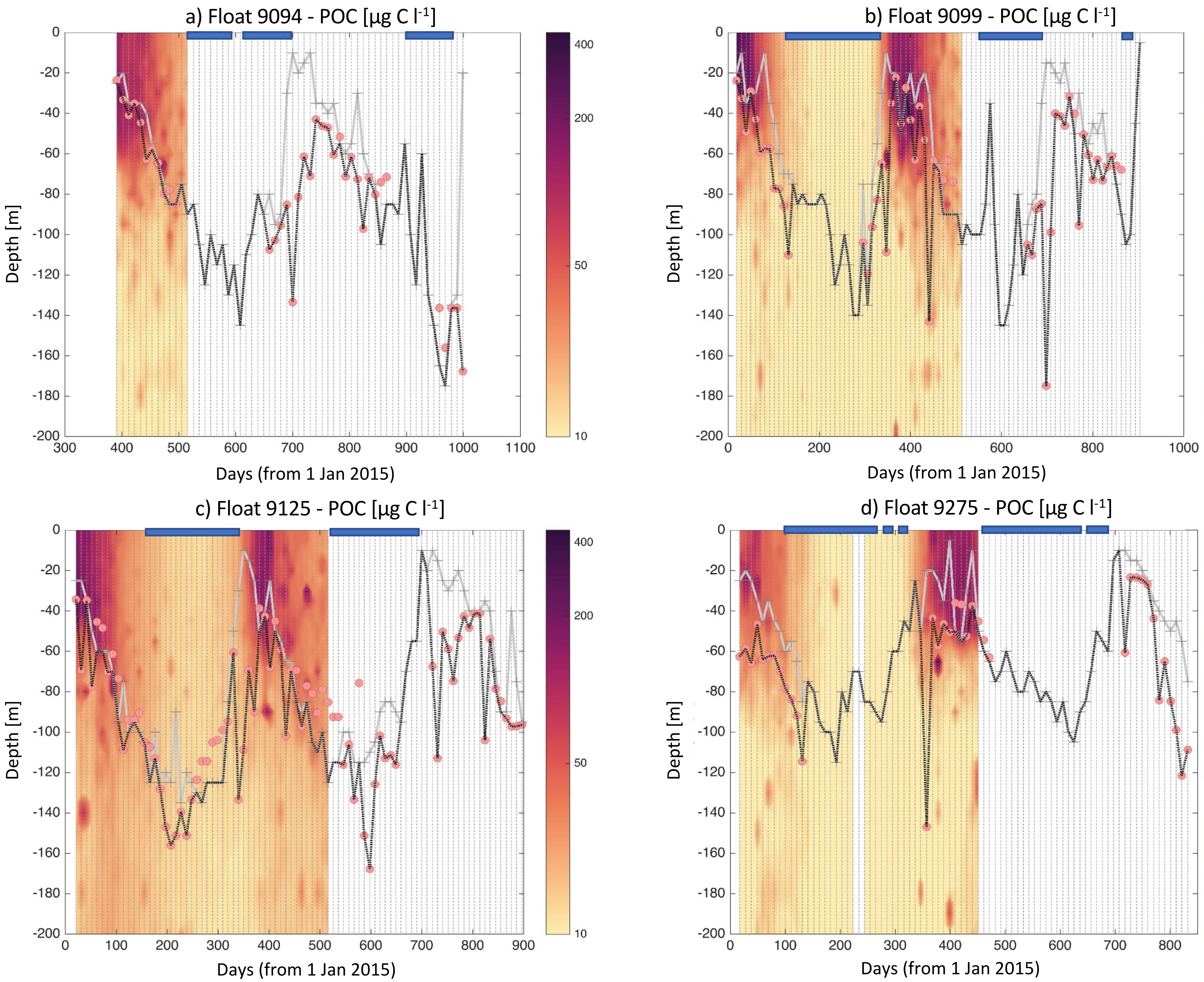

a) Float 9094 - POC [µg C l-1]
b) Float 9099 - POC [µg C l-1]
c) Float 9125 - POC [µg C l-1]
d) Float 9275 - POC [µg C l-1]
Depth [m]
Days (from 1 Jan 2015)
0
-20
-40
-60
-80
-100
-120
-140
-160
-180
-200
10
50
200
400

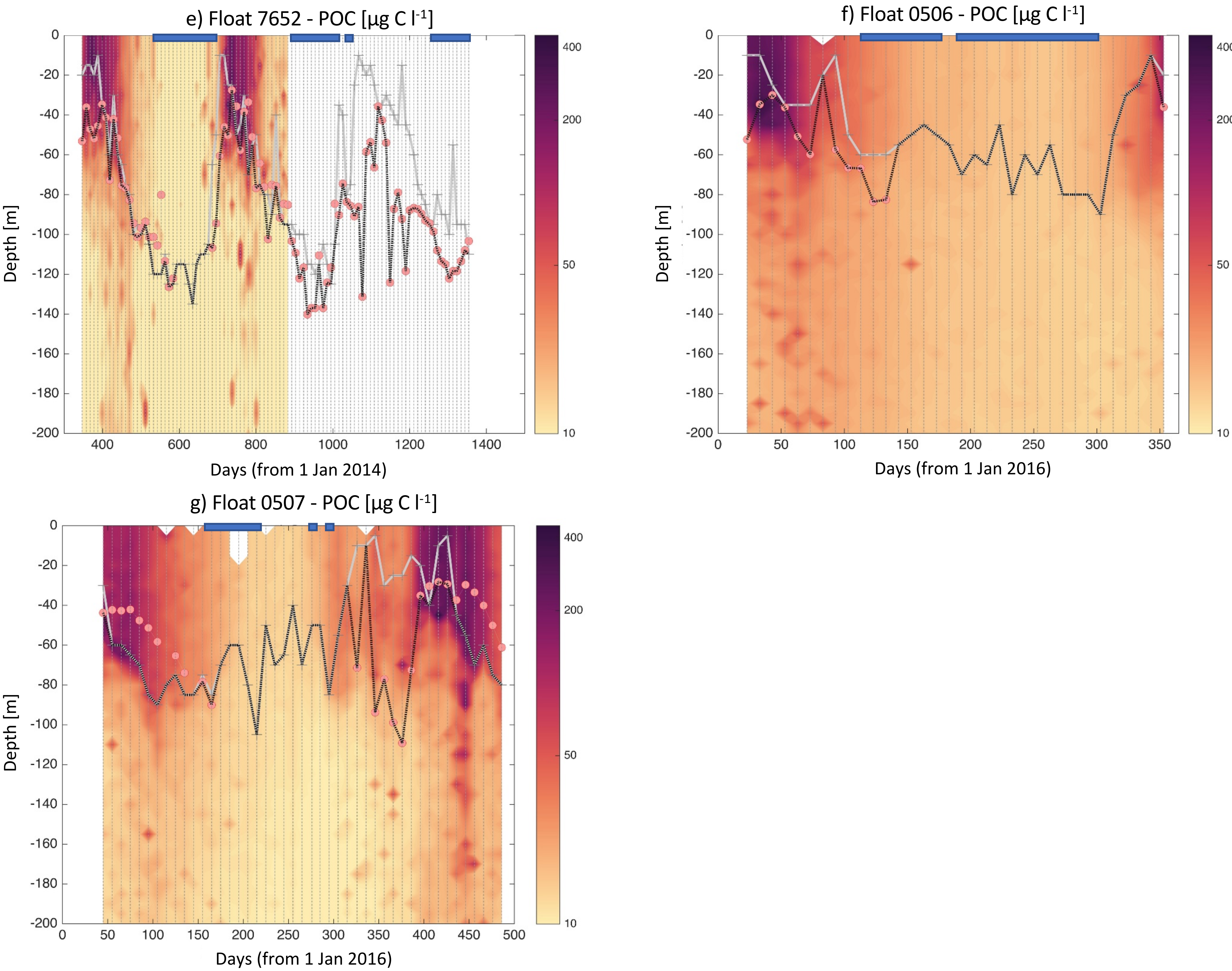

e) Float 7652 - POC [µg C l-1]
Depth [m]
Days (from 1 Jan 2014)
0
-20
-40
-60
-80
-100
-120
-140
-160
-180
-200
400
600
800
1000
1200
1400
400
200
50
10
f) Float 0506 - POC [µg C l-1]
Depth [m]
Days (from 1 Jan 2016)
0
50
100
150
200
250
300
350
g) Float 0507 - POC [µg C l-1]
Depth [m]
Days (from 1 Jan 2016)
0
50
100
150
200
250
300
350
400
450
500

Supplementary Figure 2: Particulate organic carbon (POC, µg C l^{-1}) profiles for floats 9094 (a), 9099 (b), 9125 (c), 9275 (d), 7652 (e), 0506 (f) and 0507 (g). Thick dashed line indicates the deepest of the euphotic zone depth (Zeu, red circles) defined from ref.[12], and the mixed layer depth (grey crosses and grey line) defined from ref.[11], as the depth at which density increased by 0.01 kg m^{-3} compared to density at the surface. Winter mixed layer across all seven floats varied from 90 to 175 m deep, averaging 125 ± 6 m. Thin dashed lines indicate float profiles.

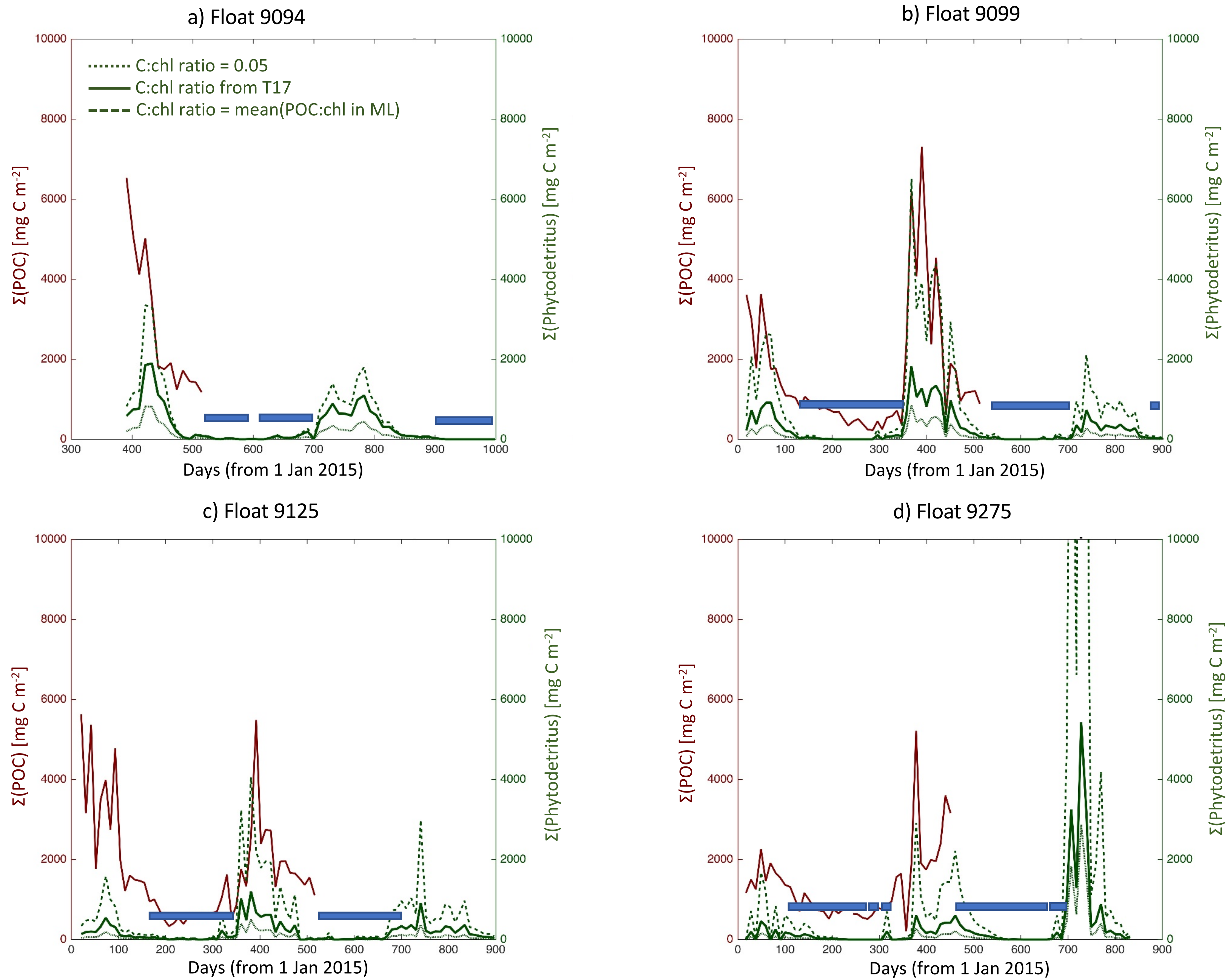

a) Float 9094
b) Float 9099
c) Float 9125
d) Float 9275
C:chl ratio = 0.05
C:chl ratio from T17
C:chl ratio = mean(POC:chl in ML)
Σ(POC) [mg C m-2]
Σ(Phytodetritus) [mg C m-2]
Days (from 1 Jan 2015)

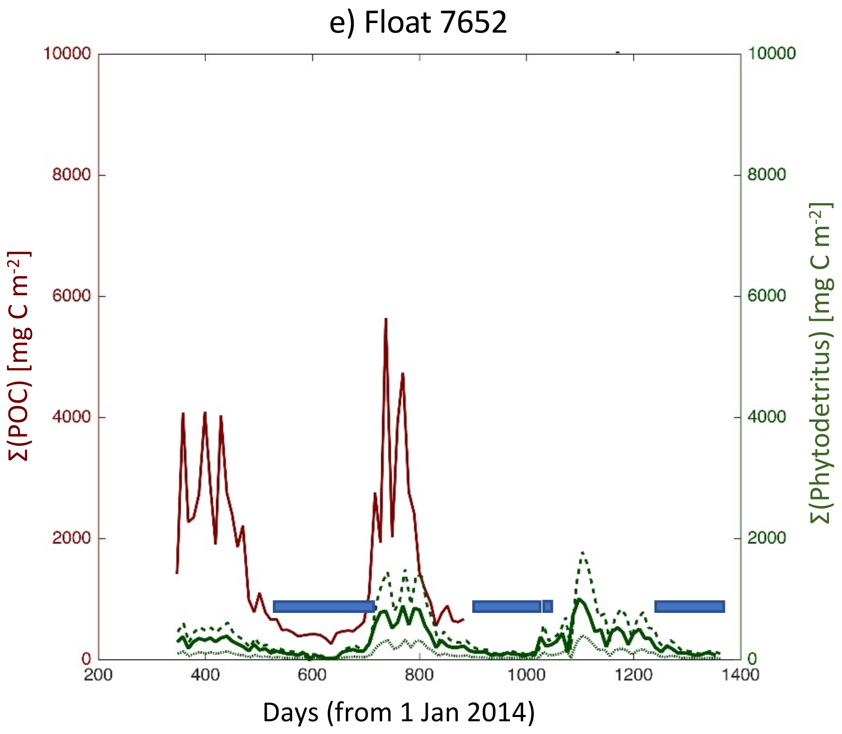
e) Float 7652
Σ(POC) [mg C m-2]
Σ(Phytodetritus) [mg C m-2]
Days (from 1 Jan 2014)

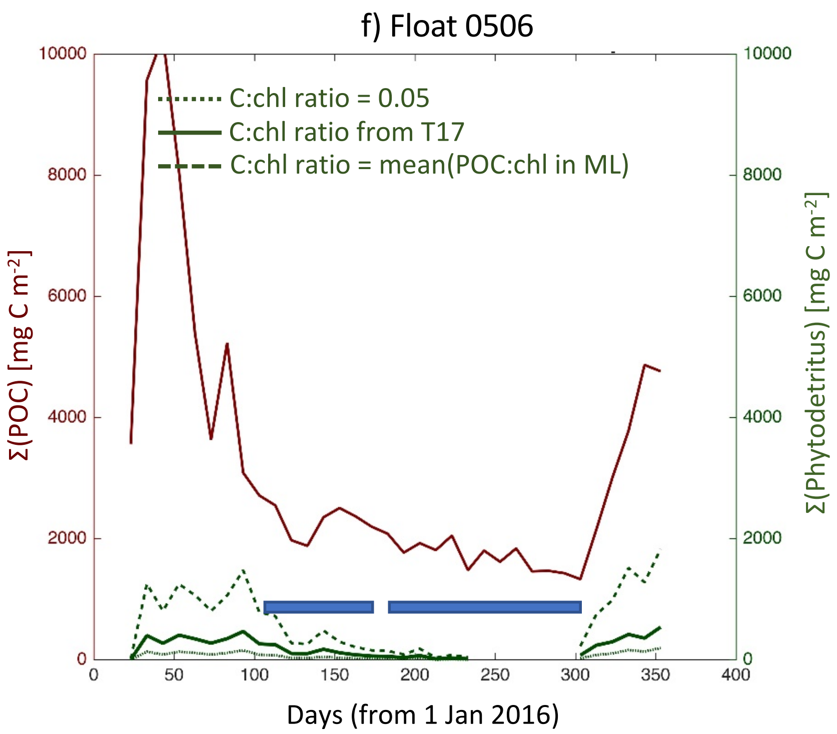
f) Float 0506
C:chl ratio = 0.05
C:chl ratio from T17
C:chl ratio = mean(POC:chl in ML)
Σ(POC) [mg C m-2]
Σ(Phytodetritus) [mg C m-2]
Days (from 1 Jan 2016)

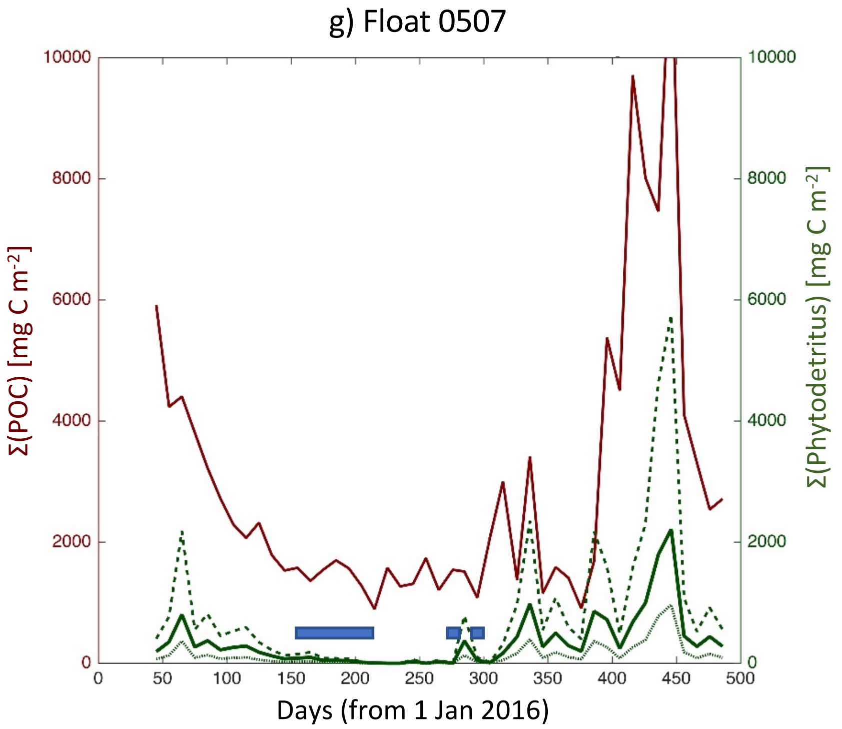
g) Float 0507
Σ(POC) [mg C m-2]
Σ(Phytodetritus) [mg C m-2]
Days (from 1 Jan 2016)

Supplementary Figure 3: Exported POC and phytodetritus (mg C m^{-2}) derived from several C:chl ratios (0.05 µg C/µg chl, T17 and the mean POC:chl ratio in the ML) and integrated from the export depth to 175 m, for floats 9094 (a), 9099 (b), 9125 (c), 9275 (d), 7652 (e), 0506 (f) and 0507 (g).

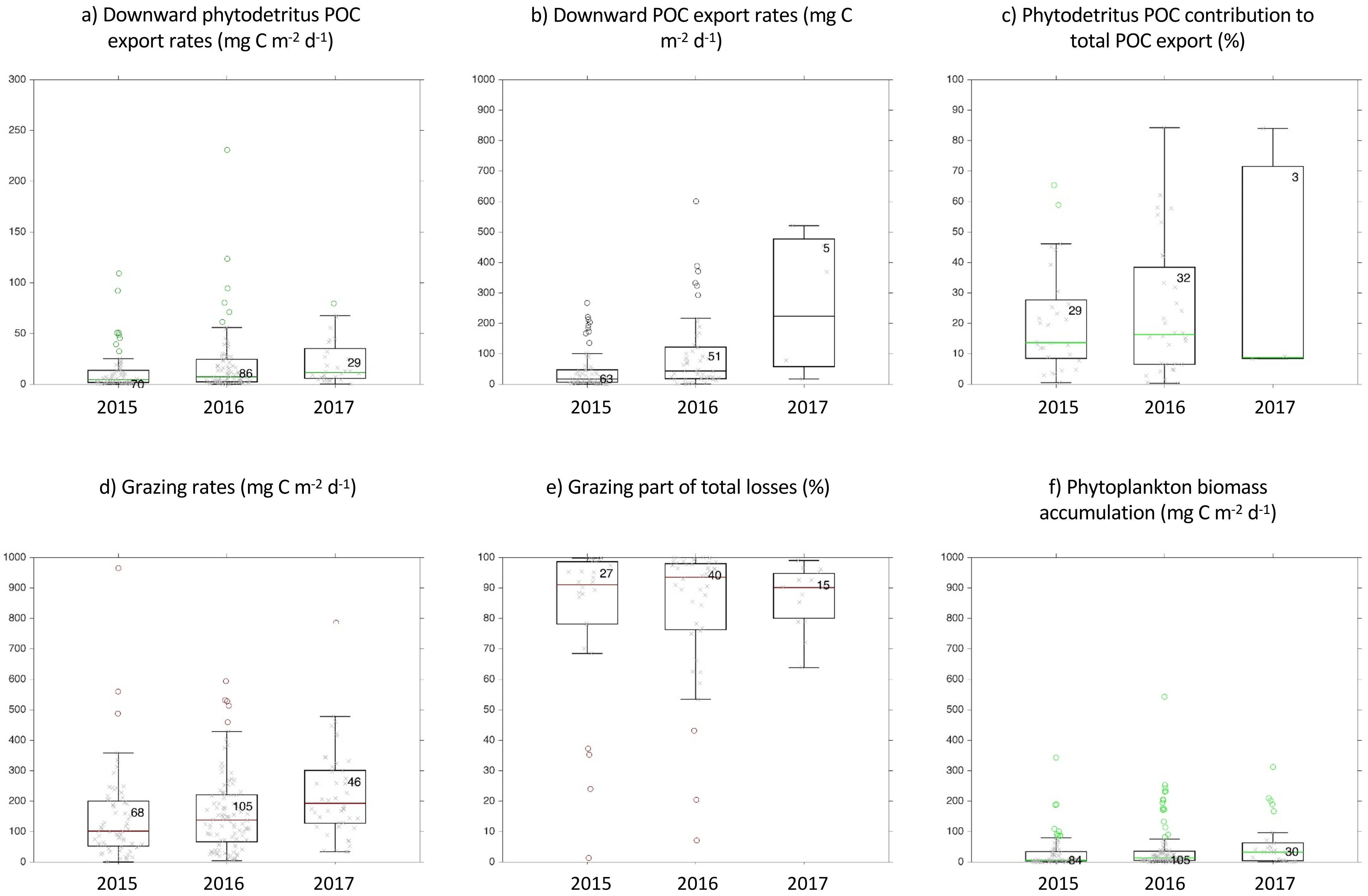

a) Downward phytodetritus POC export rates (mg C m-2 d-1)
0
50
100
150
200
250
300
70
86
29
2015
2016
2017
b) Downward POC export rates (mg C m-2 d-1)
0
100
200
300
400
500
600
700
800
900
1000
63
51
5
2015
2016
2017
c) Phytodetritus POC contribution to total POC export (%)
0
10
20
30
40
50
60
70
80
90
100
29
32
3
2015
2016
2017
d) Grazing rates (mg C m-2 d-1)
0
100
200
300
400
500
600
700
800
900
1000
68
105
46
2015
2016
2017
e) Grazing part of total losses (%)
0
10
20
30
40
50
60
70
80
90
100
27
40
15
2015
2016
2017
f) Phytoplankton biomass accumulation (mg C m-2 d-1)
0
100
200
300
400
500
600
700
800
900
1000
84
105
30
2015
2016
2017

Supplementary Figure 4: Annual distribution of phytoplankton fates. Box-plot of integrated a) phytodetritus POC export (mg C m^{-2} d^{-1}), b) POC export (mg C m^{-2} d^{-1}), c) phytodetritus POC contribution to total POC export (%), d) grazing (mg C m^{-2} d^{-1}), e) grazing part of total losses (%) and f) phytoplankton biomass accumulation (mg C m^{-2} d^{-1}). The boxplot shows the median, the 50% central region, and the functional limits ([Q1-1.5*IQR, Q3+1.5*IQR], where Q1 and Q3 are the 25th and 75th percentiles respectively, and IQR is the interquartile range). Data are plotted as crosses and outliers as circles. The sample size is indicated for each box.

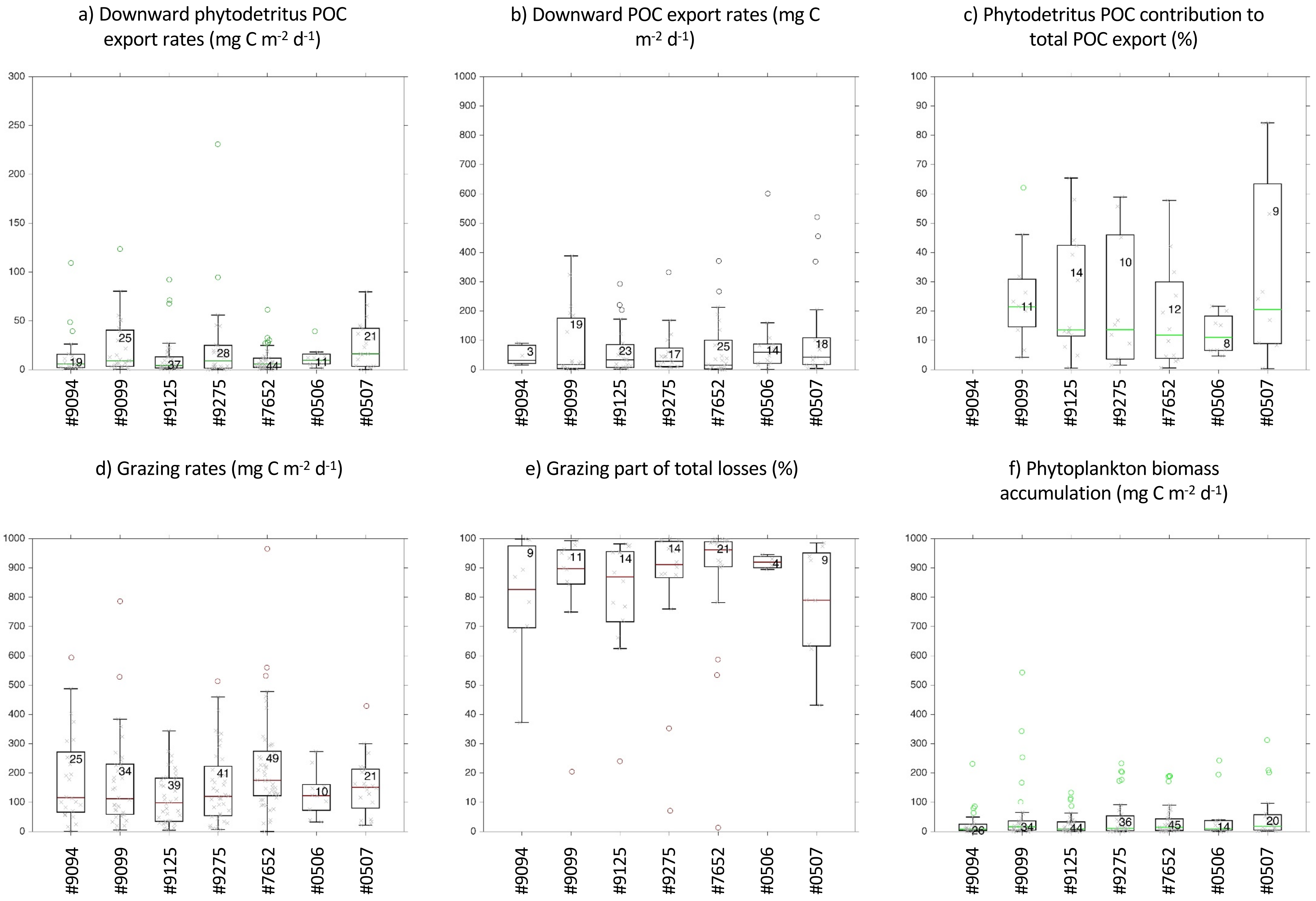

a) Downward phytodetritus POC export rates (mg C m-2 d-1)
b) Downward POC export rates (mg C m-2 d-1)
c) Phytodetritus POC contribution to total POC export (%)
d) Grazing rates (mg C m-2 d-1)
e) Grazing part of total losses (%)
f) Phytoplankton biomass accumulation (mg C m-2 d-1)
#9094
#9099
#9125
#9275
#7652
#0506
#0507

Supplementary Figure 5: Floats distribution of phytoplankton fates. Box-plot of integrated a) phytodetritus POC export (mg C m^{-2} d^{-1}), b) POC export (mg C m^{-2} d^{-1}), c) phytodetritus POC contribution to total POC export (%), d) grazing (mg C m^{-2} d^{-1}), e) grazing part of total losses (%) and f) phytoplankton biomass accumulation (mg C m^{-2} d^{-1}). The boxplot shows the median, the 50% central region, and the functional limits ([Q1-1.5*IQR, Q3+1.5*IQR], where Q1 and Q3 are the 25th and 75th percentiles respectively, and IQR is the interquartile range). Data are plotted as crosses and outliers as circles. The sample size is indicated for each box.

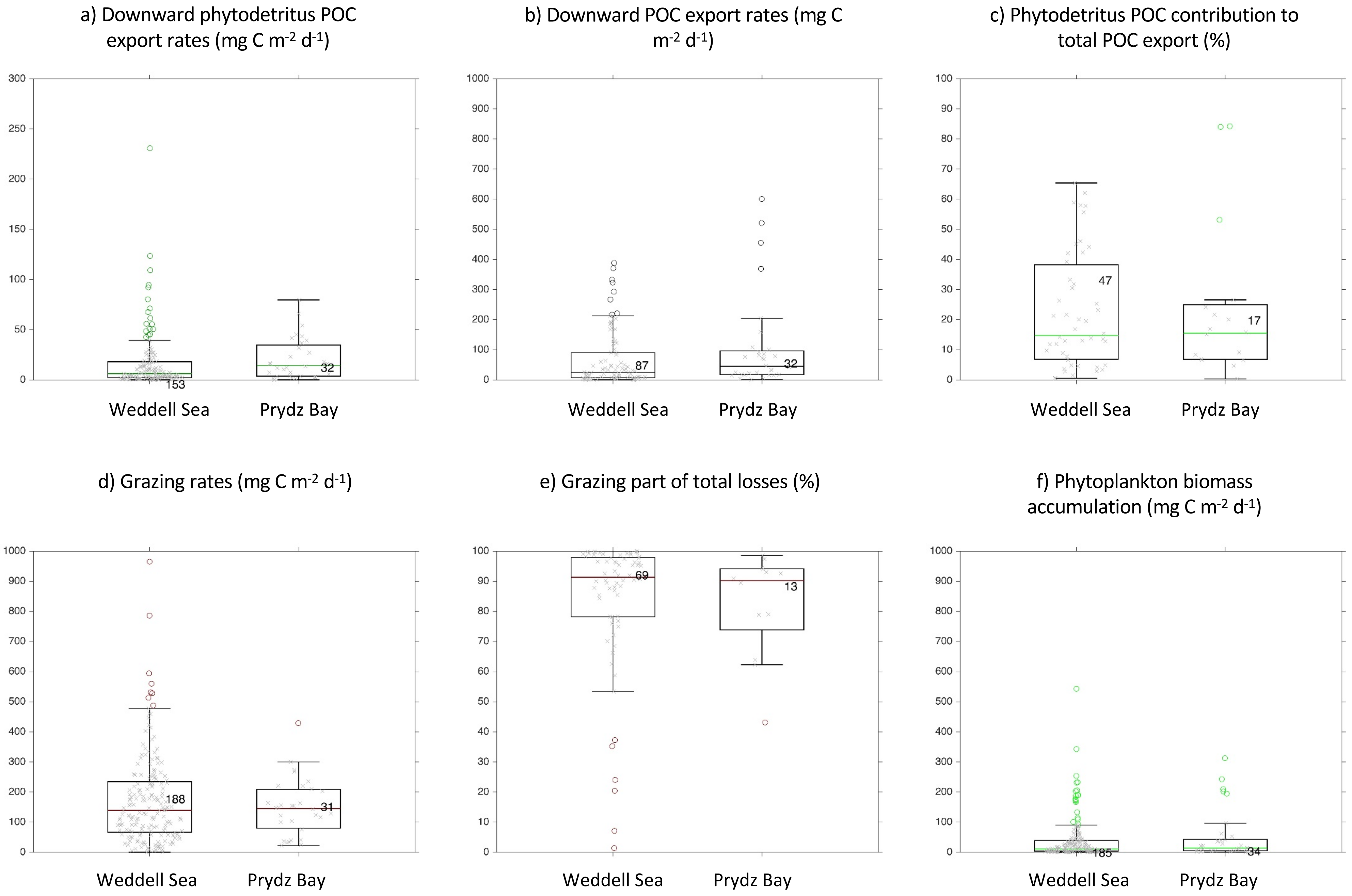

a) Downward phytodetritus POC export rates (mg C m-2 d-1)
153
32
Weddell Sea
Prydz Bay
b) Downward POC export rates (mg C m-2 d-1)
87
32
Weddell Sea
Prydz Bay
c) Phytodetritus POC contribution to total POC export (%)
47
17
Weddell Sea
Prydz Bay
d) Grazing rates (mg C m-2 d-1)
188
31
Weddell Sea
Prydz Bay
e) Grazing part of total losses (%)
69
13
Weddell Sea
Prydz Bay
f) Phytoplankton biomass accumulation (mg C m-2 d-1)
185
34
Weddell Sea
Prydz Bay

Supplementary Figure 6: Area distribution of phytoplankton fates. Box-plot of integrated a) phytodetritus POC export (mg C m^{-2} d^{-1}), b) POC export (mg C m^{-2} d^{-1}), c) phytodetritus POC contribution to total POC export (%), d) grazing (mg C m^{-2} d^{-1}), e) grazing part of total losses (%) and f) phytoplankton biomass accumulation (mg C m^{-2} d^{-1}). The boxplot shows the median, the 50% central region, and the functional limits ([Q1-1.5*IQR, Q3+1.5*IQR], where Q1 and Q3 are the 25th and 75th percentiles respectively, and IQR is the interquartile range). Data are plotted as crosses and outliers as circles. The sample size is indicated for each box.

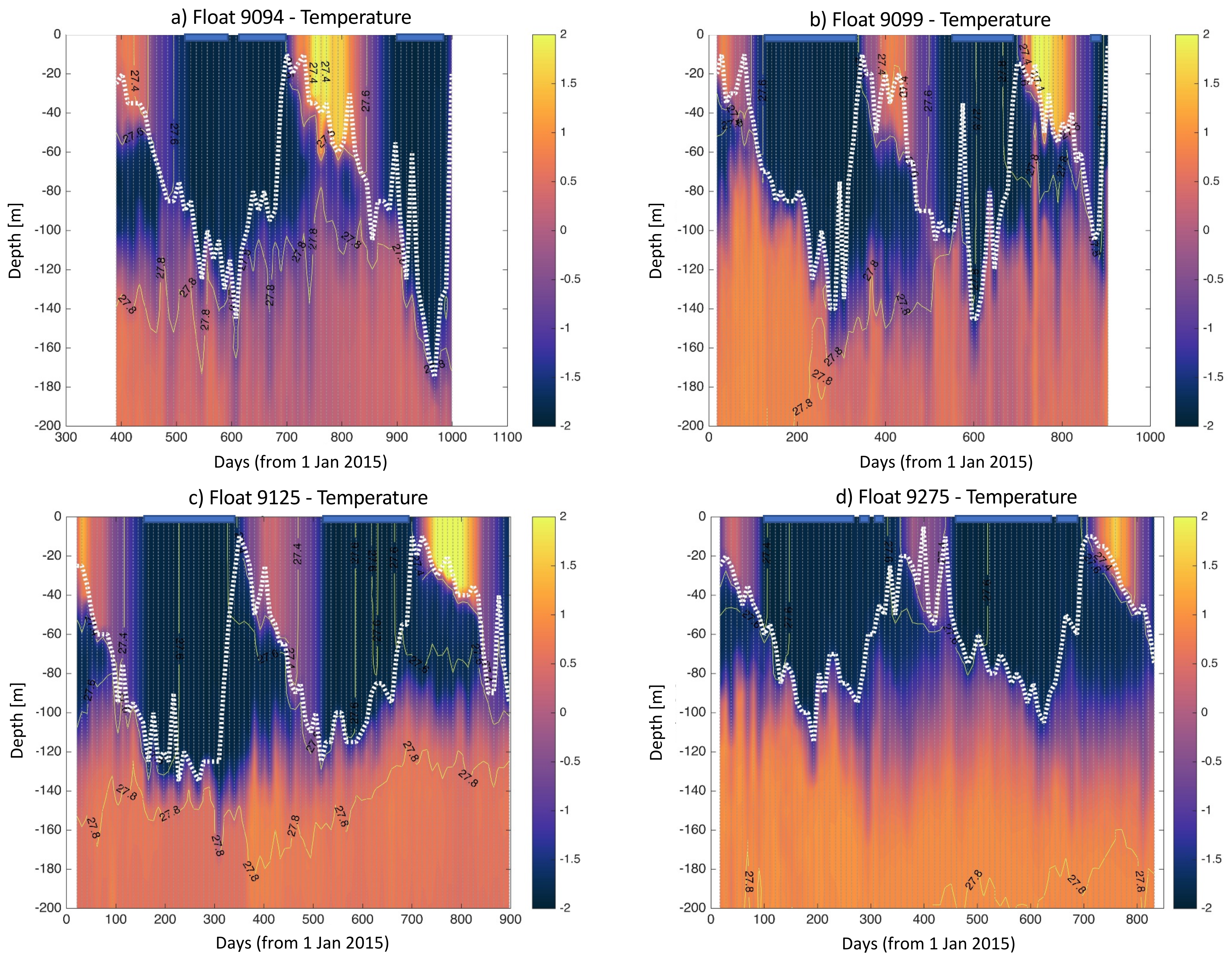
a) Float 9094 - Temperature
b) Float 9099 - Temperature
c) Float 9125 - Temperature
d) Float 9275 - Temperature
Depth [m]
Days (from 1 Jan 2015)
27.4
27.6
27.8

e) Float 7652 - Temperature

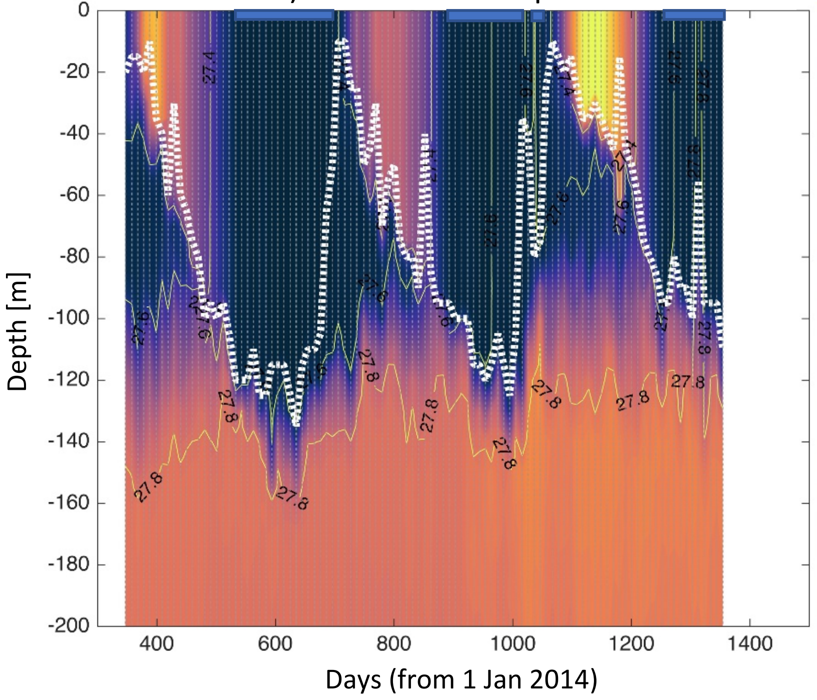

f) Float 0506 - Temperature

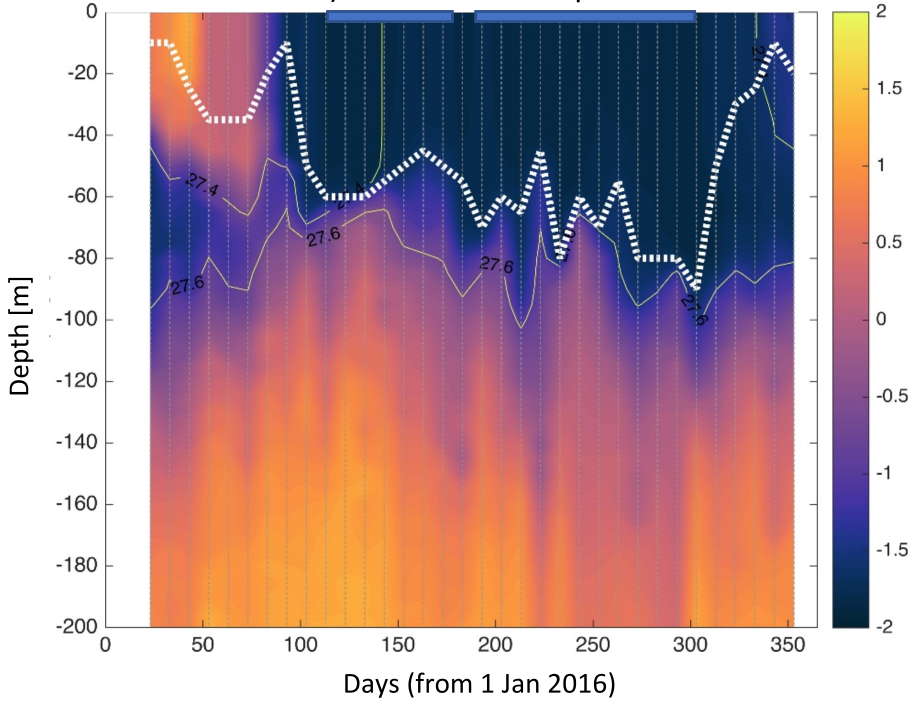

g) Float 0507 - Temperature

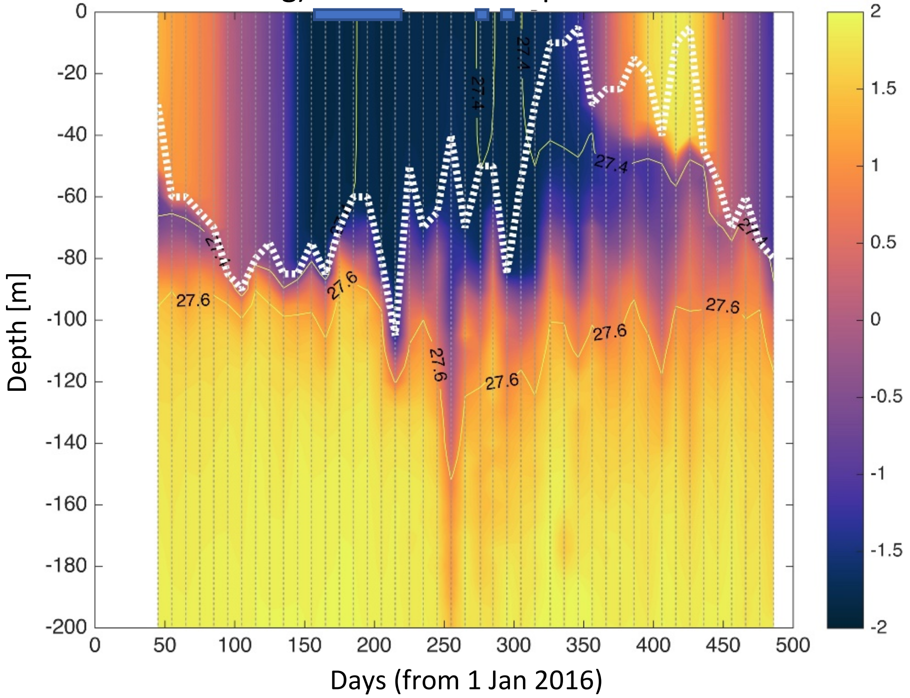

Supplementary Figure 7: Temperature (C) for floats 9094 (a), 9099 (b), 9125 (c), 9275 (d), 7652 (e), 0506 (f) and 0507 (g). Potential density isopycnals are contoured. Thick dashed lines indicate mixed layer depth defined from ref.[11] as the depth at which density increased by 0.01 kg m^{-3} compared to density at the surface. Winter mixed layer across all seven floats varied from 90 to 175 m deep, averaging 125 ± 6 m. Thin dashed lines indicate float profiles.

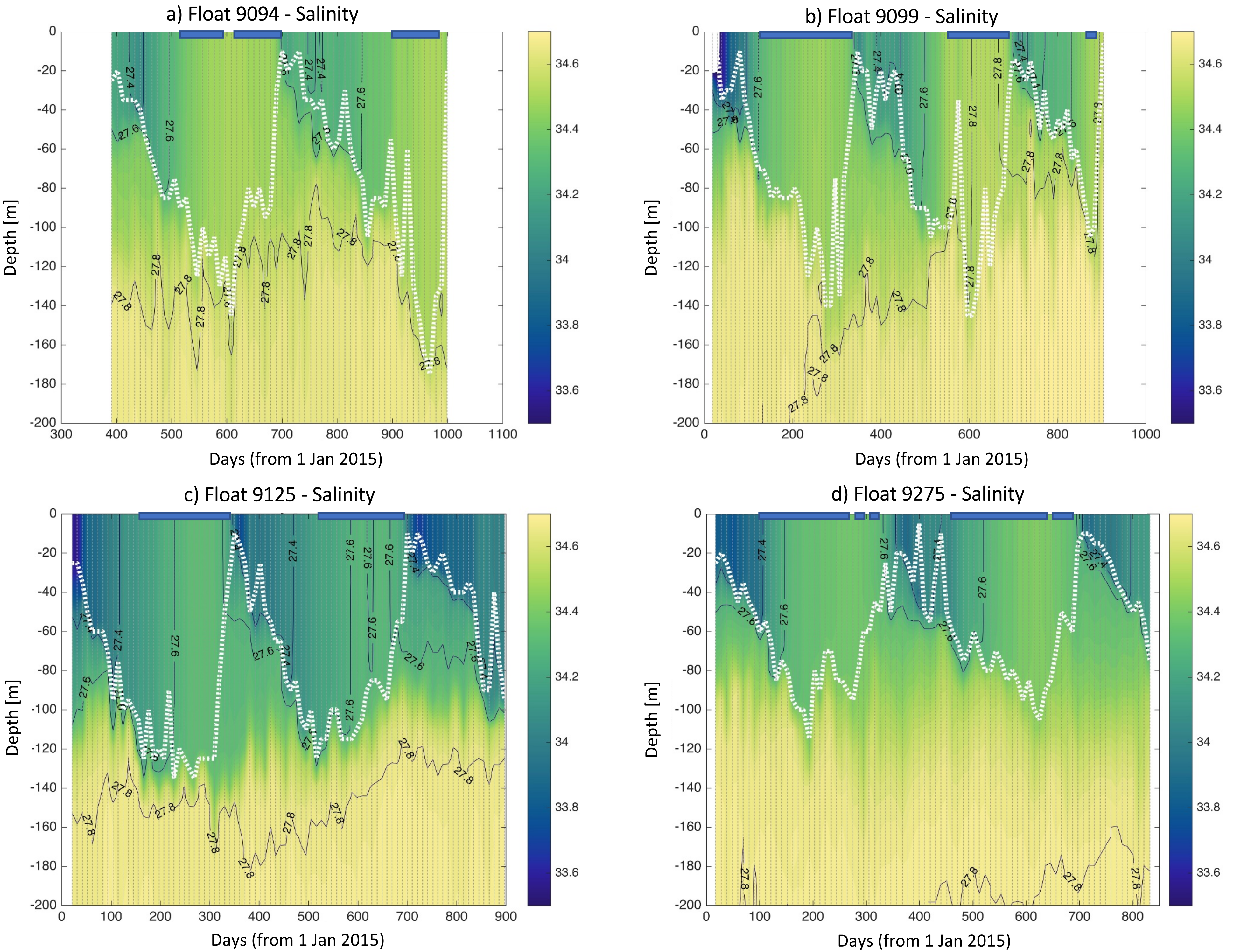
a) Float 9094 - Salinity
b) Float 9099 - Salinity
c) Float 9125 - Salinity
d) Float 9275 - Salinity
Depth [m]
Days (from 1 Jan 2015)
27.4
27.6
27.8
34.6
34.4
34.2
34
33.8
33.6

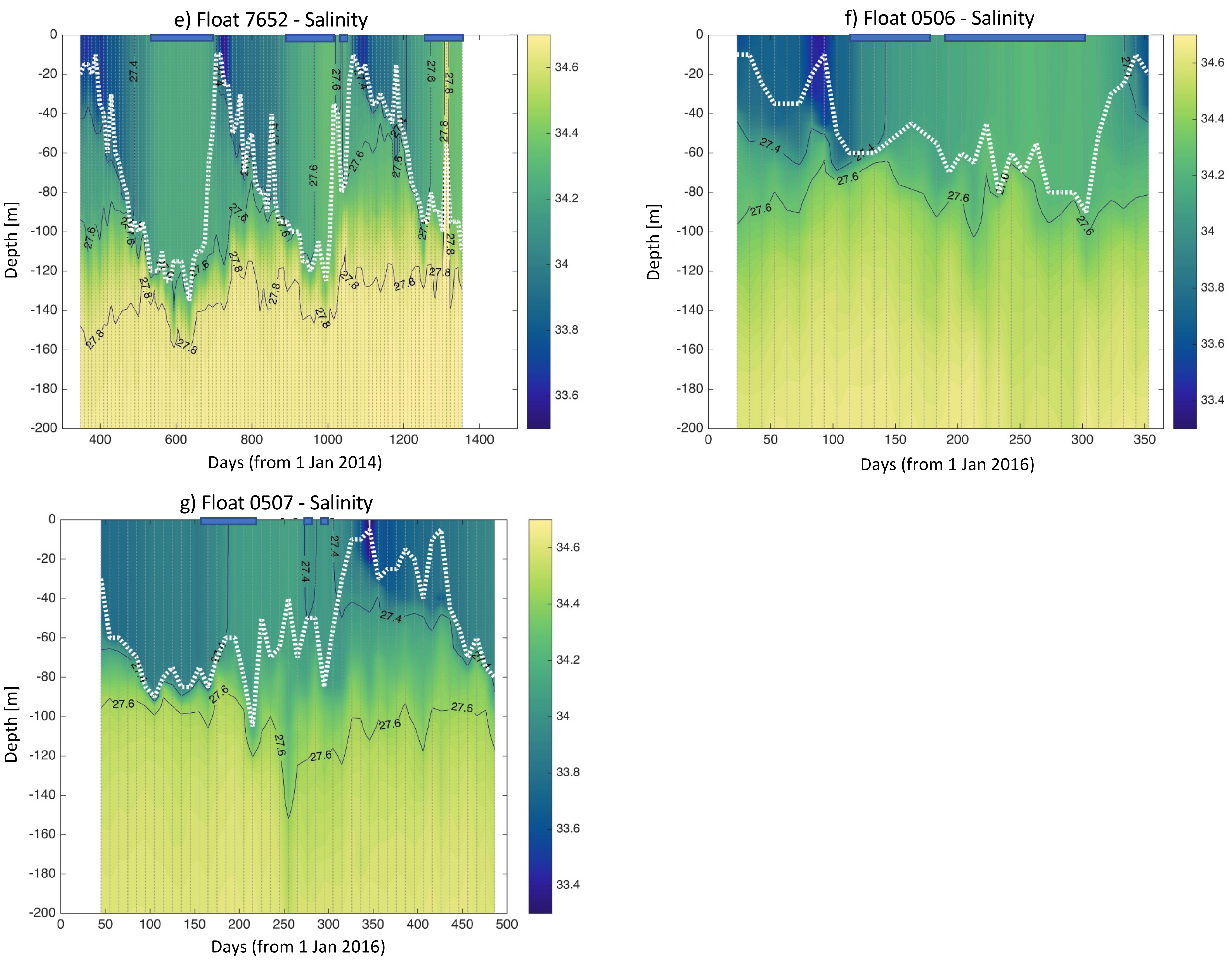
e) Float 7652 - Salinity
Depth [m]
Days (from 1 Jan 2014)
27.4
27.6
27.8
34.6
34.4
34.2
34
33.8
33.6
f) Float 0506 - Salinity
Depth [m]
Days (from 1 Jan 2016)
27.4
27.6
34.6
34.4
34.2
34
33.8
33.6
33.4
g) Float 0507 - Salinity
Depth [m]
Days (from 1 Jan 2016)
27.4
27.6
34.6
34.4
34.2
34
33.8
33.6
33.4

Supplementary Figure 8: Salinity (PSU) for floats 9094 (a), 9099 (b), 9125 (c), 9275 (d), 7652 (e), 0506 (f) and 0507 (g). Potential density isopycnals are contoured. Thick dashed lines indicate mixed layer depth defined from ref.[11] as the depth at which density increased by 0.01 kg m^{-3} compared to density at the surface. Winter mixed layer across all seven floats varied from 90 to 175 m deep, averaging 125 ± 6 m. Thin dashed lines indicate float profiles.

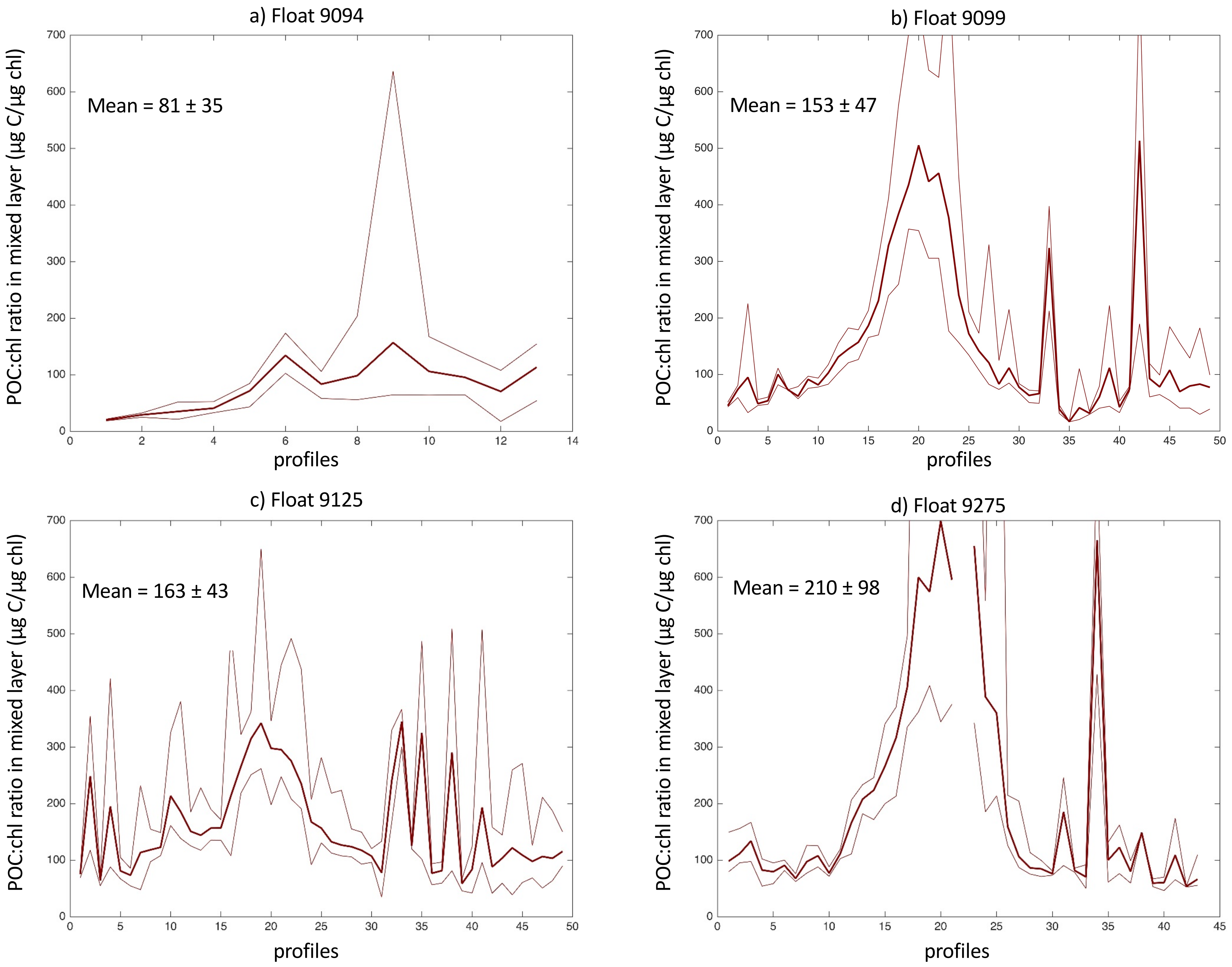

a) Float 9094
Mean = 81 ± 35
b) Float 9099
Mean = 153 ± 47
c) Float 9125
Mean = 163 ± 43
d) Float 9275
Mean = 210 ± 98
POC:chl ratio in mixed layer (µg C/µg chl)
profiles

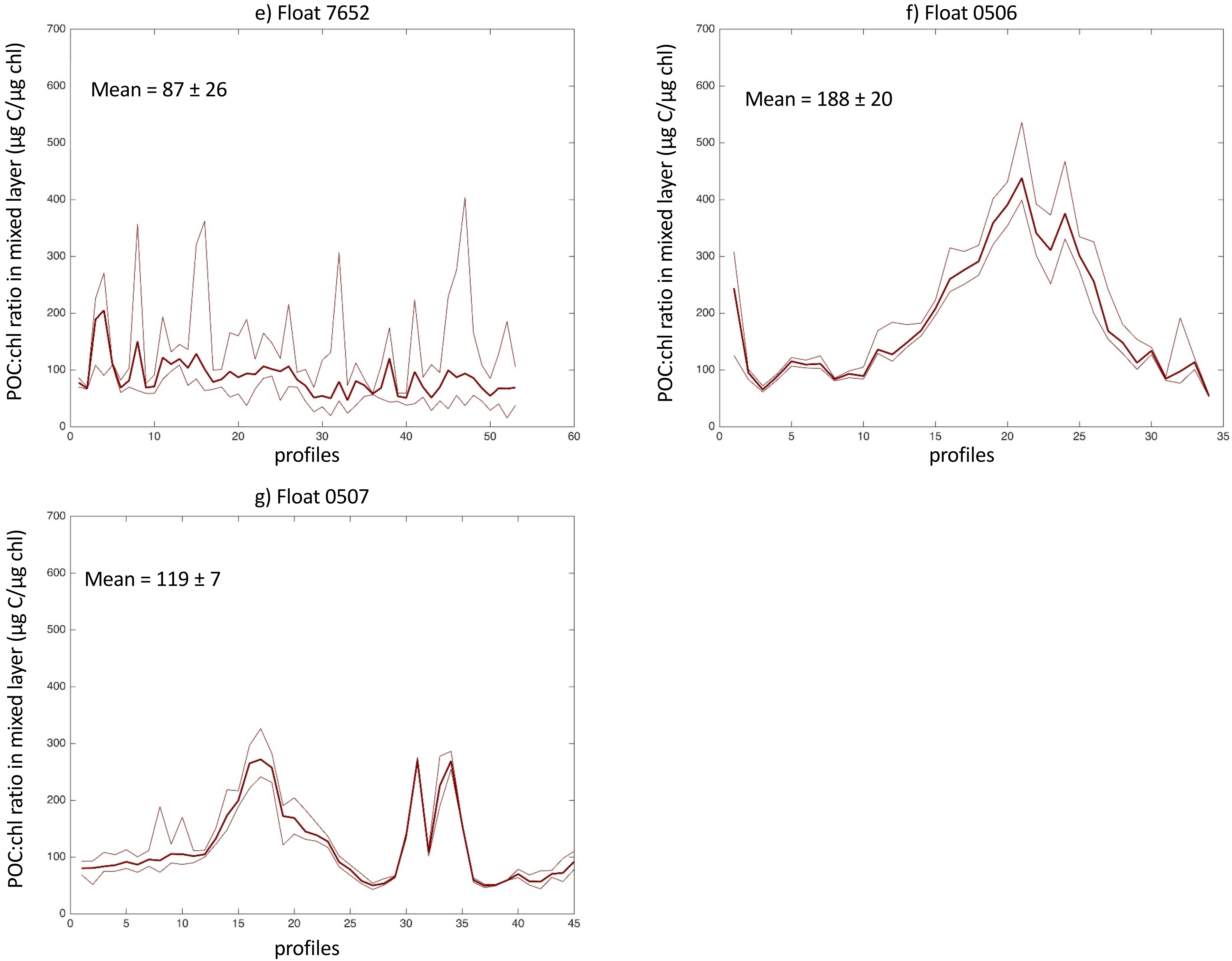

e) Float 7652
Mean = 87 ± 26
POC:chl ratio in mixed layer (µg C/µg chl)
profiles
f) Float 0506
Mean = 188 ± 20
POC:chl ratio in mixed layer (µg C/µg chl)
profiles
g) Float 0507
Mean = 119 ± 7
POC:chl ratio in mixed layer (µg C/µg chl)
profiles

Supplementary Figure 9: Average (thick line), minimum (lower thin line) and maximum (upper thin line) POC:chl ratio (µg C/µg chl) in the surface mixed layer for floats 9094 (a), 9099 (b), 9125 (c), 9275 (d), 7652 (e), 0506 (f) and 0507 (g).

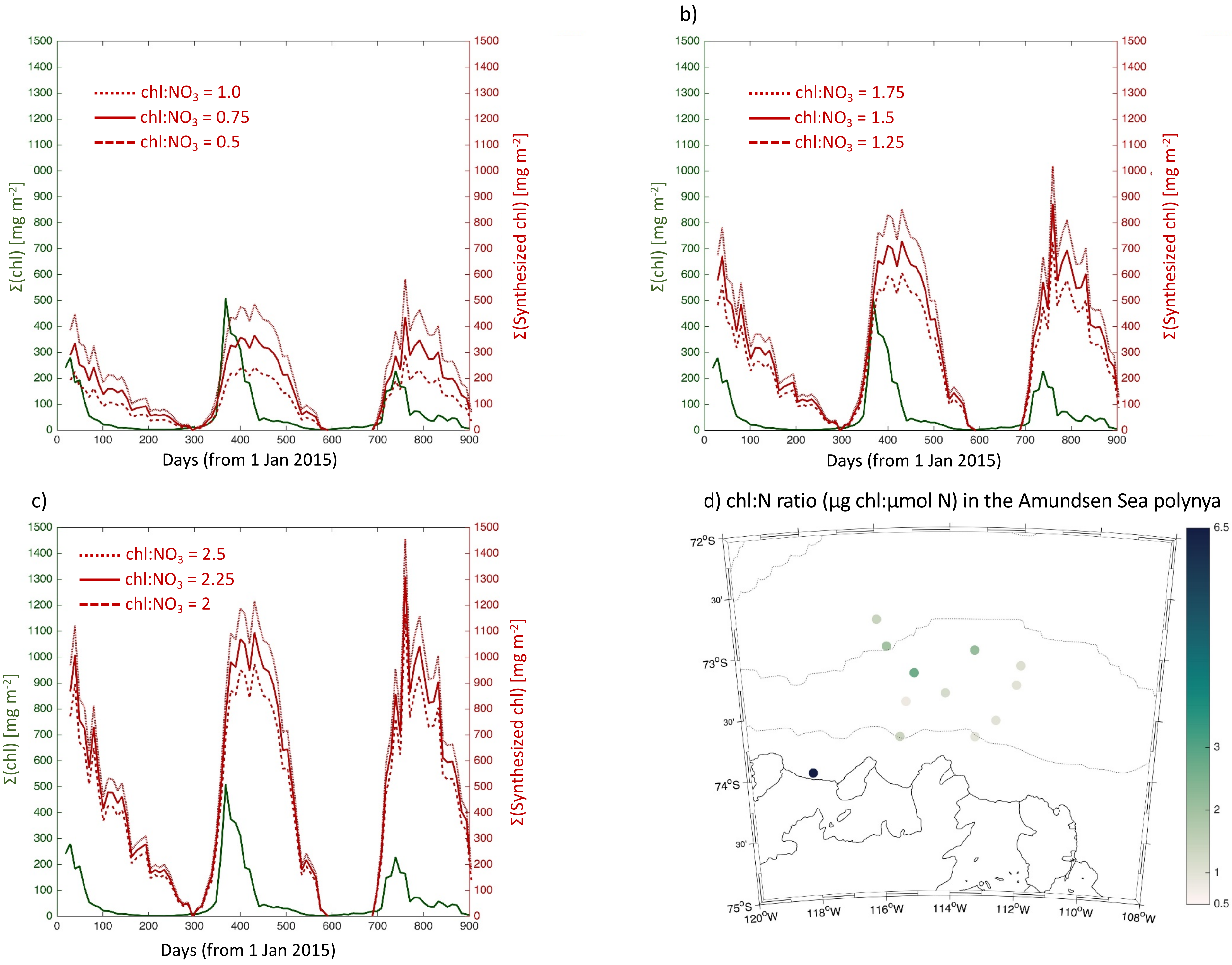

chl:NO3 = 1.0
chl:NO3 = 0.75
chl:NO3 = 0.5
Σ(chl) [mg m-2]
Σ(Synthesized chl) [mg m-2]
Days (from 1 Jan 2015)
b)
chl:NO3 = 1.75
chl:NO3 = 1.5
chl:NO3 = 1.25
Σ(chl) [mg m-2]
Σ(Synthesized chl) [mg m-2]
Days (from 1 Jan 2015)
c)
chl:NO3 = 2.5
chl:NO3 = 2.25
chl:NO3 = 2
Σ(chl) [mg m-2]
Σ(Synthesized chl) [mg m-2]
Days (from 1 Jan 2015)
d) chl:N ratio (µg chl:µmol N) in the Amundsen Sea polynya
72°S
30'
73°S
30'
74°S
30'
75°S
120°W
118°W
116°W
114°W
112°W
110°W
108°W
6.5
3
2
1
0.5

Supplementary Figure 10: Sensitivity of the method to the chl:N ratio. Observed chl (mg m^{-2}) and theoretical maximum chl (mg m^{-2}) integrated over the upper 100 m of the water column for float #9099, and shown for several chl:N ratios (units of µg chl:µmol N): a) 0.5, 0.75 and 1 µg chl:µmol N; b) 1.25, 1.5 and 1.75 µg chl:µmol N; and c) 2, 2.25 and 2.5 µg chl:µmol N. d) Map of the chl:N ratio (µg chl:µmol N) measured in the Amundsen Sea polynya[31].
