# Supplementary material for: Remote assessment of the fate of phytoplankton in the Southern Ocean sea-ice zone
Source: Nat Commun. 2020 Jun 19;11:3108. doi: 10.1038/s41467-020-16931-0 (PMC7305117; doi:10.1038/s41467-020-16931-0)
Supplement: Supplementary file 2 — Peer Review File [file 41467_2020_16931_MOESM2_ESM.docx]

Peer Review Information

**Journal:** Nature Communications

**Manuscript title:** Remote assessment of the fate of phytoplankton in the Southern Ocean sea ice zone

**Corresponding author name(s):** Dr Sebastien Moreau

**Editorial notes:**

**Transferred manuscripts**

This manuscript has been previously reviewed at another journal that is not operating a transparent peer review scheme. This document only contains reviewer comments and rebuttal letters for versions considered at *Nature Communications*.

**Reviewer comments & decisions:**

**Reviewer comments, first version:**

Reviewer #1 (Remarks to the Author):

This paper uses biogeochemical ARGO floats to understand the fate of phytoplankton in the Seasonal Ice Zone of the Southern Ocean. The study is novel in that it attempts to use the data to evaluate not just the rates, but the fates of productivity by uncoupling grazing and sinking rates. Of note, the paper estimates that grazing accounts for 90% of the average losses of productivity. This is a novel and interesting finding. I enjoyed reading this paper very much and appreciated the unique methods to calculate loss terms. Obviously, their estimates are based on some assumption, such as a constant chl:NO3 ratio, that can be quite variable in space and time. However, I think they do a reasonable job of validating their estimates with other data sources and considering the variability between floats over space and over time throughout the growing season. I think this pushes the ball forward in terms of what can be done with float data and definitely contributes to an understanding of a complex ecosystem. It opens the door to new questions and types of data that is needed to further refine these estimates. I think this novel study deserves to be published. My main comments are largely stylistic in nature in terms of the methods, figures and the representation of the results in figures and a bit more validation.

Regarding the figures, I think they have some of the most compelling figures tucked away in the supplemental section. I have not seen many estimate of de novo photosynthesis like Supp. Fig. A1 which shows the synthesized chlorophyll in relationship to the measured under different nitrate assumptions – this is a powerful validation of their approach. The mechanistic Supp. Fig. A2 is also quite useful in discerning the approach and conceptual framework for the study. I also thought Fig. A7 was useful in discerning where the floats are located in relationship to the entirety of the SIZ Southern Ocean. I think rearrangement of some of these figures into composites in the main article and reduction and summaries of the some of the bar graphs would be useful in the main text.

They note that this is the first basin scale, observational estimate of grazing throughout the SIZ of the Southern Ocean, but Fig 3 in the main paper is kind of disappointing with blobs that are hard to tell superimposed on chlorophyll of similar colors and does not really provide a corresponding synthesis of grazing across the SIZ. How representative is 158 average number across the whole SIZ? Where is the SIZ in the figure?

The methods are a bit confusing to figure out in supplemental materials, but I gather in the nonphotochemical quenching section that daytime fluorescence was used to estimate chlorophyll. How was this done exactly? Did they use the methods of (Haëntjens et al. 2017) and how does the corresponding uncertainty factor into the values?

They also mention that backscattering sensors stopped working (Suppl.) , but looks like there is quite a bit of data shown in Fig. 1. And, the patterns of deeper export particles from bbp look quite intriguing and different as compared to fluorescence. This is mentioned in the text (p. 4 lines 18-19). However, the next sentence say only chlorophyll is used for export. Seems like this would strengthen the export part of the paper and maybe lead to higher export values than estimate with fluorescence.

Also it is a bit unclear as to what depth “export” is defined. Some recent work suggests the maximum winter mixed layer depth is really what constrains export rather than an arbitrary depth horizon (Palevsky and Doney 2018). Is 100 m or 400 m used here? How does the depth horizon chosen influence export results?

Need to label the colorbars in supplemental figures and would be helpful to include figure numbers and legends with the figures.

References
Haëntjens, N., E. Boss, and L. D. Talley. 2017. Revisiting Ocean Color algorithms for chlorophyll a and particulate organic carbon in the Southern Ocean using biogeochemical floats. Journal of Geophysical Research: Oceans 122: 6583–6593.
Palevsky, H. I., and S. C. Doney. 2018. How choice of depth horizon influences the estimated spatial patterns and global magnitude of ocean carbon export flux. Geophysical Research Letters 45: 4171–4179.


Reviewer #2 (Remarks to the Author):

The paper by Moreau and colleagues uses data from BGC-Argo floats to develop a carbon budget for the Southern Ocean, and from that, calculates grazing-mediated losses. The calculation assumes that absent any losses, chlorophyll increases should balance nitrate deficits. If they don’t, then this is due to losses by the sum of export and grazing. Export losses were calculated from depth profiles of chlorophyll and so the residual loss is attributed to grazing. Although mass balance approaches to calculating fluxes do not work everywhere, I believe that their use is appropriate when sampling is on a Lagrangian framework, as it generally is with BGC-Argo floats. I think it will be very useful for elucidating a number of different biogeochemical processes as our understanding of these processes increases.

Having said that, one must be careful that the assumptions going into the calculations are correct. In the present paper, the assumption is that the Chl:N ratio for phytoplankton is 1.75 µg Chl:µmol N, and that this represents a value for chlorophyll synthesis from assimilated nitrogen without any losses. This ratio was based on measurements made during a rapidly developing bloom in the Amundsen Sea, where the authors appear to assume that no losses of phytoplankton were occurring. If correct, this assumption makes it easier to partition grazing losses from export losses based on the balance between Chl accumulation and N depletion. However, there is never a situation where there is no loss of phytoplankton, even during the exponential growth phase of the bloom. There are always some zooplankton present and feeding, even if the growth of phytoplankton far outstrips the losses to grazing. Losses can be surprisingly high at this time, but with such high phytoplankton growth rates, it is difficult to discern. So this suggests that their estimate of grazing loss may be underestimated. The authors need to come up with a better reference value for the amount of chlorophyll synthesis expected from assimilated nitrogen. Perhaps a culture study (preferably axenic) would provide a better benchmark.

Other comments
Page 2.
Line 5. Does “fates of phytoplankton” refer to phytoplankton throughout the Southern Ocean? Are the results of this study meant to be generalizable to the whole system?
Line 8. By difference from what? What is being subtracted from what?
Line 9. Change “accounts” to “accounted”
Line 10. Remove two commas

Page 3
Line 8. I’m not sure that I would describe this as a breakthrough, but it is certainly a step forward.

Page 4
I don’t agree with the way the equations were set up. I would suggest
chl* = 1.75 * deltaNO3 (eq 1)
chl = chl* - total losses (eq 2)
Total losses = local losses + export (eq. 3)
I think this is much more intuitive and easier to understand.

Page 5
How were depth related to changes in Chl:POC due to photoacclimation taken into account?

Page 6
Lines 8-20. This is repeated from a few lines earlier.

Page 8
Lines 18-20. Would large feeding events by krill be observable in the BCG-Argo data? Might their data also suggest that patchy krill feeding is the exception, rather than the rule in areas covered by the BGC-Argo floats they used?

Page 9
Lines 12-13. I’m not sure why this sentence is included here. While LIDAR may increase the time each year over which polar oceans can be sampled, using a single channel LIDAR to measure phytoplankton concentration really creates more problems than it solves.

Line 17. Where does this 10-day timescale come from?


Reviewer #3 (Remarks to the Author):

I've spent several weeks reading this paper, thinking about it, and checking references. The manuscript seeks to link time series of autonomously measured chlorophyll fluorescence, scattering (a proxy of POC), and NO3 to arrive at an estimate for particle export in waters south of the polar front zone surrounding Antarctica. The data analyzed comes from 6 SOCCOM/BGC floats deployed at various locations around Antarctica.

I feel that the paper belongs in a journal where there is sufficient length to properly develop the analysis presented. I don't recommend publication of this paper in NCOMMS.

I have the following overview comments.

(1) The text and supplemental material are so cryptic that this reviewer finds it impossible to understand the methodology applied and how conclusions were drawn. The figure captions are not informative at all (e.g. what is the dashed line present in Fig 1?). Readers will have the same difficulty.

(2) Methodology lacks clarity. The short length of the paper forces this.

(3) There is little or no development of the hydrographic context of the float observations. Others have done a far better job. This is required.

(4) The authors will confuse the reader on spatial and temporal domains. e.g. Fig 4 maps export spatially, yet export is strongly tied in the temporal domain. Which is it? More development of the temporal timing of the bloom and export - in a hydrographic context - would help the paper.

(5) I read ref 20 carefully, and nowhere in the reported data was I able to find a way to calculate a Chla:N ratio of 1.75. The notion that Chl:N data from the coastal iron-replete Amundsen Sea is representative of offshore waters which are often iron limited must be defended. Are the phytoplankton species the same? Also the authors must defend a temporally constant CHla:N.

(6) Spikes in sensor data are very difficult to calibrate because the instrument encounters large particles that induce these spikes for fractions of a second and sensor results from these floats is highly averaged. To my knowledge, there are no published works demonstrating the quantitative calibration of such data.

(7) finally, other autonomous float-based observations of export have been reported for the southern ocean, including in the sea ice affected waters. A search of the published literature would be justified. I'm thinking of papers deriving from the SOFeX era.

**Author rebuttal, first version:**

Reviewer #1 (Remarks to the Author):

This paper uses biogeochemical ARGO floats to understand the fate of phytoplankton in the Seasonal Ice Zone of the Southern Ocean. The study is novel in that it attempts to use the data to evaluate not just the rates, but the fates of productivity by uncoupling grazing and sinking rates. Of note, the paper estimates that grazing accounts for 90% of the average losses of productivity. This is a novel and interesting finding. I enjoyed reading this paper very much and appreciated the unique methods to calculate loss terms. Obviously, their estimates are based on some assumption, such as a constant chl:NO3 ratio, that can be quite variable in space and time. However, I think they do a reasonable job of validating their estimates with other data sources and considering the variability between floats over space and over time throughout the growing season. I think this pushes the ball forward in terms of what can be done with float data and definitely contributes to an understanding of a complex ecosystem. It opens the door to new questions and types of data that is needed to further refine these estimates. I think this novel study deserves to be published. My main comments are largely stylistic in nature in terms of the methods, figures and the representation of the results in figures and a bit more validation.

We thank the Reviewer for the very positive comments on our paper and approach to tease apart the fates of phytoplankton in the sea ice zone of the Southern Ocean.

Regarding the figures, I think they have some of the most compelling figures tucked away in the supplemental section. I have not seen many estimate of de novo photosynthesis like Supp. Fig. A1 which shows the synthesized chlorophyll in relationship to the measured under different nitrate assumptions – this is a powerful validation of their approach. The mechanistic Supp. Fig. A2 is also quite useful in discerning the approach and conceptual framework for the study. I also thought Fig. A7 was useful in discerning where the floats are located in relationship to the entirety of the SIZ Southern Ocean. I think rearrangement of some of these figures into composites in the main article and

reduction and summaries of the some of the bar graphs would be useful in the main text.

We have modified the structure of the manuscript, and have moved material between the main text and the supplementary materials and methods. As suggested by the Reviewer, we moved the previous Fig. A1, A2 and A7 to the main text in the new version of the manuscript.

-	We moved Figure A7 to become the new Figure 1 of the manuscript. We use this figure to illustrate the oceanography of the two areas we studied, as also suggested by Reviewer 3. We added the following section to the manuscript (Page 3, line 12, changes are highlighted in bold):

***“The sea ice zone***

***We studied seven SOCCOM floats which drifted across the SIZ of the Weddell Sea and the Indian Ocean sector of the Southern Ocean, between 60°S and 69°S (Figure 1a). The Weddell Sea is a large cyclonic gyre. Northeast of the Weddell Gyre, the Southwest Indian Ridge was identified as a major topographic feature where circumpolar deep water (CDW) travels southward through the Antarctic Circumpolar Current (ACC)^1^. South of the ACC, CDW is upwelled to the surface through wind-driven divergence^2^ and is modified on its path through the Weddell Gyre (then called Warm Deep Water, WDW^3^). Five BGC-Argo floats drifted across the open waters of the eastern Weddell Gyre (Figure 1b), a region which was identified as a major carbon sink due to strong primary production^4^. The Kerguelen Plateau is a major obstacle on the eastward flow of the ACC. The bulk of the ACC passes north of the Kerguelen Plateau, while the remainder passes through the Fawn Trough (at ~56°S) or through the Princess Elizabeth Trough (at ~64°S), south of the Banzare Bank5, ^6^ (Figure 1c). Two BGC-Argo floats drifted in the highly productive SIZ, south of the Banzare bank7, ^8^. The SOCCOM***

1

***floats we studied park and drift at 1000 m, and descend to 2000 m before returning to the surface every 10 days.”***

-	We moved the previous Figure A1 to become the new Figure 6 of the manuscript to illustrate the sensitivity of the method we employed (i.e., the fate of phytoplankton) to the ratio of chlorophyll synthesis to nitrate uptake (chl:N). As each of the three Reviewers commented on the necessity to develop a sensitivity analysis of our method to the chl:N ratio, we added the following whole section to the manuscript (Page 9-11, lines 24-4, changes are highlighted in bold):

***“Sensitivity of the method to the chl:N ratio***

***We tested the sensitivity of the calculated maximum chlorophyll concentration to chl:N ratios ranging from 0.5 to 2.5 pg chl:pmol N (Figure 6). First, we found that a lower bound of 1.25 pg chl:pmol N was appropriate for our calculations, since below this limit, the estimated synthesized chlorophyll is consistently lower than the observed chlorophyll for 100 days at the beginning of the production season (day 300 to 400 in Figure 6a-c). This finding was consistent between all seven of the floats we studied. Furthermore, we chose 2.5 pg chl:pmol N as a reasonable upper bound for our sensitivity analysis given that it was among the highest reported chl:N values reported in field studies and laboratory cultures across sites ranging from polar to tropical waters9, 10, 11, 12, 13_._***

***Next we selected an intermediate chl:N ratio of 1.75 pg chl:pmol N as our reference value. This selection was supported since it is the average chl:N ratio (1.75 ± 0.4 pg chl:pmol N) observed in the recent detailed Amundsen Sea Polynya (spanning 72.5°S to 74°S)^11^, across a primary production gradient of integrated dissolved inorganic nitrogen drawdown (ADIN) of 27 to 740 mml N m^-2^ and integrated chlorophyll from 74 to 828 mg m^-2^ (Figure 6d and supplementary Table A1). This detailed, multi-station study is ideal since it presents a time-series of nitrate depletion and concurrent accumulation of chlorophyll during a rapidly evolving water column bloom, in the virtual absence of grazers14, ^11^. There is, however, no such situation when grazers are completely absent, suggesting that our grazing estimates are possibly underestimated.***

***Furthermore, the Amundsen Sea polynya has the highest primary production rates of all Antarctic polynyas^15^. Surrounding the Amundsen Sea polynya, the Dotson and Getz Ice Shelves are among the fastest melting glaciers of Antarctica^16^, and naturally fertilise the polynya17, ^18^. Therefore, the source of dissolved iron is likely different between the Amundsen Sea polynya and the SIZ of the Southern Ocean, with possible effects on the chl:N ratio of phytoplankton. In addition, the in situ chl:N ratio is likely to vary throughout the production season and among phytoplankton species^19^. A map of the chl:N ratio (pg chl:pmol N) measured in the Amundsen Sea polynya supports this variability (Figure 6d), with a decreasing ratio eastward, towards a more developed bloom^11^.***

***To account for this potential variability, we ran a more detailed sensitivity analysis of our method to the chl:N ratio. The results of the sensitivity analysis for all floats is given in Table 1. We find that the average export of phytodetritus for all floats is 23.4 ± 2.5, 20.5 ± 2.1 and 19.4 ± 2 mg C d^-1^ for chl:N ratios of 1.25, 1.75 and 2.5 pg chl:pmol N, respectively. Similarly, average grazing for all floats is 118.5 ± 7.6, 159 ± 10.6 and 225.7 ± 15 mg C d^-1^ for chl:N ratios of 1.25, 1.75 and 2.5 pg chl:pmol N, respectively. Thus, the contribution of grazing to total chlorophyll losses is 84, 89 and 92% for varying chl:N ratios of 1.25, 1.75 and 2.5 pg chl:pmol N, confirming the prevalence of grazing on chlorophyll losses in the SIZ of the Southern Ocean.”***

-	And, as suggested by the Reviewer, we moved the previous Figure A2 to become the new Figure 4 of the manuscript to illustrate our approach schematically.

However, we believe that the bar graphs presented in the main manuscript (previously listed as Figures 2 and 3) are particularly informative regarding the spatial and seasonal variability of the fates of

2

chlorophyll we describe in this study. Therefore, we have opted to retain them in this new version of the manuscript as Figures 4 and 5.

They note that this is the first basin scale, observational estimate of grazing throughout the SIZ of the Southern Ocean, but Fig 4 in the main paper is kind of disappointing with blobs that are hard to tell superimposed on chlorophyll of similar colors and does not really provide a corresponding synthesis of grazing across the SIZ. How representative is 158 average number across the whole SIZ? Where is the SIZ in the figure?

We agree with the Reviewer that our previous Figure 4 did not present these data in the best way as it did not allow to differentiate the temporal variability from the spatial variability. This was also highlighted by Reviewer 3. Therefore, we removed the previous figure 4 from the manuscript.

The methods are a bit confusing to figure out in supplemental materials, but I gather in the nonphotochemical quenching section that daytime fluorescence was used to estimate chlorophyll. How was this done exactly? Did they use the methods of (Haëntjens et al. 2017) and how does the corresponding uncertainty factor into the values?

We thank the Reviewer for this comment. The Reviewer is correct and the chlorophyll-a data provided by the SOCCOM floats are corrected for nonphotochemical quenching as described by Johnson et al. (2017, ref. ^20^). The corresponding uncertainty is the larger of 0.12 mg m^-3^ or 37% as highlighted by Haëntjens et al. (2017, ref. ^21^).

Therefore, we removed this paragraph from the supplementary Materials and Methods and corrected the following sentence in the main manuscript (Page 4, line 23-24, changes are highlighted in bold):

*“Chlorophyll concentration (chl) is measured by a calibrated in vivo fluorescence sensor on the BGC-Argo float,* ***corrected for nonphotochemical quenching^22^*** *and assumed to represent biomass^20^ after losses:”*

They also mention that backscattering sensors stopped working (Suppl.), but looks like there is quite a bit of data shown in Fig. 1. And, the patterns of deeper export particles from bbp look quite intriguing and different as compared to fluorescence. This is mentioned in the text (p. 4 lines 18-19). However, the next sentence say only chlorophyll is used for export. Seems like this would strengthen the export part of the paper and maybe lead to higher export values than estimate with fluorescence.

Regarding the backscattering sensors that stopped working, this was the case for almost all of the seven floats studied here. We refer the Reviewer to our previous Figure A5 (now Figure A3).

In addition, we apologize for the confusion the Reviewer experienced between chlorophyll and carbon export. In this study, we estimate two types of export: phytodetritus (from chlorophyll anomalies at depth) and carbon export (from POC spikes at depth).

In the text, we now describe the export of phytodetritus as (Page 5, lines 8-13):

*“The downward export of chlorophyll as phytodetritus is conspicuous in BGC-Argo profiles below the surface mixed layer (ML, as defined from ref. ^23^) where it corresponds to higher chlorophyll concentrations than that expected from the negligible observed NO3^-^ drawdown at these depths (Figure 2e)...*

*... Losses of chlorophyll due to downward export were calculated using conspicuous chlorophyll pulses at depth (between the ML and 400 m).”*

And we now describe the total carbon export as (Page 6, lines 4-7):

3

*“Following previous work that estimated POC export from sensors mounted on lagrangian floats24, ^25,^ 26_,_ we estimate POC export from the POC increment below the ML and down to 400 m between consecutive profiles. The downward POC export includes phytodetritus and particles transformed by the foodweb such as faecal pellets^27^, and is calculated over the same depth stratum as phytodetritus export. ”*

The Reviewers’ confusion might have arisen from the step in which we transform the export of phytodetritus to carbon units as described in the text (Page 6, lines 9-10):

*“Chlorophyll losses and biomass accumulation were converted to carbon units to assess the contribution of phytodetritus direct export to downward POC export.”*

To overcome such confusion, we moved the sentence where we describe how we measure total carbon export to Page 6 (lines 4-7), in the third section of the manuscript where we describe our method to determine the fates of phytoplankton. We hope this clarifies this section of the manuscript.

Also it is a bit unclear as to what depth “export” is defined. Some recent work suggests the maximum winter mixed layer depth is really what constrains export rather than an arbitrary depth horizon (Palevsky and Doney 2018). Is 100 m or 400 m used here? How does the depth horizon chosen influence export results?

We thank the Reviewer for this interesting comment and the reference. We agree with the Reviewer that the maximum winter mixed layer depth (MLD) is often what constrains export. And, in this study, we chose to estimate export at a depth horizon of 400 m deep, which is similar to the depth of the maximum winter MLD in the Southern Ocean^28^.

Using the depth of the maximum winter MLD as the depth horizon is the best design to estimate export on annual or longer time scale, as a part of the organic carbon that is exported below the mixed layer during the stratified summer month can be ventilated by deep winter mixing^28^. In addition, since our study is focused on the sea ice zone of the Southern Ocean, where mixing is deep in winter, we consider that our choice of 400 m as a horizon depth will give conservative export estimates.

We have added the following sentence to the manuscript to account for this interesting comment (Page 5, lines 12-16, changes are highlighted in bold):

*“Losses of chlorophyll due to downward export were calculated using conspicuous chlorophyll pulses at depth (between the ML and 400 m).* ***We chose 400 m as the depth horizon to calculate export as recent studies show that the winter mixed layer is what constrains export. Some part of the organic matter that is exported below the mixed layer during the stratified summer months can be ventilated by deep winter mixing^28^.“***

Need to label the colorbars in supplemental figures and would be helpful to include figure numbers and legends with the figures.

As suggested by the Reviewer, we added labels to the color bars of the supplementary Figures.

In addition, we added legends along the figures to ease the Reviewers’ reading.

References

Haëntjens, N., E. Boss, and L. D. Talley. 2017. Revisiting Ocean Color algorithms for chlorophyll a and particulate organic carbon in the Southern Ocean using biogeochemical floats. Journal of

*4*

Geophysical Research: Oceans 122: 6583–6593.

Palevsky, H. I., and S. C. Doney. 2018. How choice of depth horizon influences the estimated spatial patterns and global magnitude of ocean carbon export flux. Geophysical Research Letters 45: 4171– 4179.

5

Reviewer #2 (Remarks to the Author):

The paper by Moreau and colleagues uses data from BGC-Argo floats to develop a carbon budget for the Southern Ocean, and from that, calculates grazing-mediated losses. The calculation assumes that absent any losses, chlorophyll increases should balance nitrate deficits. If they don’t, then this is due to losses by the sum of export and grazing. Export losses were calculated from depth profiles of chlorophyll and so the residual loss is attributed to grazing. Although mass balance approaches to calculating fluxes do not work everywhere, I believe that their use is appropriate when sampling is on a Lagrangian framework, as it generally is with BGC-Argo floats. I think it will be very useful for elucidating a number of different biogeochemical processes as our understanding of these processes increases.

We thank the Reviewer for the positive comments on our approach.

Having said that, one must be careful that the assumptions going into the calculations are correct. In the present paper, the assumption is that the Chl:N ratio for phytoplankton is 1.75 j.tg Chl:j.tmol N, and that this represents a value for chlorophyll synthesis from assimilated nitrogen without any losses. This ratio was based on measurements made during a rapidly developing bloom in the Amundsen Sea, where the authors appear to assume that no losses of phytoplankton were occurring. If correct, this assumption makes it easier to partition grazing losses from export losses based on the balance between Chl accumulation and N depletion. However, there is never a situation where there is no loss of phytoplankton, even during the exponential growth phase of the bloom. There are always some zooplankton present and feeding, even if the growth of phytoplankton far outstrips the losses to grazing. Losses can be surprisingly high at this time, but with such high phytoplankton growth rates, it is difficult to discern. So this suggests that their estimate of grazing loss may be underestimated. The authors need to come up with a better reference value for the amount of chlorophyll synthesis expected from assimilated nitrogen. Perhaps a culture study (preferably axenic) would provide a better benchmark.

We thank the Reviewer for this constructive criticism. We agree with the Reviewer and there is never a situation where there is no loss of phytoplankton, even during the exponential growth phase of the bloom. Therefore, our estimates of grazing are possibly underestimated when using a fixed Chl:N ratio for phytoplankton of 1.75 j.tg Chl:j.tmol N. However, we also note that zooplankton biomass was particularly low in the surface mixed layer in the Amundsen Sea polynya where these detailed datasets of chlorophyll accumulation and nitrate depletion were obtained, as highlighted by Wilson et al. (2015, ref. ^14^) and Yager et al. (2016, ref. ^11^).

Therefore, we modified the following sentences to the manuscript (Page 11, lines 7-14, changes are highlighted in bold):

*“Next we selected an intermediate chl:N ratio of 1.75 µg chl:µmol N as our reference value. This selection was supported since it is the average chl:N ratio (1.75 ± 0.4 µg chl:µmol N) observed in the recent detailed Amundsen Sea Polynya (spanning 72.5°S to 74°S)^11^, across a primary production gradient of integrated dissolved inorganic nitrogen drawdown (ΔDIN) of 27 to 740 mml N m^-2^* ***and integrated chlorophyll from 74 to 828 mg m^-2^ (Figure 6d and supplementary Table A1). This detailed, multi-station study is ideal since it presents a time-series of nitrate depletion and concurrent accumulation of chlorophyll during a rapidly evolving water column bloom, in the virtual absence of grazers14, ^11^. There is, however, no such situation when grazers are completely absent, suggesting that our grazing estimates are possibly underestimated. ”***

In addition, to account for this comment and for the variability of the chl:N ratios highlighted by the three Reviewers, we added a full sensitivity analysis of the method to varying chl:N ratios. The

*6*

following sentences were added to the manuscript (Page 10-11, lines 16-4, changes are highlighted in bold):

***“Furthermore, the Amundsen Sea polynya has the highest primary production rates of all Antarctic polynyas^15^. Surrounding the Amundsen Sea polynya, the Dotson and Getz Ice Shelves are among the fastest melting glaciers of Antarctica^16^, and naturally fertilise the polynya17, ^18^. Therefore, the source of dissolved iron is likely different between the Amundsen Sea polynya and the SIZ of the Southern Ocean, with possible effects on the chl:N ratio of phytoplankton. In addition, the in situ chl:N ratio is likely to vary throughout the production season and among phytoplankton species^19^. A map of the chl:N ratio (pg chl:pmol N) measured in the Amundsen Sea polynya supports this variability (Figure 6d), with a decreasing ratio eastward, towards a more developed bloom^11^.***

***To account for this potential variability, we ran a more detailed sensitivity analysis of our method to the chl:N ratio. The results of the sensitivity analysis for all floats is given in Table 1. We find that the average export of phytodetritus for all floats is 23.4 :E 2.5, 20.5 :E 2.1 and 19.4 :E 2 mg C d^-1^ for chl:N ratios of 1.25, 1.75 and 2.5 pg chl:pmol N, respectively. Similarly, average grazing for all floats is 118.5 :E 7.6, 159 :E 10.6 and 225.7 :E 15 mg C d^-1^ for chl:N ratios of 1.25, 1.75 and 2.5 pg chl:pmol N, respectively. Thus, the contribution of grazing to total chlorophyll losses is 84, 89 and 92% for varying chl:N ratios of 1.25, 1.75 and 2.5 pg chl:pmol N, confirming the prevalence of grazing on chlorophyll losses in the SIZ of the Southern Ocean.”***

Unfortunately, we do not have a better reference value for the amount of chlorophyll synthesis expected from assimilated nitrogen, such as one from an axenic monoculture study of a polar phytoplankton species, as suggested by the Reviewer. Also, such an approach might not necessarily represent the phytoplankton community chl:N ratio. Hence we ran the sensitivity analysis of our method to the chl:N ratio as described above.

Other comments:

Page 2.

Line 5. Does “fates of phytoplankton” refer to phytoplankton throughout the Southern Ocean? Are the

results of this study meant to be generalizable to the whole system?

In that particular sentence, “fates of phytoplankton” refers to the sea ice zone of the Southern Ocean where we did our study. We clarified this sentence as (Page 2, line 6, changes are highlighted in bold):

*“Here, we determine the magnitude, timing and spatial trends of the biogeochemical (export) and ecological (foodwebs) fates of phytoplankton, based on seven BGC-Argo floats spanning three years* ***across the SIZ*** *and after ruling out advection.”*

The Reviewer is right and these results are meant to be generalizable as long as horizontal and vertical advection can be ignored as influential loss terms for chlorophyll. In our study, the changes in physical properties we observed between consecutive profiles were at least an order of magnitude smaller than the criteria developed by Johnson et al. (2017, Ref. ^29^) to successfully infer net community production from BGC-Argo floats in the Southern Ocean (see Supplementary Materials and Methods).

Therefore, we suggest that our approach can be generalized to other oceanic regions as long as the changes in physical properties between consecutive profiles are minimal. We added the following sentence to the manuscript (supplementary Materials and Methods, Page 1, lines 18-19, changes are highlighted in bold):

*“****Furthermore, we suggest that this method can be generalized to other oceanic regions as long as the changes in physical properties between consecutive profiles are minimal****.”*

***7***

Line 8. By difference from what? What is being subtracted from what?

The export losses are subtracted from total losses to leave grazing-mediated losses. We clarified the sentence as follows (Page 2, line 8, changes are highlighted in bold):

*“By* ***subtracting export losses****, we calculated grazing-mediated losses.”*

Line 9. Change “accounts” to “accounted”

We modified the sentence accordingly.

Line 10. Remove two commas

This was corrected.

Page 3

Line 8. I’m not sure that I would describe this as a breakthrough, but it is certainly a step forward.

We modified the sentence accordingly.

Page 4

I don’t agree with the way the equations were set up. I would suggest

chl* = 1.75 * deltaNO3 (eq 1)

chl = chl* - total losses (eq 2)

Total losses = local losses + export (eq. 3)

I think this is much more intuitive and easier to understand.

We modified the equations as suggested by the Reviewer, and slightly modified the text accordingly.

Page 5

How were depth related to changes in Chl:POC due to photoacclimation taken into account?

To convert Chlorophyll losses and biomass accumulation to carbon, we used a robust method to derive phytoplankton carbon from chl^30^,^31^ (referred to as T17 for Thomalla et al., 2017).

This method was specifically derived from multiple Southern Ocean glider transects^31^. The authors applied the method of Sathyendranath et al. (2009, ref. ^30^) to the Subantarctic zone of the Southern Ocean. Sathyendranath et al. (2009) considered that POC incorporates all types of particulate carbon in the system (including phytoplankton, bacteria, detritus, and viruses). Their method then assumes that for any given chlorophyll concentration, the minimum particulate carbon content represents an upper bound on phytoplankton carbon. Using co-located POC and chlorophyll data, they used a 1% quantile regression to represent the upper limit on carbon content from phytoplankton alone.

The relationship derived from Southern Ocean *in situ* data by Thomalla et al. (2017) is: *Cphyto* = 42 * *chl ^0.86^* where *Cphyto* is phytoplankton carbon.

The Reviewer is correct that this relationship does not allow for varying Chl:POC ratios depending on environmental conditions. However, Thomalla et al. (2017) showed that the method of Sathyendranath et al. (2009) and two other methods based on backscattering measurements of POC (Behrenfeld et al., 2005; ref. ^32^) yielded consistent Chl:POC ratios in the Subantarctic zone of the Southern Ocean. This highlighted the robustness of the Sathyendranath et al. (2009) method in converting chlorophyll to phytoplankton carbon in the absence of particulate backscattering.

8

Therefore, where we describe this method, we modified the following sentence to account for its inability to represent varying Chl:POC ratios depending on environmental conditions (Page 6, line 15­17, changes are highlighted in bold):

*“We used approach 2 from T17 for our reference value, since it was specifically derived from multiple Southern Ocean glider transects^31^.* ***This yielded POC:chl ratios consistent with other approaches based on backscattering^32^, but it does not account for varying POC:chl ratios with environmental conditions.”***

Page 6

Lines 8-20. This is repeated from a few lines earlier.

We believe the Reviewer is referring to the following sentence: *“The direct export of phytodetritus is calculated from increments in phytodetritus below the ML and down to 400 m between consecutive profiles.”*

As noticed by the Reviewer, this was described a few lines earlier (Page 5, line 12-13). Hence, we removed that sentence from the manuscript.

Page 8

Lines 18-20. Would large feeding events by krill be observable in the BCG-Argo data? Might their data also suggest that patchy krill feeding is the exception, rather than the rule in areas covered by the BGC-Argo floats they used?

Any feeding events by krill would arguably be observable in the BGC-Argo data as it is using nitrate drawdown to estimate chlorophyll losses. Rather than suggesting that patchy krill feeding is the exception in the SIZ of the Southern Ocean, our data may instead reflect that other type of herbivory such as copepods-dominated herbivory dominate where krill herbivory is absent33, ^34^. Likewise, the uniformity of grazing pressure might suggest that on longer timescales the krill patchiness may be averaged out, possibly due to the circumpolar advection of krill patches^35^.

We modified the corresponding paragraph accordingly (Page 9, Lines 7-22, changes are highlighted in bold):

*“This evidence of widespread and consistent seasonality in the phytodetritus and herbivory loss terms is striking but has a number of plausible explanations. First, since this study is focused in the Southern Ocean SIZ, the major phytoplankton grazers are likely to be Antarctic krill, Euphausia superba^36^. The spatially uniform nature of the fate of blooms is inconsistent with the known patchy distribution of krill^37^. Hence, the uniformity suggests that on longer timescales the krill patchiness may be averaged out,* ***by the lateral advection of krill patches^35^****. Second, this study focuses on BGC-Argo floats that had trajectories within the latter two of the three major krill development areas which are: the Scotia Sea and Antarctic Peninsula; the eastern Weddell and Lazarev Seas; and the north of Prydz bay and the Kerguelen Plateau^38^. The constancy of chlorophyll loss terms due to herbivory across the SIZ could be linked to the ubiquitous grazing pressure of krill in these areas, which dominates losses^39^. Analysis of floats from outside of known krill development areas could confirm, or not, the extent to which phytoplankton losses are uniform across the SIZ.* ***Third, our data may alternatively reflect that copepod herbivory dominates where krill herbivory is absent33, ^34^.*** *The early season dominance of phytodetritus export as a major loss term is probably due to the similarity in the drivers of primary productivity across the SIZ, where sea ice melt in spring typically releases large amounts of dissolved and particulate iron to surface waters^40^. This nutrient delivery, coupled with a shallow meltwater- and temperature-driven mixed layer, fosters the sea-ice edge blooms in which phytoplankton growth is decoupled from grazing^41, 42^.”*

Page 9

*9*

Lines 12-13. I’m not sure why this sentence is included here. While LIDAR may increase the time each year over which polar oceans can be sampled, using a single channel LIDAR to measure phytoplankton concentration really creates more problems than it solves.

We have removed the sentence where we referred to LIDAR as suggested by the Reviewer. Line 17. Where does this 10-day timescale come from?

The 10-day timescale corresponds to the float profiling frequency. We added the following sentence to the manuscript (Page 3, line 23, changes are highlighted in bold):

***“The SOCCOM floats we studied park and drift at 1000 m, and descend to 2000 m before returning to the surface every 10 days.****”*

**10**

Reviewer #3 (Remarks to the Author):

I've spent several weeks reading this paper, thinking about it, and checking references. The manuscript seeks to link time series of autonomously measured chlorophyll fluorescence, scattering (a proxy of POC), and NO3 to arrive at an estimate for particle export in waters south of the polar front zone surrounding Antarctica. The data analyzed comes from 6 SOCCOM/BGC floats deployed at various locations around Antarctica.

I feel that the paper belongs in a journal where there is sufficient length to properly develop the analysis presented. I don't recommend publication of this paper in NCOMMS.

We thank the Reviewer for his/her comments.
I have the following overview comments.

(1) The text and supplemental material are so cryptic that this reviewer finds it impossible to understand the methodology applied and how conclusions were drawn. The figure captions are not informative at all (e.g. what is the dashed line present in Fig 1?). Readers will have the same difficulty.

Thanks to the comments of all three Reviewers, we have clarified our methodology and reorganised the sections and figures between the main text and the supplementary Materials and Methods. We believe that this was the main reason for the Reviewer’s difficulty to understand our approach. We hope that the Reviewer finds it more self-explanatory now. We refer the Reviewer to our detailed responses to all Reviewers’ comments and the new version of the manuscript.

In addition, we clarified the figure captions. We refer the Reviewer to the figures caption in this new version of the manuscript.

(2) Methodology lacks clarity. The short length of the paper forces this.

Thanks to the comments of all three Reviewers, we have clarified our methodology and reorganised sections between the main text and the supplementary Materials and Methods. We hope that the Reviewer finds the methodology clearer in this new version of the manuscript.

(3) There is little or no development of the hydrographic context of the float observations. Others have done a far better job. This is required.

We added a new section at the beginning of the manuscript to describe the oceanographic context where the floats observations were made (Page 3, line 12-24, changes are highlighted in bold).

***“The sea ice zone***

***We studied seven SOCCOM floats which drifted across the SIZ of the Weddell Sea and the Indian Ocean sector of the Southern Ocean, between 60°S and 69°S (Figure 1a). The Weddell Sea is a large cyclonic gyre. Northeast of the Weddell Gyre, the Southwest Indian Ridge was identified as a major topographic feature where circumpolar deep water (CDW) travels southward through the Antarctic Circumpolar Current (ACC)^1^. South of the ACC, CDW is upwelled to the surface through wind-driven divergence^2^ and is modified on its path through the Weddell Gyre (then called Warm Deep Water, WDW^3^). Five BGC-Argo floats drifted across the open waters of the eastern Weddell Gyre (Figure 1b), a region which was identified as a major carbon sink due to strong primary production^4^. The Kerguelen Plateau is a major obstacle on the eastward flow of the ACC. The bulk of the ACC passes north of the Kerguelen Plateau, while the remainder passes through the Fawn Trough (at ~56°S) or through the Princess Elizabeth Trough (at ~64°S), south of the Banzare Bank5, ^6^ (Figure 1c). Two BGC-Argo floats drifted in the highly productive SIZ, south of the Banzare bank7, ^8^. The SOCCOM***

**11**

***floats we studied park and drift at 1000 m, and descend to 2000 m before returning to the surface every 10 days.”***

In addition, we moved Figure A7 to become the new Figure 1 of the manuscript to illustrate this section, as also suggested by Reviewer 1.

(4) The authors will confuse the reader on spatial and temporal domains. e.g. Fig 4 maps export spatially, yet export is strongly tied in the temporal domain. Which is it? More development of the temporal timing of the bloom and export - in a hydrographic context - would help the paper.

We agree with the Reviewer that our previous Figure 4 did not present these data in the best way as it did not allow to differentiate the temporal variability from the spatial variability. This was also highlighted by Reviewer 1. We have removed the Figure 4 from the new version of the manuscript. We now focus on the spatial and seasonal trends of the fates of phytoplankton based on the bar charts presented in Figures 4 and 5 and in Appendix figures A5, A6 and A7.

(5) I read ref 20 carefully, and nowhere in the reported data was I able to find a way to calculate a Chla:N ratio of 1.75.

We matched the integrated ΔDIN with the integrated chlorophyll for the 13 stations described in Yager et al. (2016, ref. ^11^). The method used here was not clear enough in the previous version of the manuscript. Hence, we improved the following sentence (Page 11, line 7-11, changes are highlighted in bold) and added a supplementary table where we report the integrated ΔDIN and chlorophyll data from Yager et al. (2016, ref. ^11^):

“*This selection was supported since it is the average chl:N ratio (1.75 ± 0.4 µg chl:µmol N) observed in the recent detailed Amundsen Sea Polynya (spanning 72.5°S to 74°S)^11^, across a primary production gradient of integrated dissolved inorganic nitrogen drawdown (ΔDIN) of 27 to 740 mml N m-2* ***and integrated chlorophyll from 74 to 828 mg m^-2^ (Figure 6d and supplementary Table A1).”***

The notion that Chl:N data from the coastal iron-replete Amundsen Sea is representative of offshore waters which are often iron limited must be defended. Are the phytoplankton species the same? Also the authors must defend a temporally constant Chla:N.

The Reviewer is correct and the Chl:N ratio will likely vary between the coastal iron-replete Amundsen Sea and the often iron-limited offshore waters. In addition, as noted by the Reviewer, the chl:N ratio will also likely vary with time over the production season and with phytoplankton species.

To account for these comments, we added the following sentences (Page 10, lines 16-23, changes are highlighted in bold):

***“Furthermore, the Amundsen Sea polynya has the highest primary production rates of all Antarctic polynyas^15^. Surrounding the Amundsen Sea polynya, the Dotson and Getz Ice Shelves are among the fastest melting glaciers of Antarctica^16^, and naturally fertilise the polynya17, ^18^. Therefore, the source of dissolved iron is likely different between the Amundsen Sea polynya and the SIZ of the Southern Ocean, with possible effects on the chl:N ratio of phytoplankton. In addition, the in situ chl:N ratio is likely to vary throughout the production season and among phytoplankton species^19^. A map of the chl:N ratio (µg chl:µmol N) measured in the Amundsen Sea polynya supports this variability (Figure 6d), with a decreasing ratio eastward, towards a more developed bloom^11^.”***

In addition, in response to all three Reviewers, we added a full sensitivity analysis of the method to varying chl:N ratios. A new table presents the results of this sensitivity analysis for all floats (Table 1). The following sentences were added to the manuscript (Page 10-11, lines 25-4, changes are highlighted in bold):

12

***“To account for this potential variability, we ran a more detailed sensitivity analysis of our method to the chl:N ratio. The results of the sensitivity analysis for all floats is given in Table 1. We find that the average export of phytodetritus for all floats is 23.4 :E 2.5, 20.5 :E 2.1 and 19.4 :E 2 mg C d-1 for chl:N ratios of 1.25, 1.75 and 2.5 pg chl:pmol N, respectively. Similarly, average grazing for all floats is 118.5 :E 7.6, 159 :E 10.6 and 225.7 :E 15 mg C d^-1^ for chl:N ratios of 1.25, 1.75 and 2.5 pg chl:pmol N, respectively. Thus, the contribution of grazing to total chlorophyll losses is 84, 89 and 92% for varying chl:N ratios of 1.25, 1.75 and 2.5 pg chl:pmol N, confirming the prevalence of grazing on chlorophyll losses in the SIZ of the Southern Ocean.”***

(6) Spikes in sensor data are very difficult to calibrate because the instrument encounters large particles that induce these spikes for fractions of a second and sensor results from these floats is highly averaged. To my knowledge, there are no published works demonstrating the quantitative calibration of such data.

The Reviewer is correct and this is a limitation of our study. Hence, we added the following sentence to the new version of the manuscript (Pages 6-7, lines 27-1, changes are highlighted in bold):

*“Except for the exceptionally high export pulse captured by float #9125, the maximum downward POC export was 981 mg C _m_-2 d-1 (Figure 4b), similar to previous estimates from the open Southern Ocean to the SIZ (1090 mg C m^-2^ d^-1^; ref. ^43^).* ***It should be noted that POC export from backscattering sensors on SOCCOM floats is likely underestimated as large spikes are averaged.”***

(7) finally, other autonomous float-based observations of export have been reported for the southern ocean, including in the sea ice affected waters. A search of the published literature would be justified. I'm thinking of papers deriving from the SOFeX era.

We agree with the Reviewer that numerous publications have reported carbon export estimates from autonomous float-based observations. We originally had a large number of these references in the text, most of which we deleted prior to submission due to the limitation on the number of references.

We agree with the Reviewer that such literature must be cited in this manuscript. Therefore, we added relevant references to the text (Pages 6, lines 4-7, changes are highlighted in bold):

***“Following previous work that estimated POC export from sensors mounted on lagrangian floats^24,^ 25, 26, we estimate POC export from the POC increment below the ML and down to 400 m between consecutive profiles. The downward POC export includes phytodetritus and particles transformed by the foodweb such as faecal pellets^27^, and is calculated over the same depth stratum as phytodetritus export.”***

***13***

**References**

1. Tamsitt V, Drake HF, Morrison AK, Talley LD, Dufour CO, Gray AR*, et al.* Spiraling pathways of global deep waters to the surface of the Southern Ocean. *Nature Communications* 2017, **8**(1)**:** 172.
2. Jullion L, Garabato ACN, Bacon S, Meredith MP, Brown PJ, Torres-Valdés S*, et al.* The contribution of the Weddell Gyre to the lower limb of the Global Overturning Circulation. *Journal of Geophysical Research: Oceans* 2014, **119**(6)**:** 3357-3377.
3. Ryan S, Schröder M, Huhn O, Timmermann R. On the warm inflow at the eastern boundary of the Weddell Gyre. *Deep Sea Research Part I: Oceanographic Research Papers* 2016, **107:** 70-81.
4. MacGilchrist GA, Naveira Garabato AC, Brown PJ, Jullion L, Bacon S, Bakker DCE*, et al.* Reframing the carbon cycle of the subpolar Southern Ocean. *Science Advances* 2019, **5**(8)**:** eaav6410.
5. Vivier F, Park Y-H, Sekma H, Le Sommer J. Variability of the Antarctic Circumpolar Current transport through the Fawn Trough, Kerguelen Plateau. *Deep Sea Research Part II: Topical Studies in Oceanography* 2015, **114:** 12-26.
6. Bestley S, van Wijk E, Rosenberg M, Eriksen R, Corney S, Tattersall K*, et al.* Ocean circulation and frontal structure near the southern Kerguelen Plateau: The physical context for the Kerguelen Axis ecosystem study. *Deep Sea Research Part II: Topical Studies in Oceanography* 2018.
7. Nicol S, Pauly T, Bindoff NL, Wright S, Thiele D, Hosie GW*, et al.* Ocean circulation off east Antarctica affects ecosystem structure and sea-ice extent. *Nature* 2000, **406**(6795)**:** 504-507.
8. Tynan CT. Ecological importance of the Southern Boundary of the Antarctic Circumpolar Current. *Nature* 1998, **392**(6677)**:** 708-710.
9. Martin JH, Fitzwater SE, Gordon RM. Iron deficiency limits phytoplankton growth in Antarctic waters. *Global Biogeochemical Cycles* 1990, **4**(1)**:** 5-12.
10. Niraula MP, Casareto BE, Hanai T, Smith SL, Suzuki Y. development of carbon biomass using incubations of unaltered deep-sea water. *Eco-Engineering* 2005, **17**(2)**:** 121­131.
11. Yager P, Sherrell R, Stammerjohn S, Ducklow H, Schofield O, Ingall E*, et al.* A carbon budget for the Amundsen Sea Polynya, Antarctica: Estimating net community production and export in a highly productive polar ecosystem. *Elementa Science of the Anthropocene* 2016, **4:** 000140.

14

12. Smith REH, Cavaletto JR, Eadie BJ, Gardner WS. Growth and lipid composition of high Arctic ice algae during the spring bloom at Resolute, Northwest Territories, Canada *Marine Ecology Progress Series* 1993, **97:** 19-29.
13. Gilpin LC, Davidson K, Roberts E. The influence of changes in nitrogen: silicon ratios on diatom growth dynamics. *Journal of Sea Research* 2004, **51**(1)**:** 21-35.
14. Wilson SE, Swalethorp R, Kjellerup S, Wolverton MA, Ducklow HW, Yager PL. Meso-and macro-zooplankton community structure of the Amundsen Sea Polynya, Antarctica (Summer 2010–2011). *Elementa Science of the Anthropoccene* 2015, **3**(000033).
15. Arrigo KR, van Dijken GL, Strong AL. Environmental controls of marine productivity hot spots around Antarctica. *Journal of Geophysical Research: Oceans* 2015, **120**(8)**:** 5545-5565.
16. Rignot E, Jacobs S, Mouginot J, Scheuchl B. Ice-shelf melting around antarctica. *Science* 2013, **341**(6143)**:** 266-270.
17. St-Laurent P, Yager PL, Sherrell RM, Stammerjohn SE, Dinniman MS. Pathways and supply of dissolved iron in the Amundsen Sea (Antarctica). *Journal of Geophysical Research: Oceans* 2017, **122**(9)**:** 7135-7162.
18. Sherrell RM, Lagerström ME, Forsch KO, Stammerjohn SE, Yager PL. Dynamics of dissolved iron and other bioactive trace metals (Mn, Ni, Cu, Zn) in the Amundsen Sea Polynya, Antarctica. *Elementa Science of the Anthropoccene* 2015, **3**(000071).
19. Arrigo KR. Marine microorganisms and global nutrient cycles. *Nature* 2004, **437:** 349.
20. Johnson KS, Plant Joshua N, Coletti Luke J, Jannasch Hans W, Sakamoto Carole M, Riser Stephen C*, et al.* Biogeochemical sensor performance in the SOCCOM profiling float array. *Journal of Geophysical Research: Oceans* 2017, **122**(8)**:** 6416-6436.
21. Haëntjens N, Boss E, Talley LD. Revisiting Ocean Color algorithms for chlorophyll a and particulate organic carbon in the Southern Ocean using biogeochemical floats. *Journal of Geophysical Research: Oceans* 2017, **122**(8)**:** 6583-6593.
22. Xing X, Morel A, Claustre H, Antoine D, D'Ortenzio F, Poteau A*, et al.* Combined processing and mutual interpretation of radiometry and fluorimetry from autonomous profiling Bio-Argo floats: Chlorophyll a retrieval. *Journal of Geophysical Research: Oceans* 2011, **116**(C6).
23. Thomson RE, Fine IV. Estimating mixed layer depth from oceanic profile data. *Journal of Atmospheric and Oceanic Technology* 2003, **20:** 319-329.
24. Bishop JKB, Wood TJ, Davis RE, Sherman JT. Robotic Observations of Enhanced Carbon Biomass and Export at 55°S During SOFeX. *Science* 2004, **304**(5669)**:** 417.

15

25. Bishop JKB, Wood TJ. Year-round observations of carbon biomass and flux variability in the Southern Ocean. *Global Biogeochemical Cycles* 2009, **23**(2).
26. Llort J, Langlais C, Matear R, Moreau S, Lenton A, Strutton P. Evaluating Southern Ocean Carbon Eddy-Pump From Biogeochemical-Argo Floats. *Journal of Geophysical Research C: Oceans* 2018, **123:** 971– 984.
27. Turner JT. Zooplankton fecal pellets, marine snow, phytodetritus and the ocean’s biological pump. *Progress in Oceanography* 2015, **130:** 205-248.
28. Palevsky HI, Doney SC. How Choice of Depth Horizon Influences the Estimated Spatial Patterns and Global Magnitude of Ocean Carbon Export Flux. *Geophysical Research Letters* 2018, **45**(9)**:** 4171-4179.
29. Johnson KS, Plant JN, Dunne JP, Talley LD, Sarmiento JL. Annual nitrate drawdown observed by SOCCOM profiling floats and the relationship to annual net community production. *Journal of Geophysical Research: Oceans* 2017, **122**(8)**:** 6668-6683.
30. Sathyendranath S, Stuart V, Nair A, Oka K, Nakane T, Bouman H*, et al.* Carbon-to-chlorophyll ratio and growth rate of phytoplankton in the sea. *Marine Ecology Progress Series* 2009, **383:** 73-84.
31. Thomalla SJ, Ogunkoya AG, Vichi M, Swart S. Using Optical Sensors on Gliders to Estimate Phytoplankton Carbon Concentrations and Chlorophyll-to-Carbon Ratios in the Southern Ocean. *Frontiers in Marine Science* 2017, **4:** 34.
32. Behrenfeld MJ, Boss E, Siegel DA, Shea DM. Carbon-based ocean productivity and phytoplankton physiology from space. *Global Biogeochemical Cycles* 2005, **19**(1)**:** n/a-n/a.
33. Priddle J, Whitehouse MJ, Ward P, Shreeve RS, Brierley AS, Atkinson A*, et al.* Biogeochemistry of a Southern Ocean plankton ecosystem: Using natural variability in community composition to study the role of metazooplankton in carbon and nitrogen cycles. *Journal of Geophysical Research: Oceans* 2003, **108**(C4).
34. Smetacek V, Assmy P, Henjes J. The role of grazing in structuring Southern Ocean pelagic ecosystems and biogeochemical cycles. *Antarctic Science* 2004, **16**(4)**:** 541­558.
35. Thorpe SE, Murphy EJ, Watkins JL. Circumpolar connections between Antarctic krill (Euphausia superba Dana) populations: Investigating the roles of ocean and sea ice transport. *Deep Sea Research Part I: Oceanographic Research Papers* 2007, **54**(5)**:** 792-810.

16

36. Atkinson A, Ward P, Hunt BPV, Pakhomov EA, Hosie GW. An overview of Southern Ocean zooplankton data: abundance, biomass, feeding and functional relationships; 2012
37. Atkinson A, Siegel V, Pakhomov EA, Jessopp MJ, Loeb V. A re-appraisal of the total biomass and annual production of Antarctic krill. *Deep Sea Research Part I: Oceanographic Research Papers* 2009, **56**(5)**:** 727-740.
38. Murphy EJ, Thorpe SE, Tarling GA, Watkins JL, Fielding S, Underwood P. Restricted regions of enhanced growth of Antarctic krill in the circumpolar Southern Ocean. *Scientific Reports* 2017, **7**(1)**:** 6963.
39. Whitehouse MJ, Atkinson A, Ward P, Korb RE, Rothery P, Fielding S. Role of krill versus bottom-up factors in controlling phytoplankton biomass in the northern Antarctic waters of South Georgia. *Marine Ecology Progress Series* 2009, **393:** 69-82.
40. Lannuzel D, Vancoppenolle M, van der Merwe P, de Jong J, Meiners KM, Grotti M*, et al.* Iron in sea ice: Review and new insights. *Elementa: Science of the Anthropocene* 2016, **4**(000130).
41. Smith Jr WO, Comiso JC. Influence of sea ice on primary production in the Southern Ocean: A satellite perspective. *Journal of Geophysical Research C: Oceans* 2008, **113**(5)**:** 2156-2202.
42. Strutton PG, Brian Griffiths F, Waters RL, Wright SW, Bindoff NL. Primary productivity off the coast of East Antarctica (80–150°E): January to March 1996. *Deep Sea Research Part II: Topical Studies in Oceanography* 2000, **47**(12)**:** 2327-2362.
43. Le Moigne FAC, Henson SA, Sanders RJ, Madsen E. Global database of surface ocean particulate organic carbon export fluxes diagnosed from the <sup>234</sup>Th technique. *Earth Syst Sci Data* 2013, **5**(2)**:** 295-304.

**Reviewer comments, second version:**

Reviewer #1 (Remarks to the Author):

I felt that the authors really took my comments seriously and did an excellent job revising the manuscripts text and figures and clarifying the methods. I think the new sensitivity analysis also amplifies the strength of their results.


Reviewer #2 (Remarks to the Author):

I have read both the authors' responses to my earlier comments and the revised manuscript and am satisfied that all of my earlier comments have been adequately addressed. It is my opinion that this paper is now suitable for publication.


Reviewer #3 (Remarks to the Author):

The authors have addressed in whole or in part most of the review criticisms. I agree with the addition of figures and the restructuring. While, I do find the manuscript far more coherent, I still believe that this manuscript is beyond the scope of NCOMMS, e.g., a tightly posed analysis and clear result. The logic used in export calculations is flawed. Most importantly, the analysis must be in greater detail and that can only be achieved in a specialist journal.

There are several points the authors have failed to address and several that have cropped up during this second look.

1) The calculation of “Export” through the integration of Chlorophyll and POC from the base of the Mixed layer to 400 m is flawed. The correct shallow limit for integration is the Critical Depth which the authors should calculate. The use of 400 m as the deeper limit for integration is simply wrong in the context of the ensemble of 7 floats; In these observations all floats were operating in salinity stratified waters south of the Antarctic Polar Front (APF) and winter ML averaged 123 m (min 80- max 170 m; Supplement; see also timeseries in Ref 40). For this reason there is zero justification for the integration of Delta NO3, fluorescence ‘spikes’ or scattering based ‘POC’ anomaly to 400m. This analysis requires a redo.

2) The integration of sub ML temporal POC concentration changes cannot be a quantitative measure of flux. Particle concentrations in the subsurface reflect the balance between sources and sinks. Most particulates contributing to particle concentration are in the small size fraction and thus do not reflect the particles that are sinking. The source of material to sub-euphotic zone POC is the fragmentation of aggregate material and sinking, but the latter is minor; the sink of this material is bacterial metabolism and re-aggregation. At best, the metric the authors have developed is an index of export and not a quantitative measure of it.

3) The authors now provide an overview of the surface circulation in the area of their floats which does help. What is lacking in supplemental materials or otherwise discussed are the fundamental hydrographic variables measured by the floats, namely Temperature, Salinity, and Density. Figure 2 should include T, S, and Density – the synthetic data can be another figure or make it a 9 panel figure.

Ref 40 found that analysis of hydrographic variability provided great insight into the timing of bloom and export. The authors have an opportunity to do much more with their analysis. For example, how does the timing of stratification key into the onset of bloom and inferred systematics of export?

Some comments follow below.


a) Define “Phytodetritus”and provide a reference.

b) Introduction: It seems that Ref 40 should be acknowledged in the introduction. What did ref 40 do and how did they do it. Ref 40 was clearly motivated by many of the same questions as motivate this analysis. Then say how this analysis based on BGC floats provides a new and comprehensive view of processes in the SIZ.

c) page 4. There is still little referencing of N to Chl in SIZ phytoplankton in waters representative of the area where the floats actually operated. This must have been measured during multiple process studies of the Southern ocean. This reviewer still finds the N:Chl discussion weakly presented.

d) page 5. Line 9. Must state the numeric criterion for MLD.

e) page 5. Line 13. See major comment #1 above. Winter ML averaged 123 m and ranged from 80 to 170m; 400 m mixing applies to waters north of the PFZ which are not salinity stratified (see ref 40). I honestly think that all ‘export’ methodology needs to be explicit in the text, not hidden in supplemental materials. I don’t think the analysis is valid (see #2 above).

f) page 5. Line 9. Shallow limit for integration should be the critical depth or euphotic depth. It is not the MLD.

g) page 6. Line 4. Bishop et al. specifically did not quantify export in the way authors have done. Please reference accurately.

h) page 6. Line 21. significant figures are excessive (throughout this manuscript).

i) are the authors simply integrating spikes or are they integrating fluorescence deeper than the euphotic layer? Same question for scattering sensor results? Mid depth spikes in scattering are also attributable to zooplankton. There are multiple references on this from process studies such as NABE and VERTIGO.

j) Page 9. Line 24 onwards. This should be in a methods section. Up front and not here.

Figures:

Fig. 1. No captions for 1b and 1c, please provide. What do the dotted lines signify?

Fig. 2. Please state in the text and Caption the actual numeric criterion used for determination of MLD. Add that winter MLD for other floats varied from as deep as 170 m and as shallow as 80 m. ADD panels for T, S and density. Discuss in text these data. ADD Critical Depth!

Fig. 3. Is ‘phytodetritus’ simply the spikes or do you integrate excess chlorophyll.

Fig. 4. Increase font size of axis numbering and labels – reduce white space. (a) should be phytodetritus POC (b) should be labeled POC not Carbon. Still do not understand how this calculation is done to get “Export”. As the scattering sensor failed but fluorescence sensor did not, the number of observations contributing to (a) and (b) also needs to be plotted (OR restrict plots to times when both sensors were working).

Fig. 5. Latitudinal box plots. I don't think this adds anything to the analysis. You also want to plot mixed layer T and S. Also all plots should include space for 69S and 66S.

Fig. 6. Sensitivity… This should be upfront.

End of review

**Author rebuttal, second version:**

Reviewer #1 (Remarks to the Author):

I felt that the authors really took my comments seriously and did an excellent job revising the manuscripts text and figures and clarifying the methods. I think the new sensitivity analysis also amplifies the strength of their results.

We thank the Reviewer for this positive feedback.

Reviewer #2 (Remarks to the Author):

I have read both the authors' responses to my earlier comments and the revised manuscript and am satisfied that all of my earlier comments have been adequately addressed. It is my opinion that this paper is now suitable for publication.

We thank the Reviewer for their positive feedback.

Reviewer #3 (Remarks to the Author):

The authors have addressed in whole or in part most of the review criticisms. I agree with the addition of figures and the restructuring. While, I do find the manuscript far more coherent, I still believe that this manuscript is beyond the scope of NCOMMS, e.g., a tightly posed analysis and clear result. The logic used in export calculations is flawed. Most importantly, the analysis must be in greater detail and that can only be achieved in a specialist journal.

We acknowledge the Reviewer’s comment but defer to the Editor, who is willing to consider a revised version of the manuscript. In addition, thanks to the very interesting comments of the reviewer, we have now substantially increased our description of the methods.

Concerning the Reviewer’s comment on the logic used for export calculations, we refer the Reviewer to our detailed response below on the use of the most appropriate upper and lower depths to estimate export. We want to thank the Reviewer here for a thorough analysis of our manuscript and new method. We think that the Reviewer’s comments have improved the paper substantially.

There are several points the authors have failed to address and several that have cropped up during this second look.

1) The calculation of “Export” through the integration of Chlorophyll and POC from the base of the Mixed layer to 400 m is flawed. The correct shallow limit for integration is the Critical Depth which the authors should calculate. The use of 400 m as the deeper limit for integration is simply wrong in the context of the ensemble of 7 floats; In these observations all floats were operating in salinity stratified waters south of the Antarctic Polar Front (APF) and winter ML averaged 123 m (min 80- max 170 m; Supplement; see also timeseries in Ref 40). For this reason there is zero justification for the integration of Delta NO3, fluorescence ‘spikes’ or scattering based ‘POC’ anomaly to 400m. This analysis requires a redo.

We have now carefully considered the Reviewer’s suggestion regarding our choice of an upper and lower limit where to calculate export.

Upper Limit calculation:

First, as suggested by the Reviewer in this and the following comments, the upper limit to calculate export should be the Critical Depth or the Euphotic Zone. In our revision of the manuscript we explored the feasibility of applying the Critical depth for the ice-covered part of the Southern Ocean that we focussed on in our study.

As presented by Bishop and Wood (2009, ref. ^1^), the critical depth can be calculated as derived by Nelson and Smith (1991, ref. ^2^) for the Southern Ocean and which considers the balance between photosynthesis, community respiration and sinking losses:

$$Z_{CRIT}=\frac{\sum{PAR}_{0}}{3.78 \times{Kd}_{PAR}}$$

Where Σ PAR_0_ is the time integrated PAR at the sea surface for a 24h period (in E m-2 d-1). Bishop and Wood (2009, ref. ^1^) were able to successfully use this approach (as evidenced in detail by their Figure 6) for two floats (Carbon Explorers, CE) in permanently open ocean waters ~55S, but it appears that it was more problematic to use this metric nearer the ice edge (as suggested by their Figure 10).

For the present study, we explored the possibility of using the Critical Depth as the upper limit to calculate export. However, we found that, due to the poor coverage of satellite-derived PAR and KdPAR near the ice edge, it was more robust to use the Euphotic Zone. Therefore, for the present study, we chose to use the Euphotic Zone depth rather than the Critical Depth. We think that, near the ice edge, the euphotic zone reflects more accurately the depth over which particles may be created. In contrast, the Critical Depth may be a more powerful metrics in open waters as previously shown by Bishop and Wood (2009, ref. ^1^).

We compute the euphotic depth (Zeu) as calculated from Dierssen et al. (2000, ref. ^3^) and only when solar irradiance reaches the ocean surface (i.e., not during the polar night) as determined by year day and latitude.

Furthermore, in this new version of the manuscript, we use the deeper of either the euphotic zone or the mixed layer depth (ML) as the upper limit below which we calculate downward export. This is because we consider two cases regarding export. (1) If the mixed layer is deeper than the euphotic zone depth, then it is likely that new particles, resulting from photosynthesis, will be stirred throughout the mixed layer which could thus be considered the zone of production. (2) If the euphotic zone depth is deeper than the mixed layer, then there could be some de novo particle production occurring below the mixed layer, so the euphotic zone depth would be considered the zone of new particle production. Our approach is comparable to that applied by Dall’Olmo et al. (2014, ref. ^4^) at high latitudes (i.e., 70N). In the revised text, we acknowledge the utility of applying the critical depth to estimating the upper vertical bound of downward export in open ocean settings, as applied by Bishop and Wood (2009, ref. ^1^).

In the new version of the manuscript, we modified the following two paragraphs (Page 5, lines 10-25, changes are underlined):

*“The downward export of chlorophyll as phytodetritus (i.e., phytoplankton aggregates and senescent cells^5^) is conspicuous in BGC-Argo profiles below the export depth where it corresponds to higher (i.e., excess) chlorophyll concentrations than that expected from the negligible observed NO_3_^-^ drawdown at these depths (Figure 2e). Following Dall’Olmo et al. (2014, ref. ^4^), the export depth is taken as the deeper of either the surface mixed layer (ML, defined from ref. ^6^ as the depth at which density increased by 0.01 kg m^-3^ compared to density at the surface) and the euphotic zone depth (Zeu, defined by ref. ^7^ as the 2% light level and derived from sea surface chlorophyll concentration in Antarctic waters) when solar irradiance reaches the ocean surface (i.e., not during the polar night) as determined by year day and latitude. We acknowledge that other open water studies have used the critical depth rather than the euphotic zone depth as the upper limit below which to calculate downward export^1^. However, near the ice edge where the coverage of satellite-derived irradiance data is poor, we find that the euphotic zone reflects more accurately the depth over which particles may be created. In contrast, the Critical Depth may be a more powerful metrics in open waters as previously shown by Bishop and Wood (2009, ref. ^1^).*

*The export of phytodetritus is first observed below the export depth in early spring and is coincident with observations of particulate organic carbon (POC) export from the BGC-Argo backscattering sensors (Figure 2f).”*

In addition, throughout the manuscript, the figures and the supplementary Materials and Methods, we modified all mentions of the mixed layer to “export depth”. We refer the Reviewer to the new version of the manuscript to see all detailed changes.

--------------------------------------------------------------------------------------------------------------------------------------

Lower Limit calculation:

Second, the Reviewer is correct and a winter mixing depth of 400 m is more typical of waters north of the Polar Front Zone, as also described in Bishop and Wood (2009, ref. ^1^). Therefore, we modified the lower limit of our export calculations to the deepest winter mixing depth recorded by the seven floats we considered (i.e., 175 m), to ensure consistency in export calculations between different floats. We choose the deepest winter mixing depth (i.e., 175 m) to ensure that exported organic export could not be ventilated to the surface ocean by deep mixing.

Therefore, we modified the following paragraph of the manuscript (Page 6, lines 8-12, changes are underlined):

“*Losses of chlorophyll due to downward export were calculated using conspicuous excess chlorophyll pulses at depth (between the export depth and 175 m). We chose 175 m (the deepest winter mixed layer estimated from all seven floats) as the depth horizon to calculate export as recent studies demonstrate that the depth of the mixed layer in winter constrains downward export^8^. For example, a proportion of the organic matter that is exported below the export depth during the stratified summer months can be ventilated by deep winter mixing.*”

We modified the following sentence (Page 6, lines 14-15, changes are underlined):

*“After calculating phytodetritus downward export, local losses were calculated as the difference between total losses (between the surface and 175 m) and downward export (between the export depth and 175 m):”*

And we modified the following sentence (Page 7, lines 1-3, changes are underlined):

*“Following previous work that estimated POC export from sensors mounted on lagrangian floats^4, 9, 10, 11^, we estimate POC export from the POC increment below the export depth and down to 175 m between consecutive profiles.”*

--------------------------------------------------------------------------------------------------------------------------------------

Because we modified the upper and lower depth from which we estimate POC export and phytodetritus POC export, we modified the section figures 2, A3, A4, and A6 and their legends accordingly to include the export depth. We also modified the boxplots figures 4 and 5 and the supplementary Materials and Methods figures A8, A9 and A10.

We re-ran our sensitivity analysis of the method to the chl:N ratio. Despite the change in our method, the revised numbers are similar to those previously reported. We refer the Reviewer to the following sentences in the new version of the manuscript Page 11-12, line 24-3, changes are underlined):

*“To account for this potential variability, we ran a more detailed sensitivity analysis of our method to the chl:N ratio. The results of the sensitivity analysis for all floats is given in Table 1. We find that the average export of phytodetritus for all floats is 22 ± 3, 19 ± 3 and 18 ± 3 mg C d^-1^ for chl:N ratios of 1.25, 1.75 and 2.5 µg chl:µmol N, respectively. Similarly, average grazing for all floats is 126 ± 7, 169 ± 10 and 233 ± 13 mg C d^-1^ for chl:N ratios of 1.25, 1.75 and 2.5 µg chl:µmol N, respectively. Thus, the contribution of grazing to total chlorophyll losses is 85, 90 and 93% for varying chl:N ratios of 1.25, 1.75 and 2.5 µg chl:µmol N, confirming the prevalence of grazing on chlorophyll losses in the SIZ of the Southern Ocean.”*

Finally, we modified all the amended results throughout the manuscript. The findings from our approach essentially carry the same message, the distinct seasonality of phytoplankton fates, the dominance of grazing on all chlorophyll losses and the widespread patterns across the sea ice zone of the Southern Ocean.

We thank the Reviewer for this comment as this new diagnostic of the export depth matches well the observations of phytodetritus POC export that we determine based on excess chlorophyll compared to nitrate deficit. To account for this, we added the following sentences (Page 5-6, lines 24-6, changes are underlined):

*“The export of phytodetritus is first observed below the export depth in early spring and is coincident with observations of particulate organic carbon (POC) export from the BGC-Argo backscattering sensors (Figure 2f). This early export of phytodetritus, following the stratification of the water column, was consistently observed among all seven floats (Figure A3), in agreement with previous studies that showed that shallow transient stratification close to spring led to more favorable conditions for primary production and carbon export in the Southern Ocean^1^. In addition, the export of phytodetritus, as determined from chlorophyll excess compared to the observed NO_3_^-^ drawdown, was consistently observed below the export depth (Figure A3), while total losses (inferred from nitrate drawdown) were consistently observed above the export depth (Figure A4), reinforcing the argument that the deeper of ML and Zeu is the correct upper metrics to get at export in the SIZ of the Southern Ocean.”*

2) The integration of sub ML temporal POC concentration changes cannot be a quantitative measure of flux. Particle concentrations in the subsurface reflect the balance between sources and sinks. Most particulates contributing to particle concentration are in the small size fraction and thus do not reflect the particles that are sinking. The source of material to sub-euphotic zone POC is the fragmentation of aggregate material and sinking, but the latter is minor; the sink of this material is bacterial metabolism and re-aggregation. At best, the metric the authors have developed is an index of export and not a quantitative measure of it.

The Reviewer is correct that the major sink of small particles in the mesopelagic layer is their fragmentation and remineralization by bacteria and zooplankton. These processes account to 70 to 85% of the sinking carbon which is lost in the mesopelagic layer^12^.

In a recent research article in Science, Briggs et al. (2020, ref. ^13^) estimated that fragmentation alone can explain 49 ± 22% of the observed flux loss between 100 and 1000 m. The authors thus concluded that fragmentation is the primary process that controls the sequestration of sinking organic carbon.

Once fragmented, particulate carbon is remineralized by bacteria into dissolved organic carbon (DOC), and the Reviewer is correct that our method does not account for the DOC carbon sink. Therefore, to account for the Reviewer comment, we added the following sentences to the manuscript (Page 7, lines 6-13, changes are underlined):

*“While Dall’Olmo et al. (2014, ref. ^4^) and Briggs et al. (2020, ref. ^13^) measured the sinking of small particles through the mesopelagic layer using backscattering sensors mounted on BGC-Argo floats, Briggs et al. (2020, ref. ^13^) highlighted that particulate fragmentation was responsible for 49 ± 22% of the observed flux loss between 100 and 1000 m. Our method to estimate carbon export does not account for the bacterial remineralization of particulates into dissolved organic carbon (DOC). In addition, large backscattering spikes might be caused by the presence of zooplankton as shown by Bishop and Wood (2008, ref. ^14^), which would lead us to overestimate POC export. However, large deep backscattering spikes on SOCCOM floats are averaged, which might lead to an underestimate of POC export.”*

3) The authors now provide an overview of the surface circulation in the area of their floats which does help. What is lacking in supplemental materials or otherwise discussed are the fundamental hydrographic variables measured by the floats, namely Temperature, Salinity, and Density. Figure 2 should include T, S, and Density – the synthetic data can be another figure or make it a 9-panel figure.

We thank the Reviewer for this positive comment on the section we previously added to describe the oceanographic settings of the zones we study. As suggested by the Reviewer, we added figures of temperature and salinity (with contoured potential density isopycnals), for all floats, as supplementary Figures A1 and A2.

In addition, we now refer to these Figures in the main manuscript (Page 3, line 24-26, changes are underlined):

*“The temperature and salinity sections of all seven floats are typical of ice-covered regions, (Figures A1 and A2), with increases in mixed layer salinity under sea ice during winter and the classic thermal signature of winter water colder than -1C at 100 m deep^1^.”*

4) Ref 40 found that analysis of hydrographic variability provided great insight into the timing of bloom and export. The authors have an opportunity to do much more with their analysis. For example, how does the timing of stratification key into the onset of bloom and inferred systematics of export?

As described in our response to the major comment 1, we added the following sentences to discuss the role of post winter stratification on primary production and carbon export (Page 5-6, lines 24-2, changes are underlined):

*“The export of phytodetritus is first observed below the export depth in early spring and is coincident with observations of particulate organic carbon (POC) export from the BGC-Argo backscattering sensors (Figure 2f). This early export of phytodetritus, following the stratification of the water column, was consistently observed among all seven floats (Figure A3), in agreement with previous studies that showed that shallow transient stratification close to spring led to more favorable conditions for primary production and carbon export in the Southern Ocean^1^.”*

However, besides this clear link between post-winter stratification and carbon export, we observe phytodetritus POC export throughout the rest of the production season with no clear link to the depth of the mixed layer, as shown in our Figure A3. Therefore, we leave a further investigation of the role of the mixed layer on carbon export for a future study.

**Some comments follow below.**

a) Define “Phytodetritus”and provide a reference.

Following Rembauville et al. (2017), we defined phytodetritus as phytoplankton aggregates and senescent cells. We modified the following sentence (page 5, line 10, changes are underlined):

*“The downward export of chlorophyll as phytodetritus (i.e., phytoplankton aggregates and senescent cells^5^)…”*

b) Introduction: It seems that Ref 40 should be acknowledged in the introduction. What did ref 40 do and how did they do it. Ref 40 was clearly motivated by many of the same questions as motivate this analysis. Then say how this analysis based on BGC floats provides a new and comprehensive view of processes in the SIZ.

The study of Bishop and Wood (2009, ref. 1) is very interesting indeed, and we thank the Reviewer for pointing it out. We now reference to this study throughout our manuscript:

In the introduction, it is referenced at the end of the following sentence (Page 3, Lines 1-3, changes are underlined), before we mention how the present analysis based on BGC floats provides a new and comprehensive view of processes in the SIZ:

*“Floats can also sense the water column during the polar night and under the sea-ice where satellites cannot view^15^, providing unique time series of phytoplankton growth and particulate organic carbon export dynamics^1^. Chlorophyll concentration is routinely used to derive primary production remotely from satellite^16, 17^ but little effort has focused on the logical next step - assessing the fate of phytoplankton blooms^18^. Information on the fate of phytoplankton is valuable as it helps to partition losses due to physical (advection), biogeochemical (export) and ecological (grazing and mortality as part of pelagic foodwebs) processes^19^. The objective of this study is to assess patterns in the biogeochemical and ecological fates of primary producers as revealed through careful analysis of BGC-Argo multi-sensor datasets for seven widely distributed floats across the SIZ. Our findings on the distinctive seasonality of export versus grazing fates represents a step forward in our understanding of drivers of biogeochemistry and ecology at high latitudes, where few continuous time-series exist in open water^20^ or in the dynamic SIZ^21^.”*

This study is also added as a reference in the section where we describe the oceanographic context (Page 3-4, lines 24-1, changes are underlined):

*“The temperature and salinity sections of all seven floats are typical of ice-covered regions, (Figures A1 and A2), with increases in mixed layer salinity under sea ice during winter and the classic thermal signature of winter water colder than -1C at 100 m deep^1^.”*

Bishop and Wood (2009, ref. 1) is added as a reference in the methodology section where we describe how we estimate carbon export (Page 5, lines 13-19, changes are underlined):

*“Following Dall’Olmo et al. (2014, ref. ^4^), the export depth is taken as the deeper of either the surface mixed layer (ML, defined from ref. ^6^ as the depth at which density increased by 0.01 kg m^-3^ compared to density at the surface) and the euphotic zone depth (Zeu, defined by ref. ^7^ as the 2% light level and derived from sea surface chlorophyll concentration in Antarctic waters) when solar irradiance reaches the ocean surface (i.e., not during the polar night) as determined by year day and latitude. We acknowledge that other open water studies have used the critical depth rather than the euphotic zone depth as the upper limit below which to calculate downward export^1^.”*

And Bishop and Wood (2009, ref. 1) is added as a reference in the methodology section where we describe the phytodetritus POC export (Page 5-6, lines 26-2, changes are underlined):

*“This early export of phytodetritus, following the stratification of the water column, was consistently observed among all seven floats (Figure A3), in agreement with previous studies that showed that shallow transient stratification close to spring led to more favorable conditions for primary production and carbon export in the Southern Ocean^1^.”*

c) page 4. There is still little referencing of N to Chl in SIZ phytoplankton in waters representative of the area where the floats actually operated. This must have been measured during multiple process studies of the Southern Ocean. This reviewer still finds the N:Chl discussion weakly presented.

We added a reference of the chl:N ratio from SIZ waters similar to those where the floats drifted. This reference comes from the SOFEX-S iron-amendment mesoscale experiment (Coale et al., 2004, ref. ^22^). From their Table 1, the chl:N ratio was 2.1 µg chl:µmol N and was therefore comparable to our reference value of 1.75 µg chl:µmol N. To our knowledge, the time series from the Amundsen polynya, followed by the SOFEX-S mesoscale experiment, are the best time series to represent blooms initiated by iron replete conditions in the SIZ of the Southern Ocean.

We added the following sentence to the paragraph where we discuss the possible variability of the chl:N ratio (Page 11, lines 12-25, changes are underlined):

*“Furthermore, the Amundsen Sea polynya has the highest primary production rates of all Antarctic polynyas^23^. Surrounding the Amundsen Sea polynya, the Dotson and Getz Ice Shelves are among the fastest melting glaciers of Antarctica^24^, and naturally fertilize the polynya^25, 26^. Therefore, the source of dissolved iron is likely different between the Amundsen Sea polynya and the SIZ of the Southern Ocean, with possible effects on the chl:N ratio of phytoplankton. In comparison, the chl:N ratio reported from the SOFEX-S iron-amended mesoscale bloom in the SIZ (at 66S) averaged 2.1 µg chl:µmol N, as reported by Coale et al. (2004, ref. ^22^; their Table 1), and was therefore comparable to the chl:N ratio of 1.75 µg chl:µmol N we chose as reference value. …”*

*To account for this potential variability, we ran a more detailed sensitivity analysis of our method to the chl:N ratio. The results of the sensitivity analysis for all floats is given in Table 1. …”*

In addition, we would like to point to the Reviewer that we re-ran the full sensitivity analysis of our results to the chl:N ratio, which demonstrates the consistency of these results across the temporal and spatial scales.

d) page 5. Line 9. Must state the numeric criterion for MLD.

We added the numeric criterion used to define the MLD. The following sentence was modified as (Page 5, line 13-17, changes are underlined):

*“Following Dall’Olmo et al. (2014, ref. ^4^), the export depth is taken as the deeper of either the surface mixed layer (ML, defined from ref. ^6^ as the depth at which density increased by 0.01 kg m^-3^ compared to density at the surface) and the euphotic zone depth (Zeu, defined by ref. ^7^ as the 2% light level and derived from sea surface chlorophyll concentration in Antarctic waters) when solar irradiance reaches the ocean surface (i.e., not during the polar night) as determined by year day and latitude.”*

e) page 5. Line 13. See major comment #1 above. Winter ML averaged 123 m and ranged from 80 to 170m; 400 m mixing applies to waters north of the PFZ which are not salinity stratified (see ref 40). I honestly think that all ‘export’ methodology needs to be explicit in the text, not hidden in supplemental materials. I don’t think the analysis is valid (see #2 above).

The Reviewer is correct and a winter mixing depth of 400 m is more typical of waters north of the Polar Front Zone, as also described in Bishop and Wood (2009, ref. ^1^). Therefore, as explained above in our response to the Reviewer major comment #1, we modified the lower limit of our export calculations to the deepest winter mixing depth (i.e., 175 m), to ensure consistency in export calculations between different floats. And we also described the export methodology in more details in the manuscript. We refer the Reviewer to our detailed response to the major comment #1.

f) page 5. Line 9. Shallow limit for integration should be the critical depth or euphotic depth. It is not the MLD.

As explained in our detailed response to the Reviewer major comment #1, we also modified the upper limit in our export calculations. We refer the Reviewer to our detailed response major comment #1.

g) page 6. Line 4. Bishop et al. specifically did not quantify export in the way authors have done. Please reference accurately.

Indeed, as seen by the Reviewer, our sentence was not correct as Bishop et al. (2004) and Bishop and Wood (2009) followed a different lagrangian approach where Carbon Explorers directly estimated sedimentation at depth while drifting. Since, our approach is different, we modified the sentence and the given references to (Page 7, lines 1-3, changes are underlined):

*“Following previous work that estimated POC export from sensors mounted on lagrangian floats^4, 9, 10, 11^, we estimate POC export from the POC increment below the export depth and down to 175 m between consecutive profiles.”*

In addition, as suggested by the Reviewer, we now discuss the results of Bishop and Wood (2009) in the introduction section and throughout the manuscript. We refer the Reviewer to our detailed response to comment b).

h) page 6. Line 21. significant figures are excessive (throughout this manuscript).

We modified the number of significant figures throughout the manuscript. We refer the Reviewer to the new version of the manuscript.

i) are the authors simply integrating spikes or are they integrating fluorescence deeper than the euphotic layer? Same question for scattering sensor results? Mid depth spikes in scattering are also attributable to zooplankton. There are multiple references on this from process studies such as NABE and VERTIGO.

We thank the Reviewer for pointing at this wording issue from the previous version of the manuscript. The Reviewer is correct and we are not only integrating spikes in fluorescence and backscattering-estimated POC below the export depth. We integrate the total POC and excess fluorescence to estimate POC export and phytodetritus POC export. We clarified the manuscript as follows, mainly by removing the misleading usage of the word “spike” (Page 5, lines 10-13, changes are underlined):

“The downward export of chlorophyll as phytodetritus (i.e., phytoplankton aggregates and senescent cells^5^) is conspicuous in BGC-Argo profiles below the export depth where it corresponds to higher (i.e., excess) chlorophyll concentrations than that expected from the negligible observed NO_3_^-^ drawdown at these depths (Figure 2e).”

We also modified the following section from the supplementary materials and methods (Page 1-2, lines 24-3, changes are underlined):

*“Confirmation that deep excess chlorophyll are sinking fresh phytodetritus*

*A rationale is presented here for interpreting subsurface excess in chlorophyll as phytodetritus (such as aggregated senescent cells) sinking below the export depth. A few studies showed that diatoms may still be viable after ingestion and excretion by krill^27^, which could cast uncertainty on our assumption of observations of phytodetritus below the export depth. However, in the Indian sector of the Southern Ocean, in proximity to Crozet Island and the Kerguelen plateau, widely observed backscattering and fluorescence excess - …”*

In addition, the Reviewer is correct and large zooplankton can also be detected by the largest backscattering spikes. For instance, Rembauville et al. (2017, ref. ^5^) discarded the largest backscattering spikes from their export calculation to remove large motile organisms from the enumeration. Bishop and Wood (2008, ref. ^14^), As part of the VERtical Transport In the Global Ocean (VERTIGO) project, also demonstrated that beam attenuation spikes abundance throughout the water column closely matched the double peaked abundance distribution of zooplankton.

As pointed by the Reviewer during the previous revision of the manuscript, “*Spikes in sensor data are very difficult to calibrate because the instrument encounters large particles that induce these spikes for fractions of a second and sensor results from these floats is highly averaged. To my knowledge, there are no published works demonstrating the quantitative calibration of such data.”* In response to this previous comment of the Reviewer, we added the following sentence to the first revised version of the manuscript (Page 7, lines 12-13): *“It should be noted that POC export from backscattering sensors on SOCCOM floats is likely underestimated as large spikes are averaged.*”

In this new version of the manuscript (2^nd^ revision), we completed that sentence to account for the possible overestimation of POC export by the presence of zooplankton Page 7, line 10-13, changes are underlined):

*“In addition, large backscattering spikes might be caused by the presence of zooplankton as shown by Bishop and Wood (2008, ref. ^14^), which would lead us to overestimate POC export. However, large deep backscattering spikes on SOCCOM floats are averaged, which might lead to an underestimate of POC export.””*

j) Page 9. Line 24 onwards. This should be in a methods section. Up front and not here.

Because of the format of the journal, we would like to keep this sensitivity analysis towards the end of the paper. This allows us to describe the method clearly upfront, while referring the reader to the analysis of the method’s sensitivity at a later stage.

**Figures:** 

Fig. 1. No captions for 1b and 1c, please provide. What do the dotted lines signify?

There are captions for 1b and 1c. The Figure 1 caption reads (the captions of Figure 1b and 1c are underlined):

“*Maps of the float trajectories overlying the chlorophyll summer climatology (1997-2018) in a) and as a function of profile days in b) the Weddell Sea and c) the Indian sector of the Southern Ocean.”*

In Figure 1b and 1c, the dotted lines indicated the bathymetry. For clarity, we added the corresponding depth to these contours. We refer the Reviewer to the new Figure 1b and 1c.

Fig. 2. Please state in the text and Caption the actual numeric criterion used for determination of MLD. Add that winter MLD for other floats varied from as deep as 170 m and as shallow as 80 m. ADD panels for T, S and density. Discuss in text these data. ADD Critical Depth!

First, we added the export depth to the Figure 2 as well as the supplementary Figures A3, A4, and A6. As explained in our response to the major comment #1, we defined the export depth as the deeper of the surface mixed layer (ML, defined from ref. ^6^ as the depth at which density increased by 0.01 kg m^-3^ compared to density at the surface) and the euphotic zone depth (Zeu, derived from the sea surface chlorophyll concentration as in Dierssen et al., 2000, ref. ^3^) when solar irradiance reaches the ocean surface (i.e., not during the polar night) as determined by date and latitude.”

In addition, we stated in the text and figures’ caption the numeric criterion used to determine the export depth.

Page 5, line 13-17, (changes are underlined):

*“Following Dall’Olmo et al. (2014, ref. ^4^), the export depth is taken as the deeper of either the surface mixed layer (ML, defined from ref. ^6^ as the depth at which density increased by 0.01 kg m^-3^ compared to density at the surface) and the euphotic zone depth (Zeu, defined by ref. ^7^ as the 2% light level and derived from sea surface chlorophyll concentration in Antarctic waters) when solar irradiance reaches the ocean surface (i.e., not during the polar night) as determined by year day and latitude.”*

Page 14-15, lines 16-2 (changes are underlined): *“Thick dashed line indicates the deepest of the euphotic zone (Zeu, red circles) defined from ref.^3^, and the mixed layer depth (grey crosses and grey line) defined from ref.^6^, as the depth at which density increased by 0.01 kg m^-3^ compared to density at the surface.”*

This was also added to the captions of Figures A1, A2, A3, A4 and A6.

As suggested by the Reviewer, we also added to the figure caption that winter ML varied from as deep as 175 m and as shallow as 90 m, with an average deep winter mixed layer of 125 ± 6 m. We added the following sentence (Page 15, line 2-3, changes are underlined):

*“Winter mixed layer across all seven floats varied from 90 to 175 m deep, averaging 125 ± 6 m.”*

This was also added to the captions of Figures A1, A2, A3, A4 and A6.

Finally, as suggested by the Reviewer in the previous major comment #3, we added temperature and salinity sections (with contoured potential density isopycnals), for all floats, as supplementary Figures A1 and A2. We refer the Reviewer to our detailed response to major comment #3.

Fig. 3. Is ‘phytodetritus’ simply the spikes or do you integrate excess chlorophyll.

Phytodetritus is the integrated excess chlorophyll. It may have not been clear in the previous version of the manuscript. Hence, we modified the following sentence:

Page 15, lines 11-12 (changes are underlined): *“Below the export depth, the excess chlorophyll reveals the export of phytodetritus, whereas herbivory mainly takes place between the surface and the export depth.”*

Fig. 4. Increase font size of axis numbering and labels – reduce white space. (a) should be phytodetritus POC (b) should be labelled POC not Carbon. Still do not understand how this calculation is done to get “Export”. As the scattering sensor failed but fluorescence sensor did not, the number of observations contributing to (a) and (b) also needs to be plotted (OR restrict plots to times when both sensors were working).

For all the boxplots figures of the manuscript (Figures 4, 5, A5, A6 and A7), we modified the labelling of the panels a), b) and c) to:

a) *Downward phytodetritus POC export rates (mg C m^-2^ d^-1^)*
b) *Downward POC export rates (mg C m^-2^ d^-1^)*
c) *Phytodetritus POC contribution to total POC export (%)*

We also modified “phytodetritus export” to “phytodetritus POC export” throughout the manuscript.

In addition, for all the boxplots figures of the manuscript, we increased the font size of numbering and labels, and reduced the white space.

Finally, for all the boxplots figures of the manuscript, we specified the number of observations contributing to each box and modified the figures legends accordingly.

Fig. 5. Latitudinal box plots. I don't think this adds anything to the analysis. You also want to plot mixed layer T and S. Also all plots should include space for 69S and 66S.

We added space for 69S and 66S where we had no observations.

However, we do find that this Figure 5 gives an interesting view on the latitudinal distribution of phytoplankton fates across the SIZ. We refer the Reviewer to our section entitled “***Widespread trends in the fate of phytoplankton stocks***” (pages 8-9) where we discuss the homogeneity of phytoplankton fates across the SIZ.

In addition, given that we added temperature and salinity sections to this new version of the manuscript in response to the Reviewer major comment #3, we do not find it useful to also add mixed layer temperature and salinity on these latitudinal box plots.

Fig. 6. Sensitivity… This should be upfront.

We refer the Reviewer to our response to the previous comment j) on the position of the sensitivity analysis in the paper.

End of review

**References**

1.	Bishop JKB, Wood TJ. Year-round observations of carbon biomass and flux variability in the Southern Ocean. *Global Biogeochemical Cycles* 2009, **23**(2).

2.	Nelson DM, Smith Jr W. Sverdrup revisited: Critical depths, maximum chlorophyll levels, and the control of Southern Ocean productivity by the irradiance-mixing regime. *Limnology and Oceanography* 1991, **36**(8)**:** 1650-1661.

3.	Dierssen HM, Smith RC. Bio-optical properties and remote sensing ocean color algorithms for Antarctic Peninsula waters. *Journal of Geophysical Research* 2000, **105:** 26301-26312.

4.	Dall'Olmo G, Mork KA. Carbon export by small particles in the Norwegian Sea. *Geophysical Research Letters* 2014, **41**(8)**:** 2921-2927.

5.	Rembauville M, Briggs N, Ardyna M, Uitz J, Catala P, Penkerc'h C*, et al.* Plankton Assemblage Estimated with BGC-Argo Floats in the Southern Ocean: Implications for Seasonal Successions and Particle Export. *Journal of Geophysical Research: Oceans* 2017**:** n/a-n/a.

6.	Thomson RE, Fine IV. Estimating mixed layer depth from oceanic profile data. *Journal of Atmospheric and Oceanic Technology* 2003, **20:** 319-329.

7.	Dierssen HM, Vernet M, Smith RC. Optimizing models for remotely estimating primary production in Antarctic coastal waters. *Antarctic Science* 2000, **12**(1)**:** 20-32.

8.	Palevsky HI, Doney SC. How Choice of Depth Horizon Influences the Estimated Spatial Patterns and Global Magnitude of Ocean Carbon Export Flux. *Geophysical Research Letters* 2018, **45**(9)**:** 4171-4179.

9.	Llort J, Langlais C, Matear R, Moreau S, Lenton A, Strutton P. Evaluating Southern Ocean Carbon Eddy‐Pump From Biogeochemical‐Argo Floats. *Journal of Geophysical Research C: Oceans* 2018, **123:** 971– 984.

10.	Smetacek V, Klaas C, Strass VH, Assmy P, Montresor M, Cisewski B*, et al.* Deep carbon export from a Southern Ocean iron-fertilized diatom bloom. *Nature* 2012, **487**(7407)**:** 313-319.

11.	Poteau A, Boss E, Claustre H. Particulate concentration and seasonal dynamics in the mesopelagic ocean based on the backscattering coefficient measured with Biogeochemical-Argo floats. *Geophysical Research Letters* 2017, **44**(13)**:** 6933-6939.

12.	Henson SA, Sanders R, Madsen E. Global patterns in efficiency of particulate organic carbon export and transfer to the deep ocean. *Global Biogeochemical Cycles* 2012, **26**(1).

13.	Briggs N, Dall’Olmo G, Claustre H. Major role of particle fragmentation in regulating biological sequestration of CO&lt;sub&gt;2&lt;/sub&gt; by the oceans. *Science* 2020, **367**(6479)**:** 791.

14.	Bishop JKB, Wood TJ. Particulate matter chemistry and dynamics in the twilight zone at VERTIGO ALOHA and K2 sites. *Deep Sea Research Part I: Oceanographic Research Papers* 2008, **55**(12)**:** 1684-1706.

15.	Pope A, Wagner P, Johnson R, Shutler JD, Baeseman J, Newman L. Community review of Southern Ocean satellite data needs. *Antarctic Science* 2017, **29**(2)**:** 97-138.

16.	Behrenfeld MJ. Climate-mediated dance of the plankton. *Nature Climate Change* 2014, **4:** 880.

17.	Behrenfeld MJ, Hu Y, O/'Malley RT, Boss ES, Hostetler CA, Siegel DA*, et al.* Annual boom-bust cycles of polar phytoplankton biomass revealed by space-based lidar. *Nature Geosci* 2017, **10**(2)**:** 118-122.

18.	Siegel DA, Buesseler KO, Doney SC, Sailley SF, Behrenfeld MJ, Boyd PW. Global assessment of ocean carbon export by combining satellite observations and food-web models. *Global Biogeochemical Cycles* 2014, **28**(3)**:** 181-196.

19.	Frost BW. The role of grazing in nutrient-rich areas of the open sea. *Limnology and Oceanography* 1991, **36**(8)**:** 1616-1630.

20.	Eriksen R, Trull TW, Davies D, Jansen P, Davidson AT, Westwood K*, et al.* Seasonal succession of phytoplankton community structure from autonomous sampling at the Australian Southern Ocean Time Series (SOTS) observatory. *Marine Ecology Progress Series* 2018, **589:** 13-31.

21.	Schofield O, Saba G, Coleman K, Carvalho F, Couto N, Ducklow H*, et al.* Decadal variability in coastal phytoplankton community composition in a changing West Antarctic Peninsula. *Deep Sea Research Part I: Oceanographic Research Papers* 2017, **124:** 42-54.

22.	Coale KH, Johnson KS, Chavez FP, Buesseler KO, Barber RT, Brzezinski MA*, et al.* Southern Ocean Iron Enrichment Experiment: Carbon Cycling in High- and Low-Si Waters. *Science* 2004, **304**(5669)**:** 408.

23.	Arrigo KR, van Dijken GL, Strong AL. Environmental controls of marine productivity hot spots around Antarctica. *Journal of Geophysical Research: Oceans* 2015, **120**(8)**:** 5545-5565.

24.	Rignot E, Jacobs S, Mouginot J, Scheuchl B. Ice-shelf melting around antarctica. *Science* 2013, **341**(6143)**:** 266-270.

25.	St-Laurent P, Yager PL, Sherrell RM, Stammerjohn SE, Dinniman MS. Pathways and supply of dissolved iron in the Amundsen Sea (Antarctica). *Journal of Geophysical Research: Oceans* 2017, **122**(9)**:** 7135-7162.

26.	Sherrell RM, Lagerström ME, Forsch KO, Stammerjohn SE, Yager PL. Dynamics of dissolved iron and other bioactive trace metals (Mn, Ni, Cu, Zn) in the Amundsen Sea Polynya, Antarctica. *Elementa Science of the Anthropoccene* 2015, **3**(000071).

27.	Michels J, Dieckmann GS, Thomas DN, Schnack-Schiel SB, Krell A, Assmy P*, et al.* Short-term biogenic particle flux under late spring sea ice in the western Weddell Sea. *Deep Sea Research Part II: Topical Studies in Oceanography* 2008, **55**(8)**:** 1024-1039.

**Reviewer comments, third version:**

Reviewer #3 (Remarks to the Author):

NCOMMS_228599-2

Moreau et al. Have responded in a comprehensive way to review comments. I think the concept of using Delta NO3 to estimate Chl produced vs. measured Chl is interesting. They have adjusted MLD/Ez estimates to match the reality of the waters in which the floats have operated. All in all, the manuscript is far improved and suitable for publication. It should be published with minor revisions.

The logic of integration of Chl and scattering measures of particle concentration as a measure of export remains a difficulty with this reviewer. The authors do add caveats to the discussion of their methodology. That said, there are still zero publications on the quantification of fluorescence or scattering spikes in terms of chlorophyll and POC. This remains a weakness.

One comment regarding signal averaging needs to be revisited: if a spike occurs over a short term compared with the time interval of the sensor measurement, then the magnitude of the spike is difficult to detect and it is harder to establish the background sensor reading. The average still does accurately represent the sensor response to background particles and the spike inducing particle, thus there is likely not much bias; however, the Chl:C ratio of the material causing the spike is still an unknown. The bias could be in any direction. It is not reasonable to state that the contributions of the spikes are underestimated.

In the discussions and conclusions, the authors talk about the succession in importance of different particle classes to export: phyto-detritus, krill, and copepod fecal pellets. SOCCOM float data on spikes and Bishop et al’s data on carbon flux index only can go so far. Is there any other methodology that can be co deployed that would validate these findings? What needs to be done to advance the field?

**Author rebuttal, third version:**

Reviewer #3 (Remarks to the Author):

NCOMMS_228599-2

Moreau et al. Have responded in a comprehensive way to review comments. I think the concept of using Delta NO3 to estimate Chl produced vs. measured Chl is interesting. They have adjusted MLD/Ez estimates to match the reality of the waters in which the floats have operated. All in all, the manuscript is far improved and suitable for publication. It should be published with minor revisions.

We thank the reviewer for the positive comments on our manuscript.

The logic of integration of Chl and scattering measures of particle concentration as a measure of export remains a difficulty with this reviewer. The authors do add caveats to the discussion of their methodology. That said, there are still zero publications on the quantification of fluorescence or scattering spikes in terms of chlorophyll and POC. This remains a weakness.

We understand the reviewer’s point, which was highlighted previously. The state of the art regarding this issue is probably the recent article by Briggs et al. (2020, ref.^1^). In our previous response to the reviewer, we highlighted this weakness in the manuscript.

One comment regarding signal averaging needs to be revisited: if a spike occurs over a short term compared with the time interval of the sensor measurement, then the magnitude of the spike is difficult to detect and it is harder to establish the background sensor reading. The average still does accurately represent the sensor response to background particles and the spike inducing particle, thus there is likely not much bias; however, the Chl:C ratio of the material causing the spike is still an unknown. The bias could be in any direction. It is not reasonable to state that the contributions of the spikes are underestimated.

We thank the reviewer for this interesting comment. We agree that if the spike occurs over a short term compared with the duration of the sensor measurement, the magnitude of the spike will be averaged. However, as pointed by the reviewer the average still does accurately represent the sensor response to background particles and the spike inducing particle, thus there is likely not much bias.

Hence, as the reviewer points, it is not reasonable to state that the contributions of the spikes are underestimated. Therefore, we removed the following sentence from the manuscript (Page 6, line 7):

“However, large deep backscattering spikes on SOCCOM floats are averaged, which might lead to an underestimate of POC export.”

In addition, the fluorescence and backscatter measurements should be very close in time and space as they come from the same sensor window at almost the same time. Briggs et al. (2013, ref.^2^) estimated this time as the inverse of the sampling frequency (1 Hz) minus 50 ns (the overhead time), so approximately 1 second. Given that both fluorescence and backscattering measurements occur at the same time, we think that this will not cause a major bias in the estimation of the Chl:C ratio. In addition, we would like to point that we convert phytoplankton and phytodetritus to carbon unit using the method of Thomalla et al., 2017 (ref.^3^), which is based on measurements of chlorophyll-a, not POC.

In the discussions and conclusions, the authors talk about the succession in importance of different particle classes to export: phyto-detritus, krill, and copepod fecal pellets. SOCCOM float data on spikes and Bishop et al’s data on carbon flux index only can go so far. Is there any other methodology that can be co deployed that would validate these findings? What needs to be done to advance the field?

As suggested by Martin et al. (2020, ref.^4^), devices such as the Underwater Vision Profiler (UVP) can now be deployed on autonomous devices to count organisms from bacteria to zooplankton. The new UVP6-LP has been designed for BGC-Argo floats, from which we can track different particles classes.

We added the following sentence to the manuscript (Page 10, line 3):

“Some of the most recent BGC-Argo floats are equipped with Underwater Vision Profilers (UVP6) which will allow counting organisms from bacteria to zooplankton^4^.”

References

1.	Briggs N, Dall’Olmo G, Claustre H. Major role of particle fragmentation in regulating biological sequestration of CO&lt;sub&gt;2&lt;/sub&gt; by the oceans. *Science* 2020, **367**(6479)**:** 791.

2.	Briggs NT, Slade WH, Boss E, Perry MJ. Method for estimating mean particle size from high-frequency fluctuations in beam attenuation or scattering measurements. *Appl Opt* 2013, **52**(27)**:** 6710-6725.

3.	Thomalla SJ, Ogunkoya AG, Vichi M, Swart S. Using Optical Sensors on Gliders to Estimate Phytoplankton Carbon Concentrations and Chlorophyll-to-Carbon Ratios in the Southern Ocean. *Frontiers in Marine Science* 2017, **4:** 34.

4.	Martin A, Boyd P, Buesseler K, Cetinic I, Claustre H, Giering S*, et al.* The oceans’ twilight zone must be studied now, before it is too late. *Natue* 2020, **580**.
